# Supplementary material for: Ultrasensitive single-genome sequencing reveals strong purifying selection in acute HIV-1 infection
Source: bioRxiv. 2026 Aug 26:2026.08.21.746200. Preprint. [Version 1] doi: 10.64898/2026.08.21.746200 (PMC13628423; doi:10.64898/2026.08.21.746200)
Supplement: Supplementary file 1 [file NIHPP2026.08.21.746200v1-supplement-1.pdf]

bioRxiv preprint doi: https://doi.org/10.64898/2026.08.21.746200; this version posted August 26, 2026. The copyright holder for this preprint (which was not certified by peer review) is the author/funder. This article is a US Government work. It is not subject to copyright under 17 USC 105 and is also made available for use under a CC0 license.

SUPPLEMENTAL MATERIAL

Text S1. Viral mutation analysis modeling

Text S2. Viral tropism of CRF01_AE

File S1. Primer sequences

File S2. GenBank accessions

Table S1. Number of observed nucleotide mutations and inferred minimal single step rate

Table S2. Inferred mutation rate based on the proportion of viral genomes identical to the TF

Table S3. Summary of inferred minimum single step rate based on observed mutations, Poisson mean, and viral generation time sensitivity with assay error correction

Table S4. The frequency of APOBEC3G/F-mediated activity among G>A mutations and all observed mutations

Table S5. Predicted coreceptor tropism of inferred single transmitted/founder virus by genetic model training of known CRF01_AE genotypes

Table S6. Frequency of detected drug resistance across viral genomes that possess at least one drug resistance mutations in inferred single TF virus participants

Figure S1. Sequence validation phylogenies for inferred single TF virus participants. Neighbor-joining phylogenetic reconstructed trees for (**A**) *pol* and (**B**) *env*. Each participant identifier is indicated and branches colored, respectively. The scale is set to 1 nt.

Figure S2. Phylogenies in inferred single TF participants. (**A**) Reconstructed phylogenies for the *pol* region. (**B**) Reconstructed phylogenies for the *env* region. The red dot in the center of the viral populations indicate the inferred TF. The participant identifier is noted next to the respective phylogeny. The scale is set to 1 nt.

bioRxiv preprint doi: https://doi.org/10.64898/2026.08.21.746200; this version posted August 26, 2026. The copyright holder for this preprint (which was not certified by peer review) is the author/funder. This article is a US Government work. It is not subject to copyright under 17 USC 105 and is also made available for use under a CC0 license.

Figure S3. Phylogenies in inferred multiple TF participants. (**A**) Reconstructed phylogenies for the *pol* region. (**B**) Reconstructed phylogenies for the *env* region. The red dots in the center of the viral populations indicate the inferred TF. The proportion of viral genomes that comprise of the TF virus is noted as a percent. The participant identifier is noted next to the respective phylogeny. Long branches have been removed. The branches intermediate between the clusters reflect inferred recombinants between two TFs. The scale is set to 1 nt.

Figure S4. Examination of subpopulation in PID 3698 *pol*. (**A**) The star phylogeny as found in Supplemental Figure 2A. The dataset underwent GR-AR filtered with the two populations noted as two shades of blue. The two populations were split for each respective star phylogeny. The average pairwise distance is reported for each tree. The number of viral genomes with their respective percent that match the 'main' TF (dark blue) or subpopulation TF (light blue). The large subpopulation are a set of viral genomes that all contain a synonymous C>T change. (**B**) Hamming distance plots for the parsed datasets. Hamming distances were calculated relative to the respective inferred single TF virus. Bars indicate the number of viral genomes sequenced in unfiltered (red), GR-AR filtered (bright blue), without the large C>T population in GR-AR filtered (sky blue), and large C>T population only in GR-AR filtered (pale blue) data. The expected number of viral genomes was determined as a probability based on the Poisson fit of each respective observed dataset. The χ^2-goodness of fit test and Kolmogorov-Smirnov test are reported for all respective datasets. (**C**) Nucleotide exchange rate frequencies of populations for PID 3698. Bars colors next to the names match the data filtering as above. Data from the unfiltered observed is from **Figure 3A**.

Figure S5. Selection of non-synonymous to synonymous (d_N/d_S) mutations across sequenced HIV-1 genomes (**A**) d_N/d_S lollipop plots for the *pol* region where the shaded region highlights the (Y)MDD codons 183-186, the highly conserved tyrosine residue was not captured in all the sequencing read. Since there is only one codon for methionine, the dS is always 0. (**B**) d_N/d_S lollipop plots for the *env* region where the shaded area highlights the V3 region. Each PID is indicated on each plot. Calculated d_N/d_S values were $\log_{10}$-transformed with positive (> 1), neutral (= 1), and purifying (< 1) values indicated. Codon position is relative to the sequence length. The average dN/dS across all participants for *pol* and *env* are reported.

Figure S6. Phylogenetic reconstruction of TF viral quasi-species population during Fiebig stages II-III after filtering potential APOBEC3G/F mutations. Trees were reconstructed from

bioRxiv preprint doi: https://doi.org/10.64898/2026.08.21.746200; this version posted August 26, 2026. The copyright holder for this preprint (which was not certified by peer review) is the author/funder. This article is a US Government work. It is not subject to copyright under 17 USC 105 and is also made available for use under a CC0 license.

viral genomes in the GR-AR filtered dataset where ≥2 genomes were included thereby excluding singlets (*i.e.*, viral genomes that differed from the TF by 1 nt and found only once). The tree was rooted on the TF for *pol* and *env*. Neighbor-joining phylogenetic trees were reconstructed and are plotted to the level of diversity (where 0.004 units ~ 1nt) with respect to that participant's inferred single TF virus (serving as the root). The nodes at the leaves are colored by the number of collapsed viral genomes with 1 (dark blue), 2-4 (light blue), 5-9 (yellow), and 10+ (red). In alignment to the phylogenies, a SNP matrix (highlighter plot) is provided where changes to the inferred single TF virus are noted as: A (red), G (orange), C (blue), and T (green). The nucleotide position is relative to the absolute sequenced region. At the top of each tree is the PID and region.

Figure S7. Phylogenetic reconstruction of TF viral quasi-species population during Fiebig stages II-III including APOBEC3F/G mutations. Viral genomes that were generated from ≥2 were included thereby excluding singlets (*i.e.*, viral genomes that differed from the transmitted/founder by 1nt and found only once) where the tree was rooted on the TF for *pol* and *env*. Neighbor-joining phylogenetic trees were reconstructed and are plotted to the level of diversity with respect to that participant's inferred single TF virus (serving as the root). The nodes at the leaves are colored by the number of collapsed viral genomes with 1 (dark blue), 2-4 (light blue), 5-9 (yellow), and 10+ (red). In alignment to the phylogenies, a SNP matrix (highlighter plot) is provided where changes to the inferred single TF virus are noted as: A (red), G (orange), C (blue), and T (green). The nucleotide position is relative to the coding region sequenced in HXB2 coordinates.

Figure S8. Extracted pseudo-trees of select branches that illustrate likely effects of APOBEC3G/F activity or 'Founder Effects'. Selected inferred single TF virus participants for (**A**) *pol* and (**B**) *env*. Neighbor-joining phylogenetic trees were reconstructed and selected branches were excised for further analysis. The branches are likely artificial due to extensive APOBEC3G/F activity with the accumulation of multiple G>A mutations within GR>AR motifs. In select pseudo-trees, mutations in red indicate "reversions" to the consensus CRF01_AE. The excised branches are plotted according to the Hamming distance with respect to that participant's inferred single TF virus (serving as the root). Labeled on branches are the amino acid changes (both synonymous and non-synonymous) reported with the associated nucleotide change. The leaves of the trees have the number of viral genomes in parentheses. In PID 3698 *pol*, synonymous C>T change that would be considered a reversion to the consensus CRF01_AE demonstrates a potential 'Founder Effect". In alignment to the phylogenies, an SNP matrix

bioRxiv preprint doi: https://doi.org/10.64898/2026.08.21.746200; this version posted August 26, 2026. The copyright holder for this preprint (which was not certified by peer review) is the author/funder. This article is a US Government work. It is not subject to copyright under 17 USC 105 and is also made available for use under a CC0 license.

(highlighter plot) is provided where changes to the inferred single TF virus are noted as: A (red), G (orange), C (blue), and T (green). The nucleotide position is relative to the coding region sequenced in HXB2 coordinates. The listed mutations on the branches are relative to the amino acid change in HXB2 coordinates.

Figure S9. Representative tree illustrating pseudo-tree topology caused by APOBEC3G/F mutations. PID 3193 *pol* neighbor-joining tree with pairwise deletion for gap handling. (**A**) Complete tree with all unique viral genomes included. Majority of genomes are singlet leaves/branches (black). Pseudo-tree branching is highlighted within the overall topology (dotted bracket). (**B**) Tree topology after filtering singlet leaves/branches. Equivalent tree can be found in **Figure S7**. (**C**) Tree topology after masking all GR-AR mutations that could be due to APOBEC3G/F. Viral genomes that had GR-AR mutations collapsed as identical to the TF (red branch). An inner branching of the pseudo-tree (beige branch, #2) collapsed into a single leaf but remained 1nt different than the TF. Non-singlet branch population demonstrating an accumulation of mutations (sky blue branch, #1). The number in the parentheses indicate the number of viral genomes collapsed into the unique sequence.

Figure S10. CTL epitope mapping for CRF01_AE. Mapping of CTL epitopes from HXB2 to CRF01_AE in (A) *pol* and (B) *env*. Epitopes are mapped to within a region of 14 amino acids or less. Epitopes were designated as either specific to HLA alleles (*e.g.*, A*02; A* pink/ B* orange/ C* blue bars), polyreactive (*e.g.*, A*02/B*58; green bars), or when no MHC presenting molecule is defined the host species is note (*e.g.*, human; black bars).

Figure S11. V3 loop mutations among single TF participants. Layer one (top) is the reference V3 loop to the TF for the PID shown. Layer two (middle) is the substitution frequency relative to the TF V3 loop sequence. Layer 3 (bottom) is the mutation frequency of the amino acid residues that differ from the transmitted/founder normalized to 100%. Where complete conservation is observed, no residue is shown in the bottom panel. Each participant ID is noted for each set of panels. Amino acid results were defined as nonpolar aliphatic (green: G (Gly), A (Ala), V (Val), L (Leu), I (Ile), M (Met), P (Pro)), nonpolar aromatic (purple: F (Phe), W (Trp), Y (Tyr)), polar uncharged (black: C (Cys), S (Ser), T (Thr), N (Asn), Q (Gln)), polar positivel charged (red: K (Lys), R (Arg), H (His)), and polar negativel charged (blue: D (Asp), E (Glu)).

bioRxiv preprint doi: https://doi.org/10.64898/2026.08.21.746200; this version posted August 26, 2026. The copyright holder for this preprint (which was not certified by peer review) is the author/funder. This article is a US Government work. It is not subject to copyright under 17 USC 105 and is also made available for use under a CC0 license.

Figure S12. Illustrative map of sequenced subgenomic regions. (**A**) Amino acid sequence of *pol* from codon 184 to 261. Site of drug resistance mutations are denoted for NRTI (red) and NNRTI (bright blue). (**B**) Amino acid sequence of *env* from codon 259 to 330. The glycosite (magenta) and co-receptor engagement residues (cyan) are noted along with other features in this subgenomic regon of *env*. Residues found to be related to either R5 vs. X4 tropism are noted with a red line. APOBEC3G/F sites at the nucleotide motif level superimposed the amino acid sequence for APOBEC3G (GG>AG; black arrows) or APOBEC3F (GA>AA; grey arrows).

Table S1. Number of observed nucleotide mutations and inferred minimal single step rate

PID	*pol* (**unfiltered** dataset)[a]			*env* (**unfiltered** dataset)[a]		
	**# of genomes**	**Observed # of mutations (bp)**	**Inferred minimal single step rate (mut/site/day)**	**# of genomes**	**Observed # of mutations (bp)**	**Inferred minimal single step rate (mut/site/day)**
3928	19,251	2,921	2.82×10^{-5}	17,242	1,122	1.21×10^{-5}
3832	12,518	713	1.28×10^{-5}	12,993	508	8.79×10^{-6}
5436	7,766	763	1.83×10^{-5}	10,329	535	1.02×10^{-5}
3698[c]	6,479	864	2.48×10^{-5}			
3698[d]	8,148	2,722	6.21×10^{-5}	10,120	590	1.18×10^{-5}
6609	10,386	912	1.63×10^{-5}	13,476	1,293	1.95×10^{-5}
8123	11,339	772	1.27×10^{-5}	13,867	1,011	1.37×10^{-5}
3513	12,913	590	8.49×10^{-6}	12,459	992	1.62×10^{-5}
9114	27,884	3,857	2.57×10^{-5}	10,957	501	9.16×10^{-6}
3193	3,704	224	1.12×10^{-5}	6,914	268	7.77×10^{-6}
8522	22,675	1,544	1.27×10^{-5}	12,602	622	9.17×10^{-6}
**Median [IQR]**	**11,929 [8,053–20,107]**	**308 [259–486]**	**1.46×10^{-5} [1.23×10^{-5}–2.63×10^{-5}]**	**12,531 [10,277–13,574]**	**606 [506–1,039]**	**1.10×10^{-5} [9.07×10^{-6}–1.43×10^{-5}]**
***p*-value[f]**	**t(9)=5.00, *p*=0.0007**			**t(9)=10.47, *p*<0.0001**		
***p*-value[g]**	**t(9)=1.21 *p*=0.26**			**t(9)=2.25, *p*=0.051**		

PID	*pol* (**filtered** dataset)[b]			*env* (**filtered** dataset)[b]		
	**# of genomes**	**Observed # of mutations (bp)**	**Inferred minimal single step rate (mut/site/day)**	**# of genomes**	**Observed # of mutations (bp)**	**Inferred minimal single step rate (mut/site/day)**
3928	19,152	2,704	2.62×10^{-5}	17,220	1,065	1.15×10^{-5}
3832	12,496	660	1.19×10^{-5}	12,987	494	8.56×10^{-5}
5436	7,744	714	1.71×10^{-5}	10,319	514	9.84×10^{-5}
3698[c]	6,624	1,154	3.24×10^{-5}			
3698[d]	8,137	1,209	2.76×10^{-5}	10,115	578	1.16×10^{-5}
6609	10,362	856	1.53×10^{-5}	13,473	1,285	1.94×10^{-5}
8123	11,313	715	1.17×10^{-5}	13,855	984	1.33×10^{-5}
3513	12,902	560	8.06×10^{-5}	12,452	978	1.60×10^{-5}
9114	27,723	3,438	2.30×10^{-5}	10,944	464	8.49×10^{-6}
3193	3,692	199	1.00×10^{-5}	6,911	261	7.57×10^{-6}
8522	22,636	1,448	1.19×10^{-5}	12,602	622	9.17×10^{-6}
**Median[e] [IQR]**	**11,905 [7,464–20,023]**	**715 [635–1,762]**	**1.36×10^{-5} [1.13×10^{-5}–2.38×10^{-5}]**	**12,527 [10,268–13,569]**	**600 [487–1,004]**	**1.07×10^{-5} [8.54×10^{-6}–1.40×10^{-5}]**
***p*-value[f]**	**t(9)=4.76, *p*=0.001**			**t(9)=10.47, *p*<0.0001**		
***p*-value[g]**	**t(9)=0.14, *p*=0.89**			**t(9)=2.47, *p*=0.04**		

[a] Unfiltered dataset, where all viral genome sequences were analyzed

[b] Filtered dataset, viral genome sequences with ≥2 GR-AR (APOBEC3G/F) were removed

[c,d] Since PID 3698 has a potential 'Founder Effect', 3698[c] is where the C>T lineage sequences were removed, and 3698[d] maintains the C>T lineage viral genomes in analysis

[e] The Median and IQR were calculated with the exclusion of PID 3698[d] where the C>T lineage was present for the uncollapsed dataset

[f] PID 3698[d] dataset was excluded. Values were log_{10}-transformed; One-sample t-test compared to HIV-1 RT single step rate of 3.0×10^{-5} mut/site/day

[g] PID 3698[d] dataset was excluded. Values were log_{10}-transformed; One-sample t-test compared to HIV-1 RT single step rate of 1.4×10^{-5} mut/site/day

Table S2. Inferred mutation rate based on the proportion of viral genomes identical to the TF

PID	*pol* subgenomic region			*env* subgenomic region		
	**Proportion of TF[a]**	**Mean (λ)[b]**	**Inferred mutation rate (mut/site/day)[c]**	**Proportion of TF[a]**	**Mean (λ)[b]**	**Inferred mutation rate (mut/site/day)[c]**
3928	0.86	0.151	2.80×10^{-5}	0.94	0.062	1.15×10^{-5}
3832	0.95	0.051	1.15×10^{-5}	0.96	0.041	9.18×10^{-6}
5436	0.91	0.094	1.75×10^{-5}	0.85	0.163	3.02×10^{-5}
3698	0.87	0.139	2.59×10^{-5}	0.95	0.051	9.53×10^{-6}
6609	0.92	0.083	1.55×10^{-5}	0.91	0.094	1.75×10^{-5}
8123	0.94	0.062	1.15×10^{-5}	0.93	0.073	1.35×10^{-5}
3513	0.96	0.041	7.58×10^{-6}	0.92	0.083	1.55×10^{-5}
9114	0.87	0.139	2.59×10^{-5}	0.96	0.041	7.58×10^{-6}
3193	0.94	0.062	1.15×10^{-5}	0.96	0.041	7.58×10^{-6}
8522	0.94	0.062	1.15×10^{-5}	0.95	0.051	9.53×10^{-6}
**Median**	**0.93**	**0.73**	**1.35×10^{-5}**	**0.95**	**0.057**	**1.05×10^{-5}**
**IQR**	**0.88–0.94**	**0.062–0.128**	**1.15×10^{-5}–2.38×10^{-5}**	**0.92–0.96**	**0.043–0.081**	**9.27×10^{-6}–1.50×10^{-5}**
***p*-value[d]**			**$t(9)=4.85$, $p=0.0009$**			**$t(9)=6.73$, $p<0.0001$**
***p*-value[e]**			**$t(9)=0.61$, $p=0.57$**			**$t(9)=1.14$, $p=0.28$**

[a] Proportion of sequences that match the inferred single TF (Hamming distance of 0)
[b] Mean of a Poisson distribution when k=0 (*i.e.*, Hamming distance=0), $\lambda = -\ln(\Pr(0))$
[c] Median days corresponding to Fiebig stage used
[d] Values were $\log_{10}$-transformed; One-sample t-test compared to HIV-1 RT single step rate of 3.0×10^{-5} mut/site/day
[e] Values were $\log_{10}$-transformed; One-sample t-test compared to HIV-1 RT single step rate of 1.4×10^{-5} mut/site/day

Table S3. Summary of inferred minimal single step rate based on observed mutations, Poisson mean, and viral generation time sensitivity with assay error correction.

Dataset type	Region	Inferred minimal single step rate based on Poisson mean (λ)[a]	Observed inferred minimal single step rate[b]	HIV-1 RT error rate with viral generation time sensitivity (assay error corrected)					
				$\varepsilon = 3.0\text{x}10^{-5}$			$\varepsilon = 1.4\text{x}10^{-5}$		
				$\tau = 1.0$[c]	$\tau = 1.5$[d]	$\tau = 2.0$[e]	$\tau = 1.0$[f]	$\tau = 1.5$[g]	$\tau = 2.0$[h]
Unfiltered	*pol*	$1.35\text{x}10^{-5}$ [$1.15\text{x}10^{-5}$–$2.59\text{x}10^{-5}$]	$1.46\text{x}10^{-5}$ [$1.23\text{x}10^{-5}$–$2.63\text{x}10^{-5}$]	$4.30\text{x}10^{-5}$	$3.26\text{x}10^{-5}$	~$2.80\text{x}10^{-5}$	$2.70\text{x}10^{-5}$	$2.22\text{x}10^{-5}$	$2.03\text{x}10^{-5}$
	env	$1.10\text{x}10^{-5}$ [$8.99\text{x}10^{-6}$–$1.75\text{x}10^{-5}$]	$1.10\text{x}10^{-5}$ [$9.07\text{x}10^{-6}$–$1.43\text{x}10^{-5}$]						
Filtered	*pol*	$1.35\text{x}10^{-5}$ [$1.15\text{x}10^{-5}$–$2.59\text{x}10^{-5}$]	$1.36\text{x}10^{-5}$ [$1.13\text{x}10^{-5}$–$2.38\text{x}10^{-5}$]						
	env	$1.10\text{x}10^{-5}$ [$8.99\text{x}10^{-6}$–$1.75\text{x}10^{-5}$]	$1.07\text{x}10^{-5}$ [$8.54\text{x}10^{-6}$–$1.40\text{x}10^{-5}$]						

All values are reported as the median [interquartile range] in mut/site/day

[a] Values from **Table S2**

[b] Values from **Table S1**

[c] Values from **Text S1 table 1**

[d] Values from **Text S1 table 3**

[e] Values from **Text S1 table 5**

[f] Values from **Text S1 table 2**

[g] Values from **Text S1 table 4**

[h] Values from **Text S1 table 6**

Table S4. The frequency of APOBEC3G/F-mediated activity among G>A mutations and all observed mutations.

pol subgenomic regions								
PID	Total number of mutations	G→A mutations (%)	% G→A from APOBEC3G/F			% of all observed mutations		
			% A3G/F	% A3G	% A3F	%A3G/F	% A3G	% A3F
3928	2,921	37.8	83.6	23.6	60.0	31.6	8.9	22.7
3832	713	42.2	86.6	12.4	74.2	36.5	5.2	31.3
5436	763	64.7	61.3	8.6	52.7	39.7	5.6	34.1
3698[a]	864	19.2	78.5	17.8	60.7	15.1	3.4	11.7
3698[b]	2,722	7.9	77.1	18.0	59.0	6.1	1.4	4.6
6609	912	49.5	84.5	33.9	50.6	41.8	16.8	25.0
8123	772	51.8	81.2	11.1	70.2	42.1	5.7	36.4
3513	590	49.5	75.9	18.2	57.7	37.6	9.0	28.6
9114	3,857	33.7	91.9	30.0	62.0	31.0	10.1	20.9
3193	224	46.6	89.2	18.6	70.6	41.6	8.7	32.9
8522	1,544	34.7	71.8	16.4	55.4	24.9	5.7	19.2
**Median[c]**	**818**	**44.4**	**82.4**	**18.0**	**60.4**	**37.1**	**7.2**	**26.8**
**IQR[c]**	**682–1,888**	**34.5-50.1**	**74.9-87.3**	**12.0-25.2**	**54.7-70.3**	**29.5-41.6**	**5.5-9.3**	**20.5-33.2**
env subgenomic regions								
PID	Total number of mutations	G→A mutations (%)	% G→A from APOBEC3G/F			% of all observed mutations		
			% A3G/F	% A3G	% A3F	% A3G/F	% A3G	% A3F
3928	1,122	41.6	77.6	15.4	62.2	32.3	6.4	25.8
3832	508	43.3	57.3	12.3	45.0	24.8	5.3	19.5
5436	535	44.9	64.6	11.5	53.1	29.0	5.2	23.9
3698	590	25.4	78.7	12.5	66.2	20.0	3.2	16.8
6609	1,293	20.7	75.7	16.2	59.5	15.6	3.3	12.3
8123	1,011	57.9	94.3	66.4	27.8	54.6	38.5	16.1
3513	992	21.0	76.5	14.2	62.3	16.1	3.0	13.1
9114	501	46.1	87.2	11.9	75.3	40.2	5.5	34.7
3193	268	36.0	80.9	18.0	62.9	29.1	6.5	22.6
8522	622	29.0	78.6	19.3	59.3	22.7	5.6	17.2
**Median**	**606**	**38.8**	**78.1**	**14.8**	**60.8**	**26.9**	**5.4**	**18.3**
**IQR**	**506–1,039**	**24.3-45.2**	**72.8-82.5**	**12.2-18.3**	**51.1-63.7**	**19.0-34.2**	**3.3-6.4**	**15.4-24.4**

**% G→A from APOBECG/F: % A3G vs. % A3F *p*-value[d]**	*pol*	0.001
	env	0.003
**% observed mutations: % A3G vs. % A3F *p*-value[d]**	*pol*	0.002
	env	0.06

[a,b] Since PID 3698 has a potential 'Founder Effect', 3698[a] is where the C>T lineage sequences were removed, and 3698[b] maintains the C>T lineage viral genomes in analysis

[c] The Median and IQR were calculated with the exclusion of PID 3698[b] where the C>T lineage was present for the uncollapsed dataset

[d] Wilcoxon matched-pairs signed rank test

Table S5. Predicted coreceptor tropism of inferred single transmitted/founder virus by genetic model training of known CRF01_AE genotypes

Sequence/ Participant ID	V3 loop[a,b]	Net charge	Training model *Population % total (% unique)*		
			TF tropism	R5	X4
Con. CRF01_AE	CTRPSNNTRT**S**ITIGPGQV-FYRTG**D**IIGDIRKAY C	+3	R5	-	-
3928	CTRPNNNIRQ**S**VNIGPGQVLFYRPG**D**VIGDIRKAY(C)	+3	R5	99.10 (89.7)	0.90 (10.3)
3832	CTRPSNNTRT**S**VSIGPGQV-FYRTG**D**IIGDIKRAY(C)	+3	R5	99.99 (99.5)	0.01 (0.5)
5436	CSRPSNNTRT**S**TRIGPGQV-FYSTG**A**ITGDIRKAY(C)	+4	R5	99.94 (98.1)	0.06 (1.9)
3698	CTRPSNNTRA**S**ISIGPGRV-YYRTG**D**IIGDIRRAY(C)	+4	R5	99.62(91.6)	0.38 (8.4)
6609	CTRPANNTRT**G**ITIGPGQV-FYQTG**D**IIGDIRKAY(C)	+2	R5	100.00 (100)	0.00 (0)
8123	CTRPSNNTRT**S**THIGPGQV-FYRTG**E**IIGNIRQAY(C)	+4	R5	99.50 (88.7)	0.50 (11.3)
3513	CTRPSNNTRK**G**IHIGPGQVL-YRTD**A**IVGDIRKAY(C)	+5	R5	99.57 (88.1)	0.43 (11.9)
9114	CTRPFNNTRK**S**IHIGPGQT-FYRTG**D**IIGDIRKAY(C)	+5	X4	0.31 (8.1)	99.69 (91.9)
3193	CTRPSNNTRT**S**ITIGPGQV-FYRTG**D**IIGDIRQAH(C)	+3	R5	99.99 (99.3)	0.01 (0.7)
8522	CTRPSNNTRT**G**ITIGPGQI-FYRTG**D**IIGDIRRAY(C)	+3	R5	99.98 (99.6)	0.02 (0.4)

[a] V3 loop residues are denoted as negative charged (blue), positive charged (red), non-charged (black). HXB2 codons 254 to 331 of the amplicon sequenced. The final residue of the V3 loop (Cys, C) was not captured, a placeholder (C) is included

[b] The 11th and 25th positions are in bold

Table S6. Frequency of detected drug resistance across viral genomes that possess at least one drug resistance mutations in inferred single TF virus participants

PID	% Frequency observed among viral genomes with at least one drug resistance mutation									
	NRTI					NNRTI				
	M184I[a] AT***G***→AT***A***	M184V ***A***TG→***G***TG	L210W T***T***G→T***G***G	K219E ***A***AA→***G***AA	K219Q ***A***AA→***C***AA	Y188C T***A***T→T***G***T	Y188H ***T***AT→***C***AT	G190A G***G***A→G***C***A	G190E[b] G***G***A→G***A***A	M230L ***A***TG→***Y***TG
3928	4.03	-	0.21	0.21	-	0.21	0.21	-	1.69	0.21
3832	0.46	-	-	-	-	-	-	-	1.83	-
5436	0.97	-	-	-	-	-	-	0.49	3.40	-
3698	0.35	-	-	-	-	-	0.69	-	0.69	-
6609	1.12	-	-	-	0.37	-	-	-	1.49	-
8123	1.26	0.42	-	-	-	0.42	0.42	-	0.84	0.42
3513	1.74	-	-	0.43	-	0.43	-	0.43	1.74	-
9114	3.42	-	-	-	-	-	0.23	0.23	3.65	-
3193	-	1.04	-	-	-	-	1.04	-	1.04	-
8522	1.14	-	-	-	-	0.28	-	-	2.85	0.58

[a] Very common APOBEC3G mutation
[b] Frequent APOBEC3F mutation

Figure S1

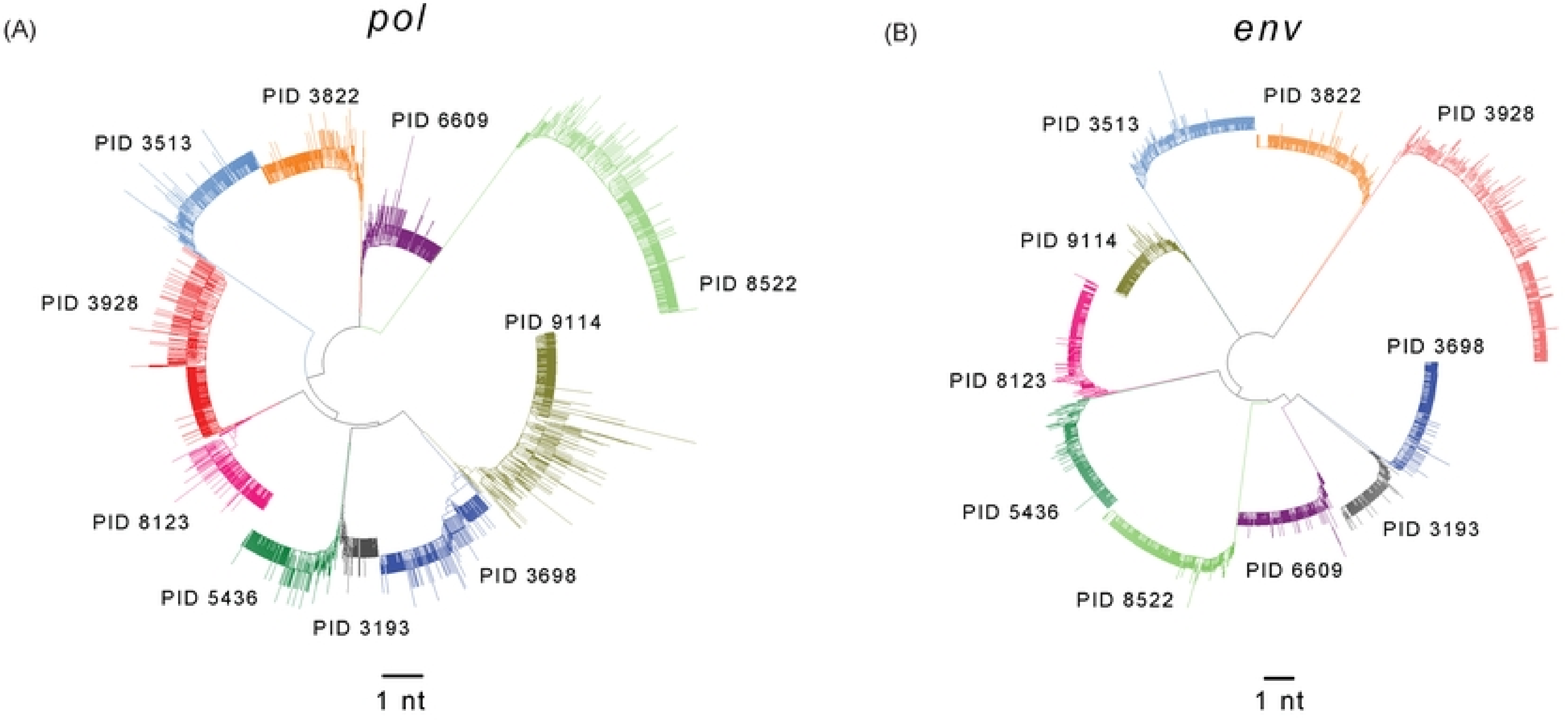

(A)
pol
PID 3822
PID 6609
PID 3513
PID 8522
PID 3928
PID 9114
PID 8123
PID 3698
PID 5436
PID 3193
1 nt
(B)
env
PID 3822
PID 3928
PID 3513
PID 9114
PID 3698
PID 8123
PID 5436
PID 3193
PID 6609
PID 8522
1 nt

Figure S2

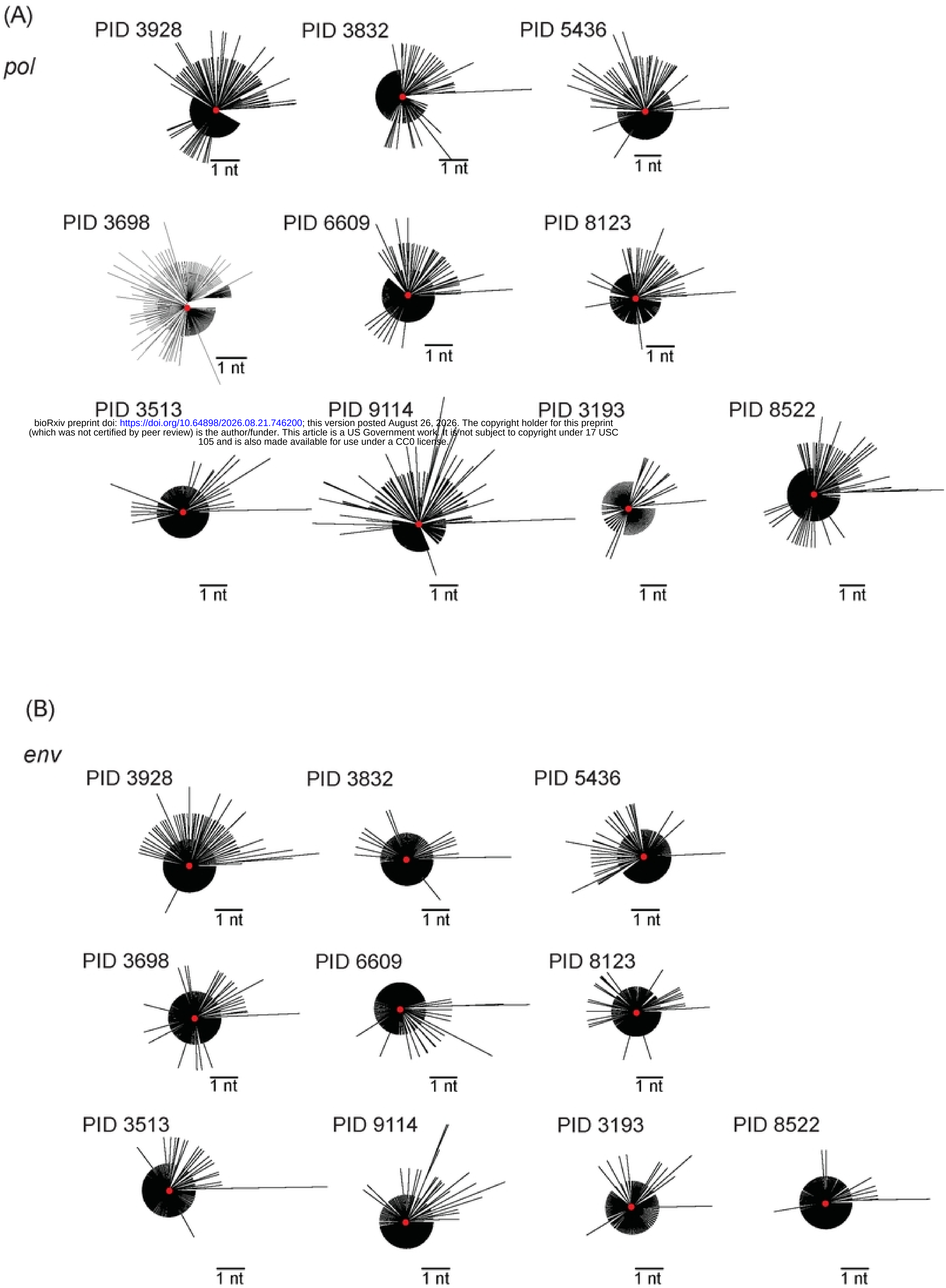

bioRxiv preprint doi: https://doi.org/10.64898/2026.08.21.746200; this version posted August 26, 2026. The copyright holder for this preprint (which was not certified by peer review) is the author/funder. This article is a US Government work. It is not subject to copyright under 17 USC 105 and is also made available for use under a CC0 license.

Figure S3

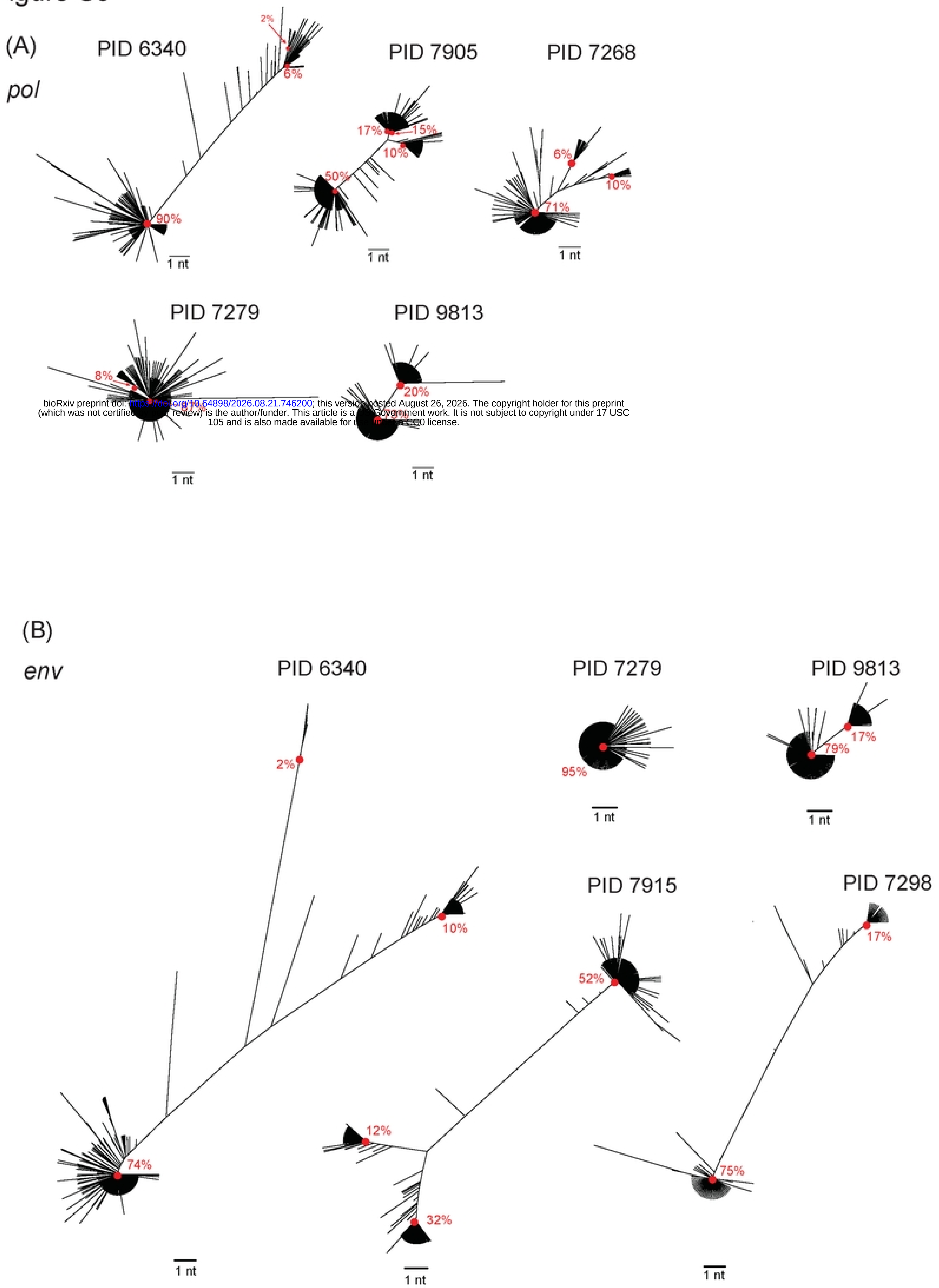

bioRxiv preprint doi: https://doi.org/10.64898/2026.08.21.746200; this version posted August 26, 2026. The copyright holder for this preprint (which was not certified by peer review) is the author/funder. This article is a US Government work. It is not subject to copyright under 17 USC 105 and is also made available for use under a CC0 license.

Figure S4

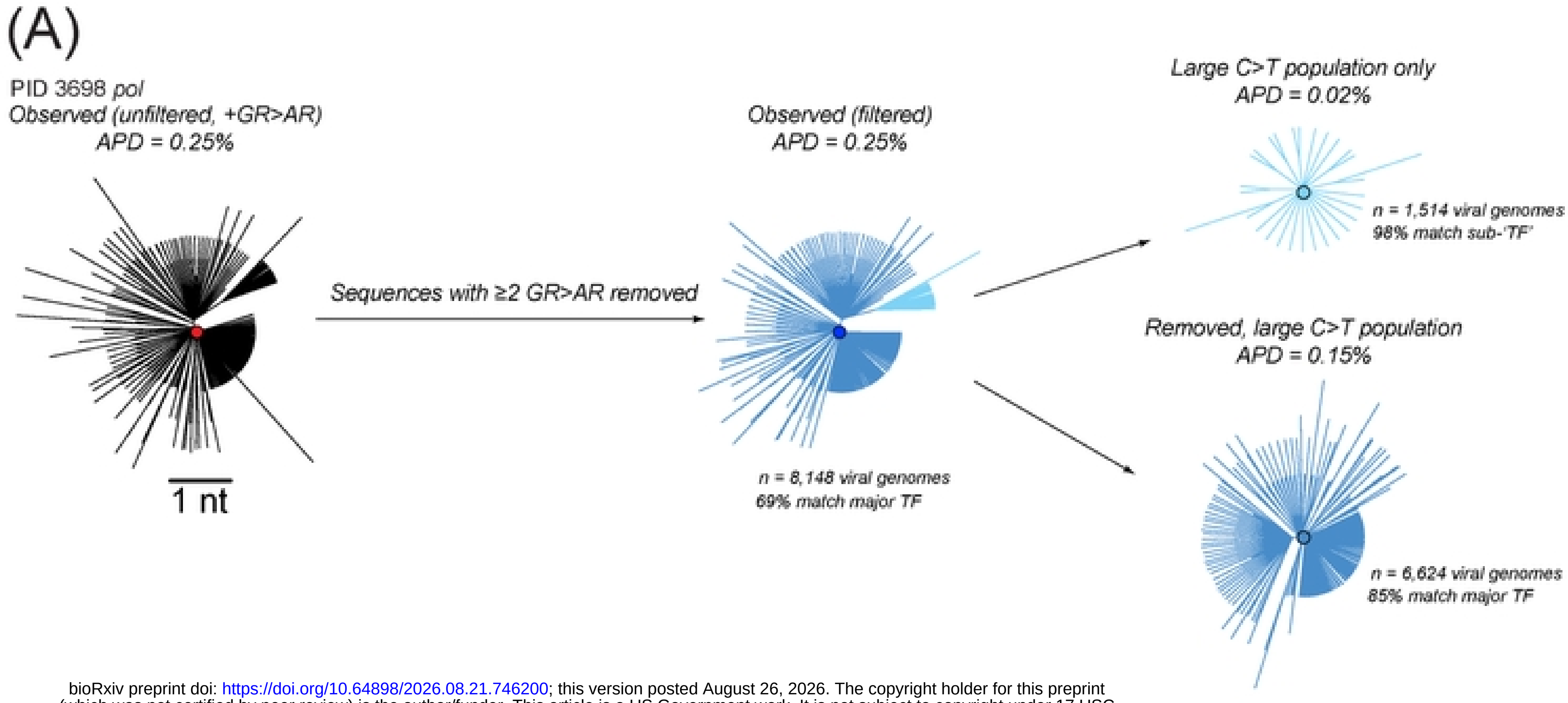

bioRxiv preprint doi: https://doi.org/10.64898/2026.08.21.746200; this version posted August 26, 2026. The copyright holder for this preprint (which was not certified by peer review) is the author/funder. This article is a US Government work. It is not subject to copyright under 17 USC 105 and is also made available for use under a CC0 license.

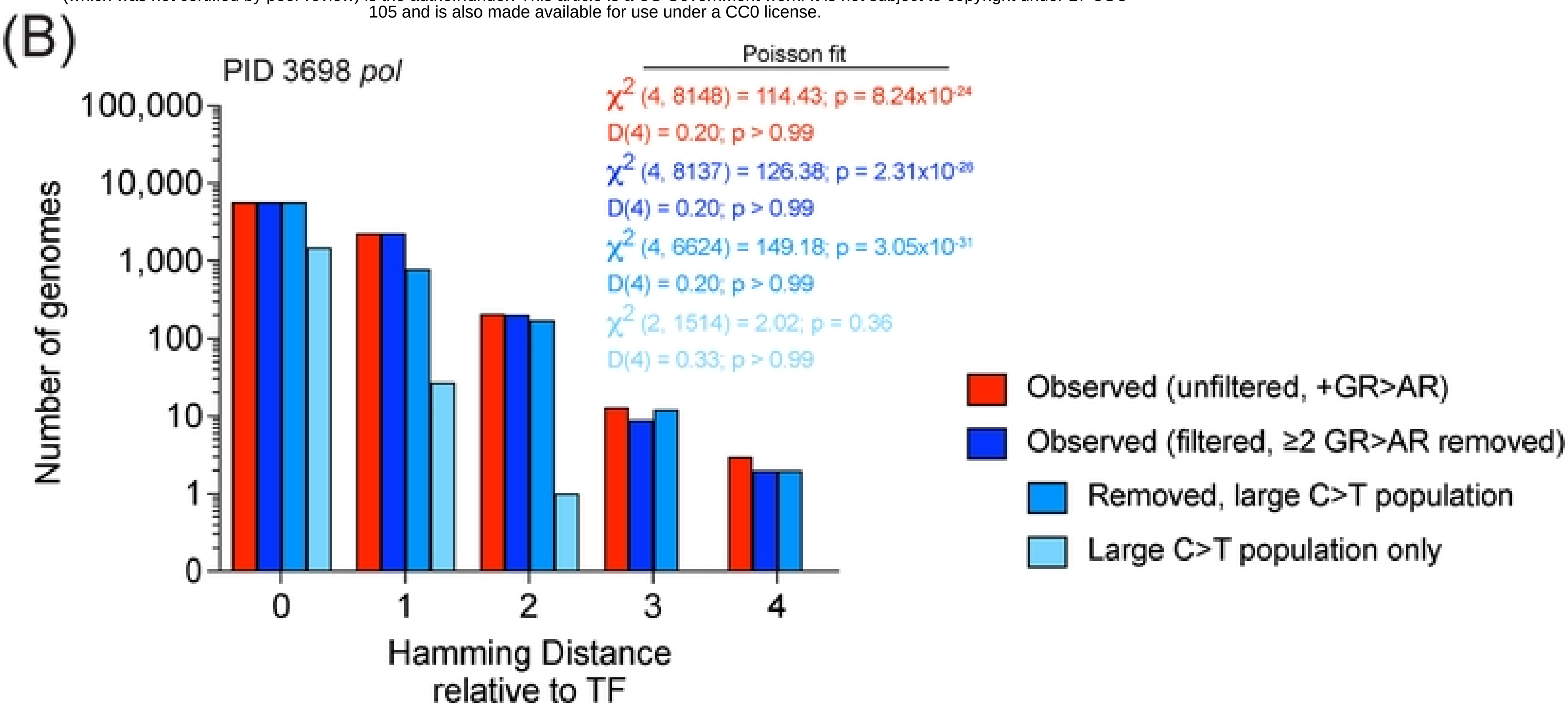

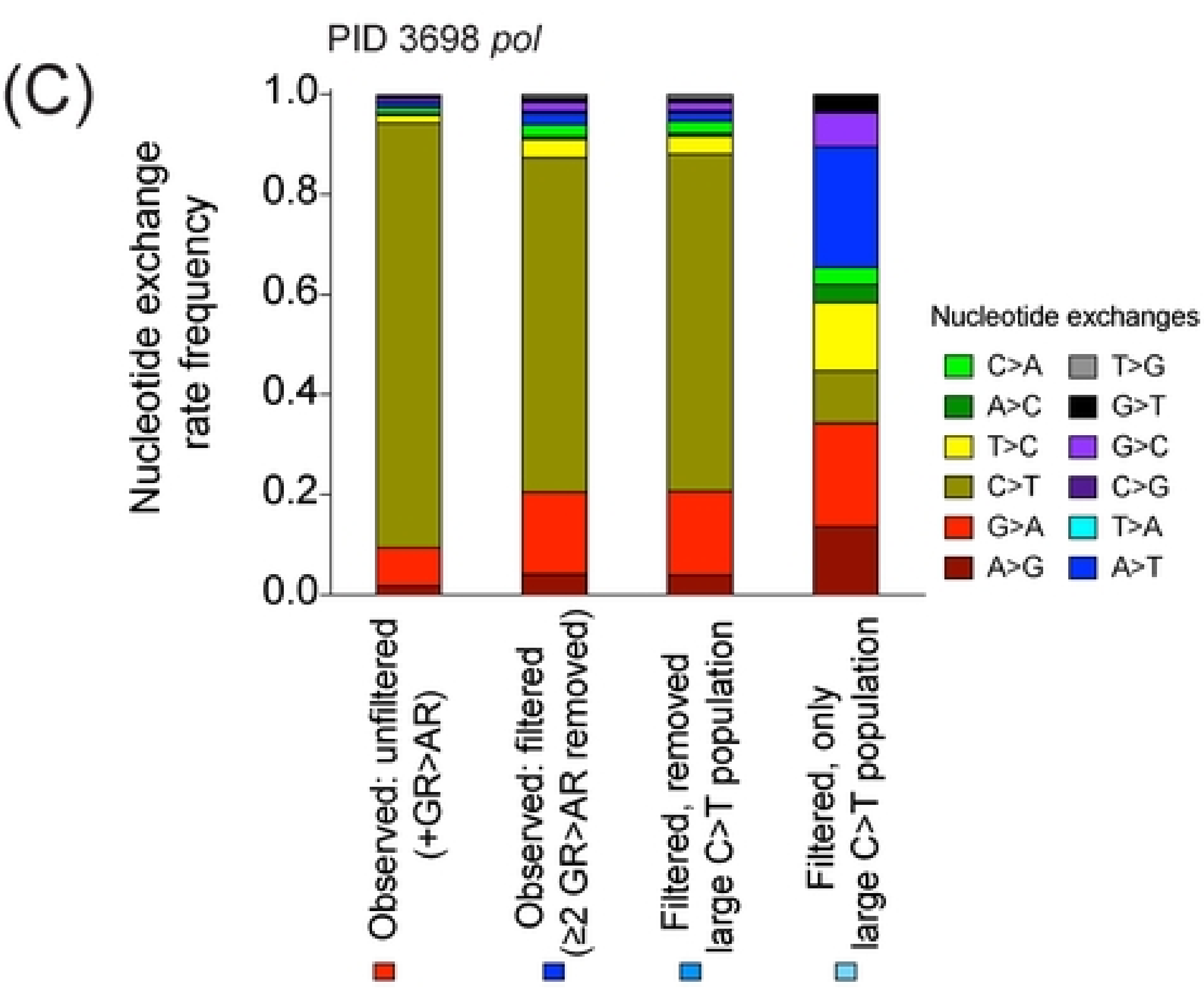

Figure S5

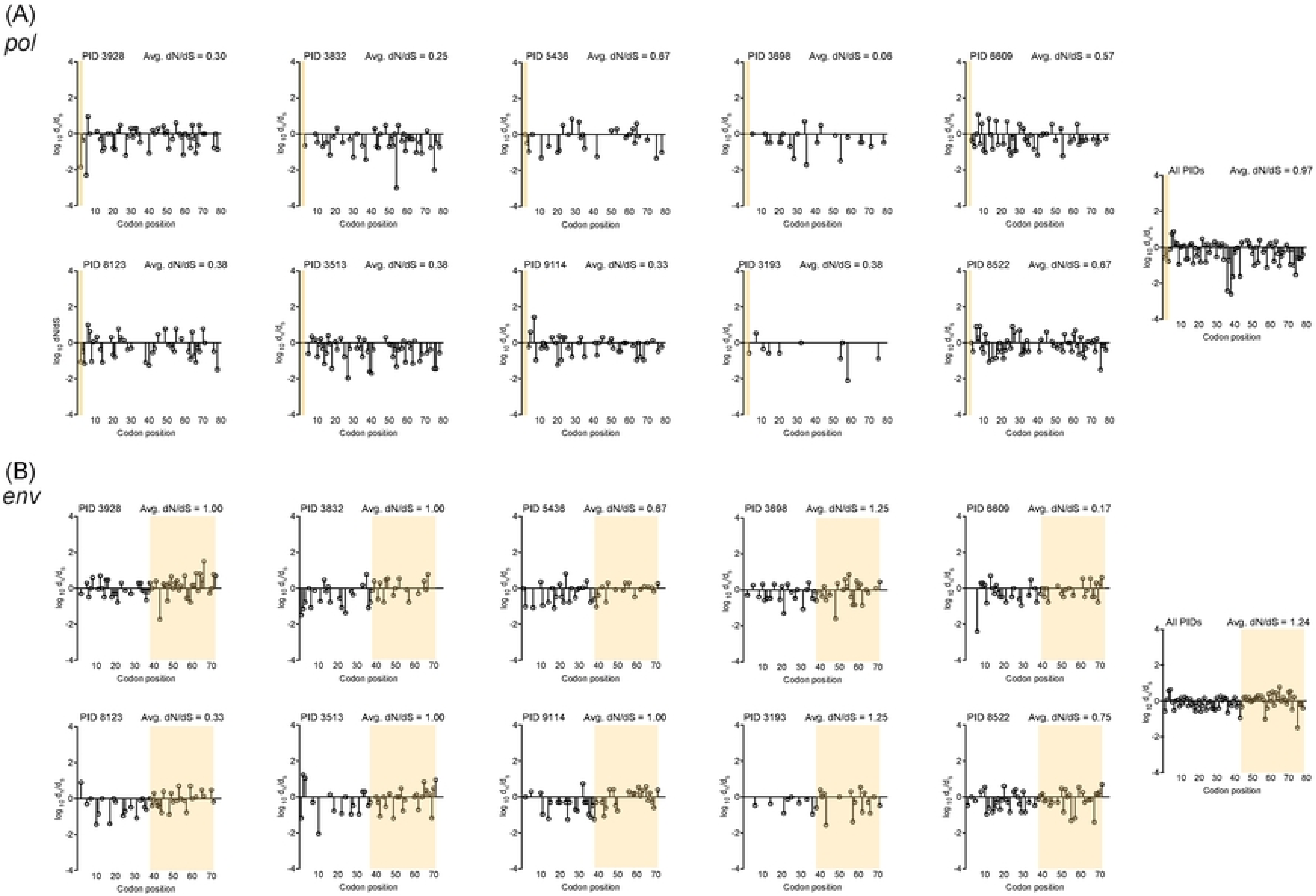

(A)
pol
PID 3928 Avg. dN/dS = 0.30
PID 3832 Avg. dN/dS = 0.25
PID 5436 Avg. dN/dS = 0.67
PID 3898 Avg. dN/dS = 0.06
PID 6609 Avg. dN/dS = 0.57
PID 8123 Avg. dN/dS = 0.38
PID 3513 Avg. dN/dS = 0.38
PID 9114 Avg. dN/dS = 0.33
PID 3193 Avg. dN/dS = 0.38
PID 8522 Avg. dN/dS = 0.67
All PIDs Avg. dN/dS = 0.97
(B)
env
PID 3928 Avg. dN/dS = 1.00
PID 3832 Avg. dN/dS = 1.00
PID 5436 Avg. dN/dS = 0.67
PID 3898 Avg. dN/dS = 1.25
PID 6609 Avg. dN/dS = 0.17
PID 8123 Avg. dN/dS = 0.33
PID 3513 Avg. dN/dS = 1.00
PID 9114 Avg. dN/dS = 1.00
PID 3193 Avg. dN/dS = 1.25
PID 8522 Avg. dN/dS = 0.75
All PIDs Avg. dN/dS = 1.24
Codon position

Figure S6

GR-AR filtered trees

<u>Order of trees</u>

3928 *pol*

bioRxiv preprint doi: https://doi.org/10.64898/2026.08.21.746200; this version posted August 26, 2026. The copyright holder for this preprint (which was not certified by peer review) is the author/funder. This article is a US Government work. It is not subject to copyright under 17 USC 105 and is also made available for use under a CC0 license.

3832 *pol*

5436 *pol*

3698 *pol*

6609 *pol*

8123 *pol*

3513 *pol*

9114 *pol*

3193 *pol*

8522 *pol*

3928 *env*

3832 *env*

5436 *env*

3698 *env*

6609 *env*

8123 *env*

3513 *env*

9114 *env*

3193 *env*

8522 env

bioRxiv preprint doi: https://doi.org/10.64898/2026.08.21.746200; this version posted August 26, 2026. The copyright holder for this preprint (which was not certified by peer review) is the author/funder. This article is a US Government work. It is not subject to copyright under 17 USC 105 and is also made available for use under a CC0 license.

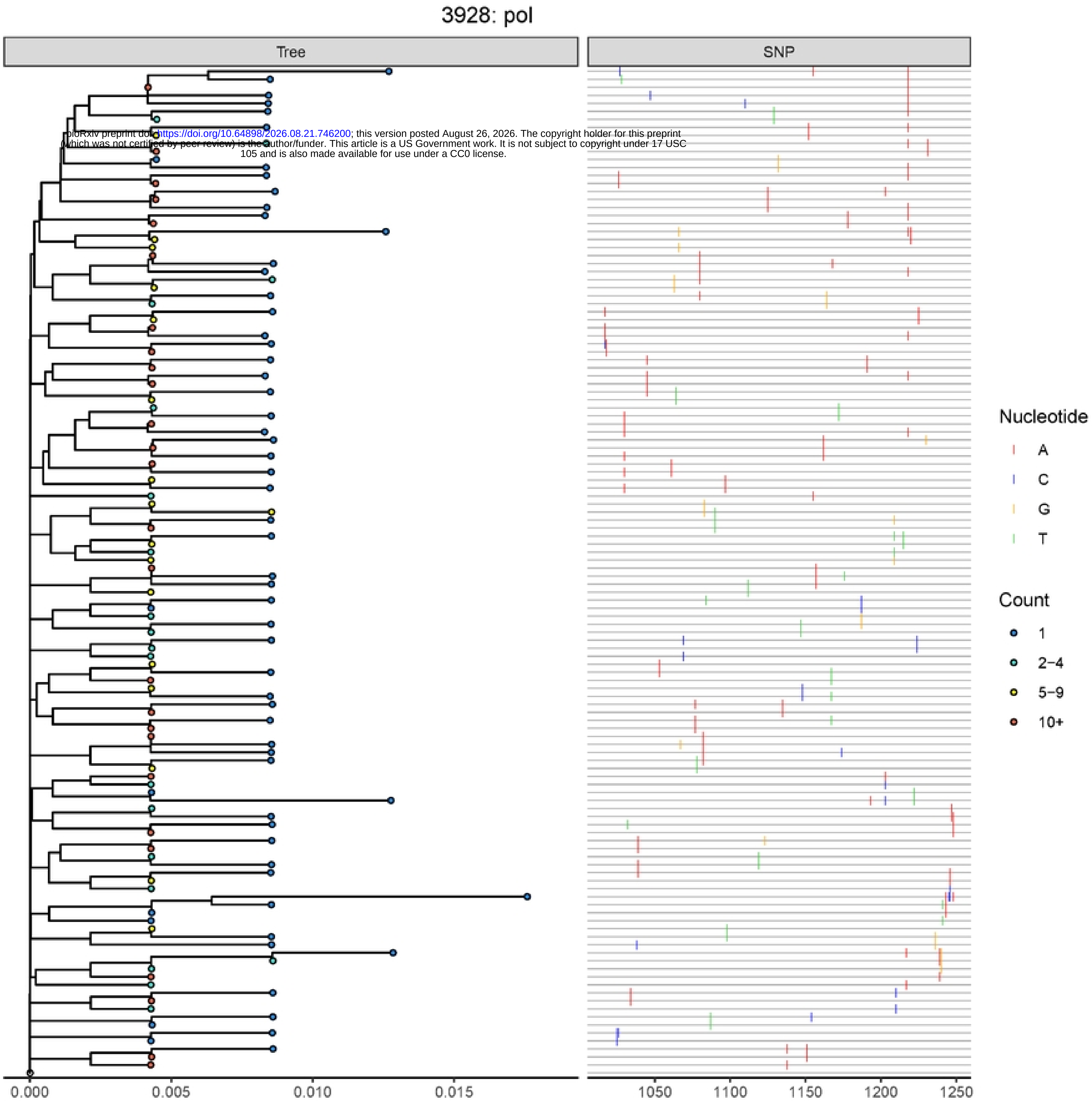

bioRxiv preprint doi: https://doi.org/10.64898/2026.08.21.746200; this version posted August 26, 2026. The copyright holder for this preprint (which was not certified by peer review) is the author/funder. This article is a US Government work. It is not subject to copyright under 17 USC 105 and is also made available for use under a CC0 license.

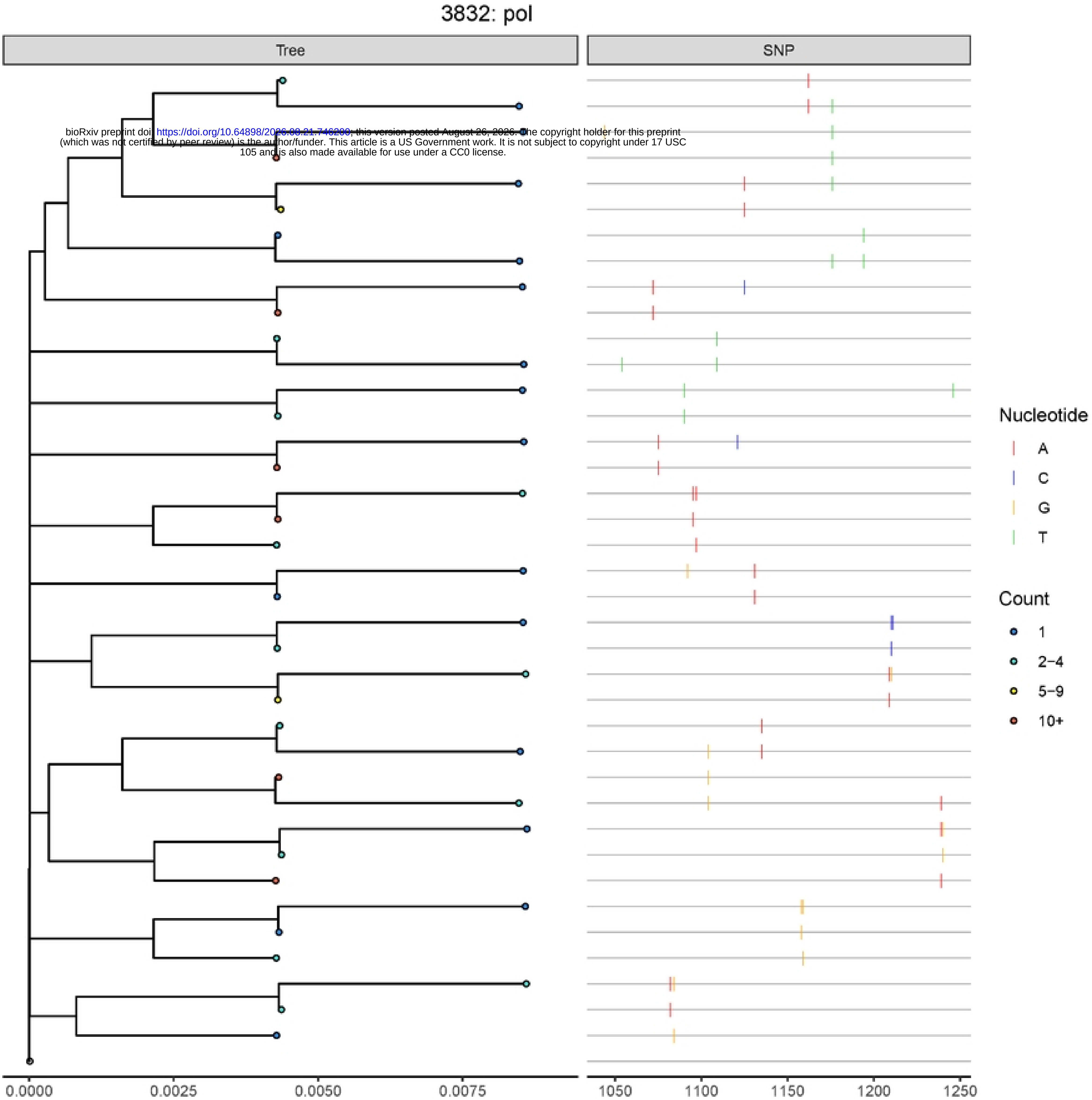

bioRxiv preprint doi: https://doi.org/10.64898/2026.08.21.746200; this version posted August 26, 2026. The copyright holder for this preprint (which was not certified by peer review) is the author/funder. This article is a US Government work. It is not subject to copyright under 17 USC 105 and is also made available for use under a CC0 license.

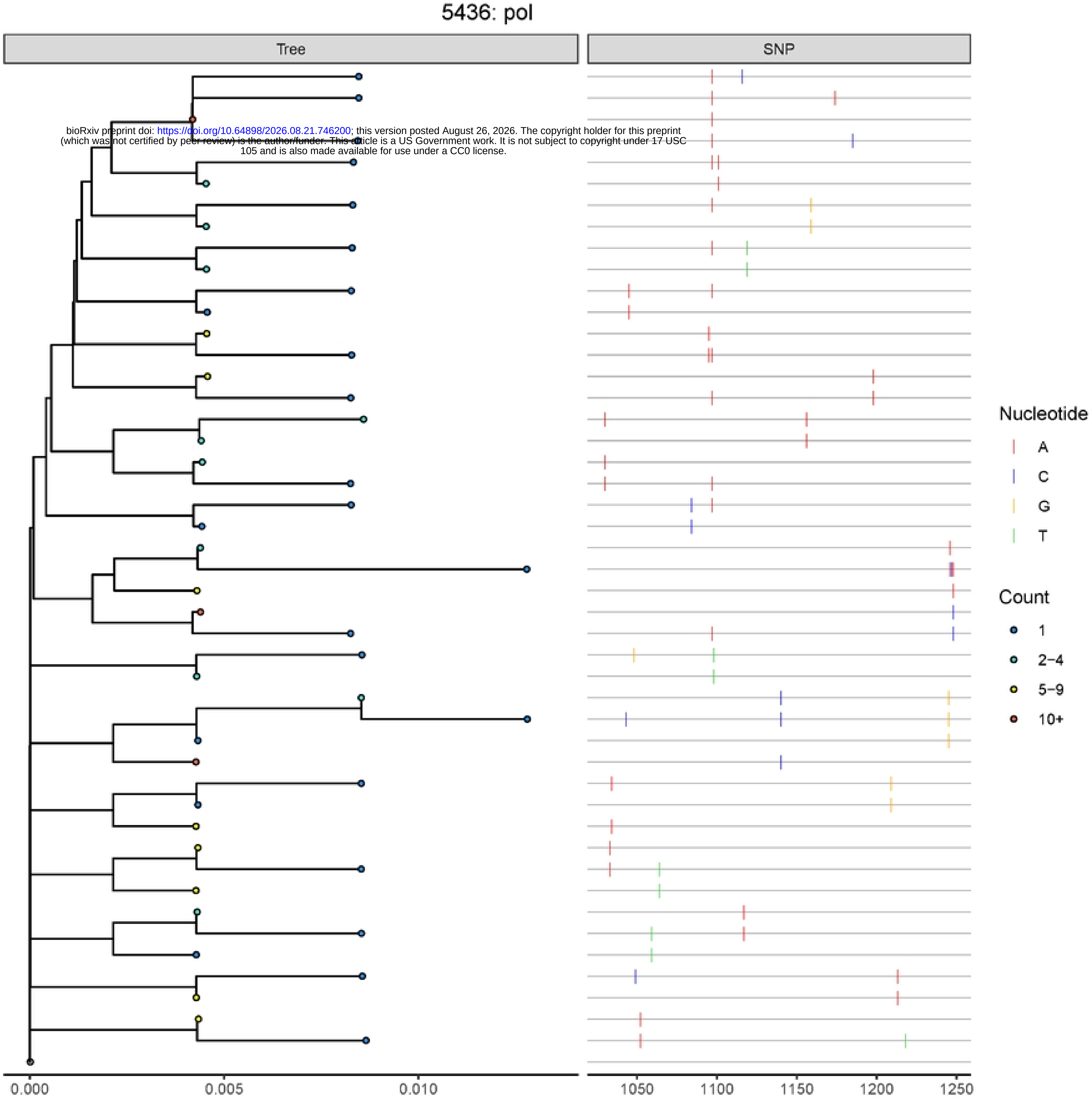

bioRxiv preprint doi: https://doi.org/10.64898/2026.08.21.746206; this version posted August 26, 2026. The copyright holder for this preprint (which was not certified by peer review) is the author/funder. This article is a US Government work. It is not subject to copyright under 17 USC 105 and is also made available for use under a CC0 license.

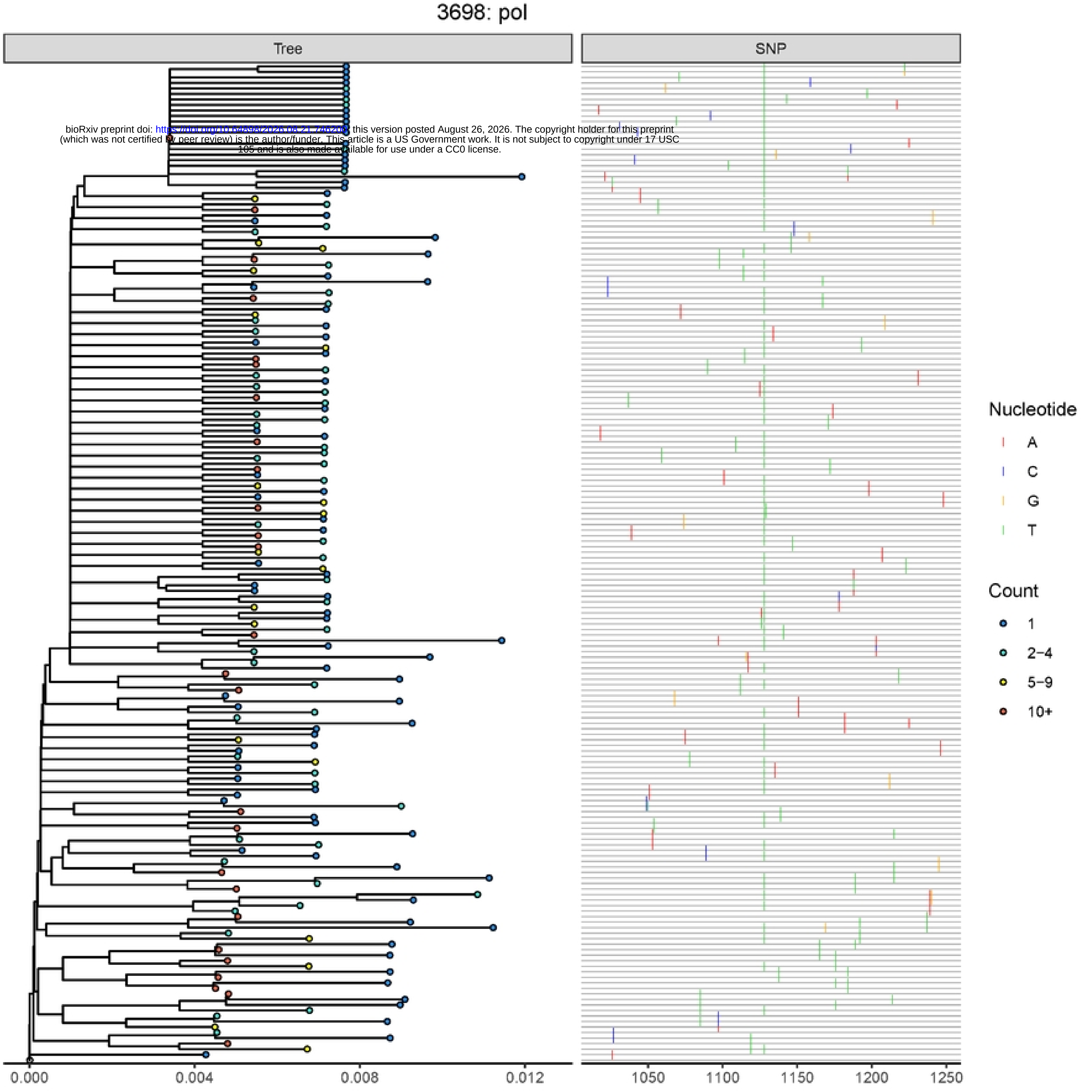

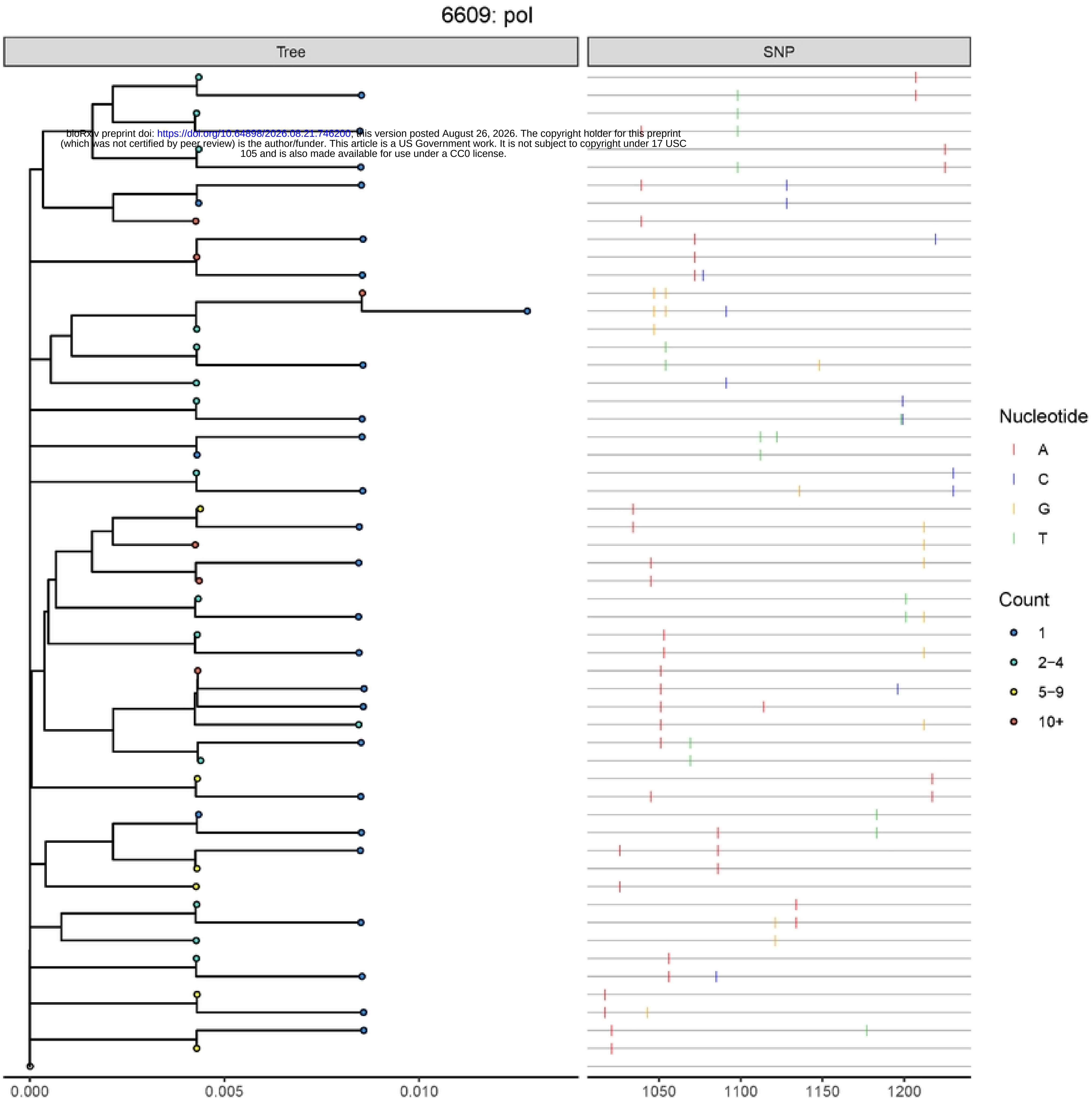

bioRxiv preprint doi: https://doi.org/10.64898/2026.08.21.746200; this version posted August 26, 2026. The copyright holder for this preprint (which was not certified by peer review) is the author/funder. This article is a US Government work. It is not subject to copyright under 17 USC 105 and is also made available for use under a CC0 license.

bioRxiv preprint doi: https://doi.org/10.64898/2026.08.21.746200; this version posted August 26, 2026. The copyright holder for this preprint (which was not certified by peer review) is the author/funder. This article is a US Government work. It is not subject to copyright under 17 USC 105 and is also made available for use under a CC0 license.

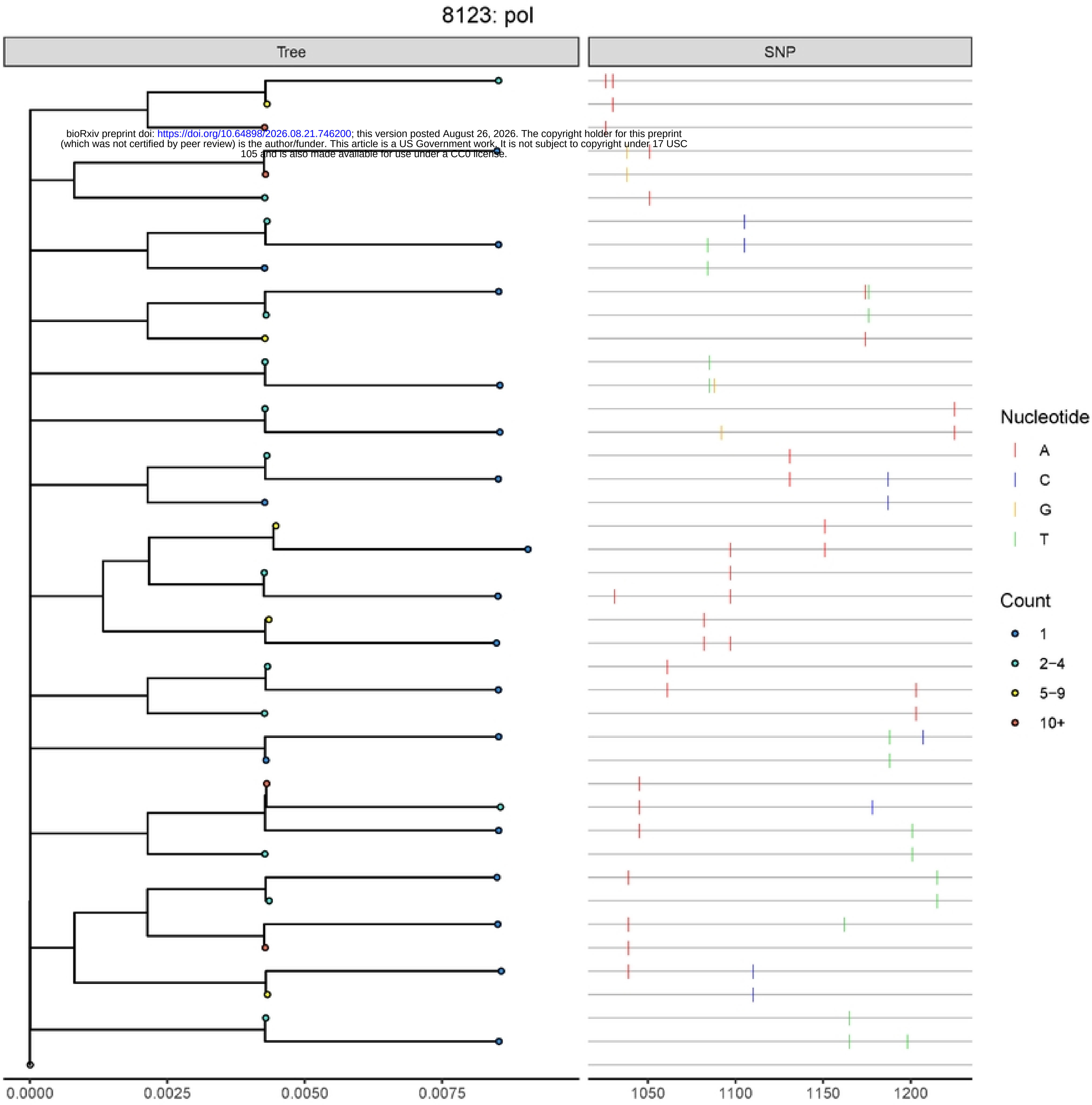

bioRxiv preprint doi: https://doi.org/10.64898/2026.08.21.746200; this version posted August 26, 2026. The copyright holder for this preprint (which was not certified by peer review) is the author/funder. This article is a US Government work. It is not subject to copyright under 17 USC 105 and is also made available for use under a CC0 license.

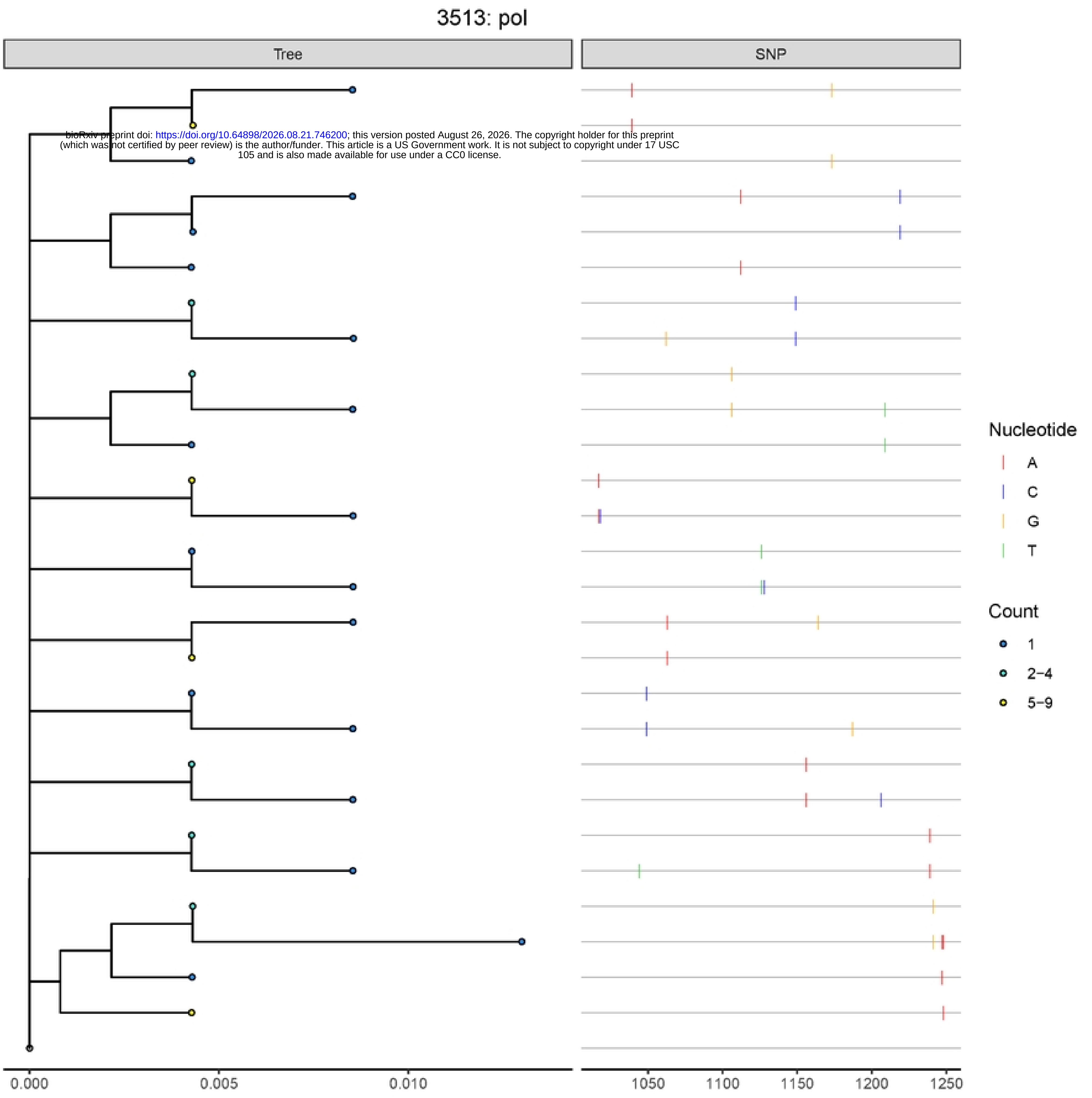

bioRxiv preprint doi: https://doi.org/10.64898/2026.08.21.746200; this version posted August 26, 2026. The copyright holder for this preprint (which was not certified by peer review) is the author/funder. This article is a US Government work. It is not subject to copyright under 17 USC 105 and is also made available for use under a CC0 license.

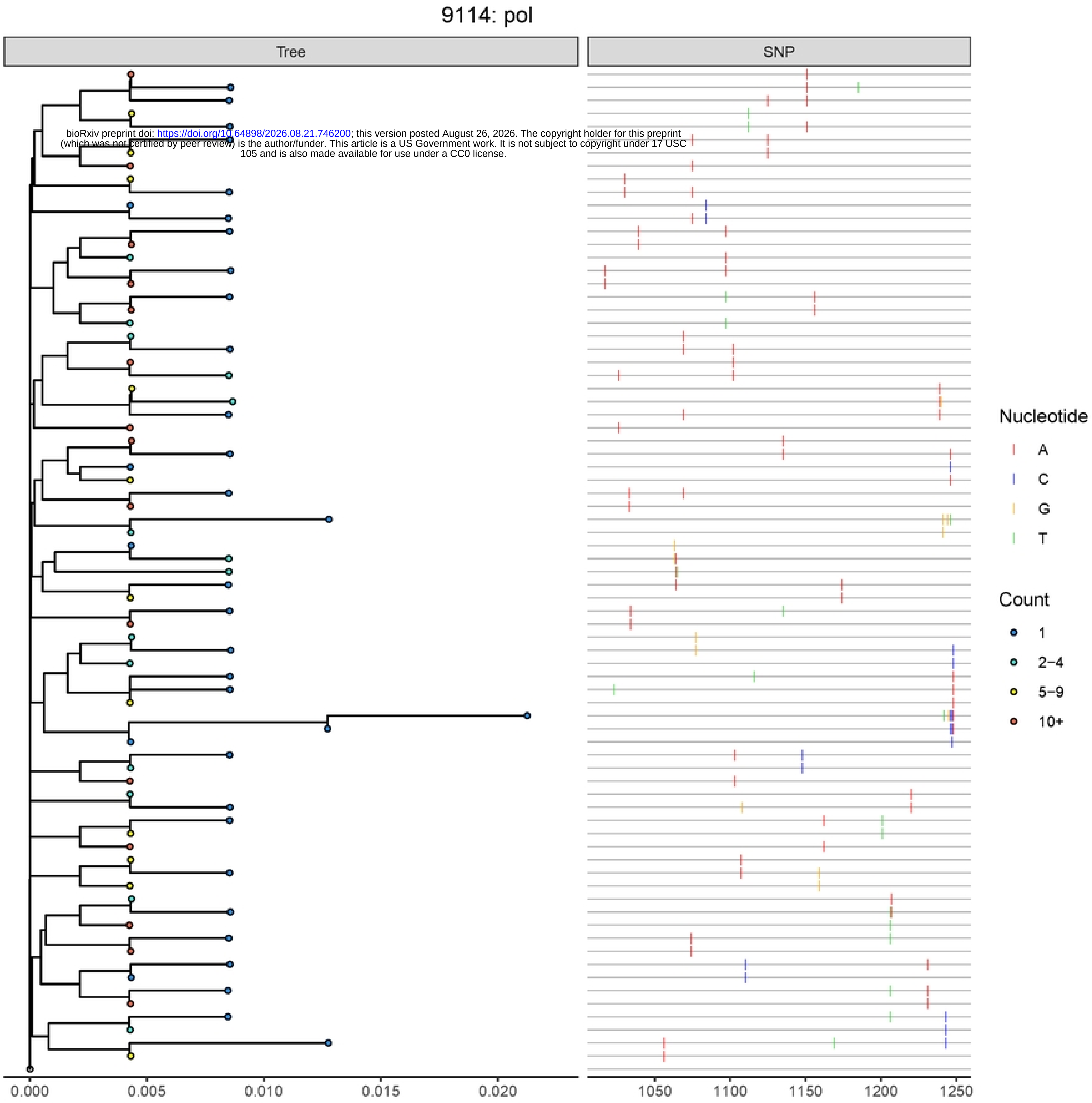

bioRxiv preprint doi: https://doi.org/10.64898/2026.08.21.746200; this version posted August 26, 2026. The copyright holder for this preprint (which was not certified by peer review) is the author/funder. This article is a US Government work. It is not subject to copyright under 17 USC 105 and is also made available for use under a CC0 license.

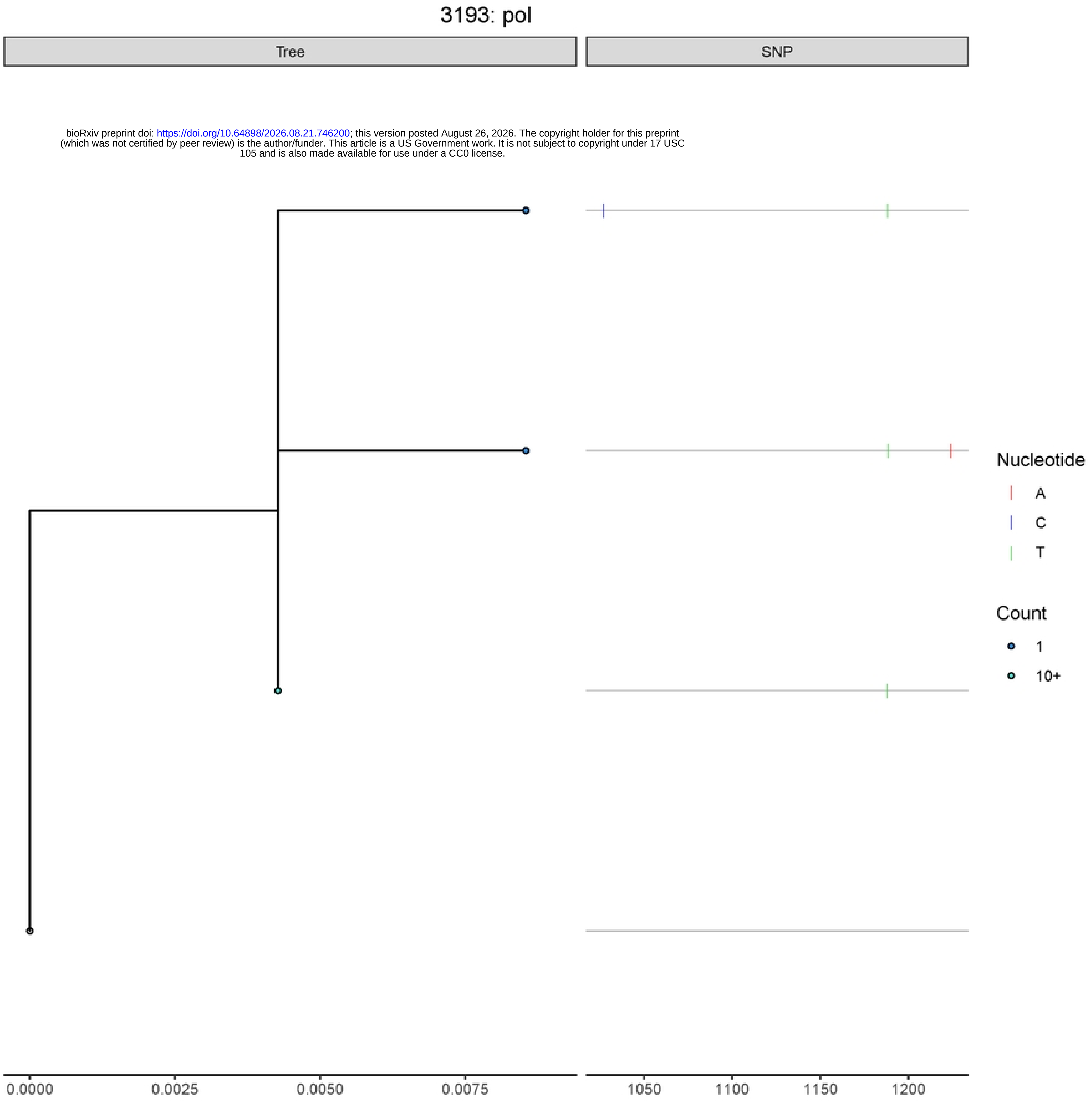

bioRxiv preprint doi: https://doi.org/10.64898/2026.08.21.746200; this version posted August 26, 2026. The copyright holder for this preprint (which was not certified by peer review) is the author/funder. This article is a US Government work. It is not subject to copyright under 17 USC 105 and is also made available for use under a CC0 license.

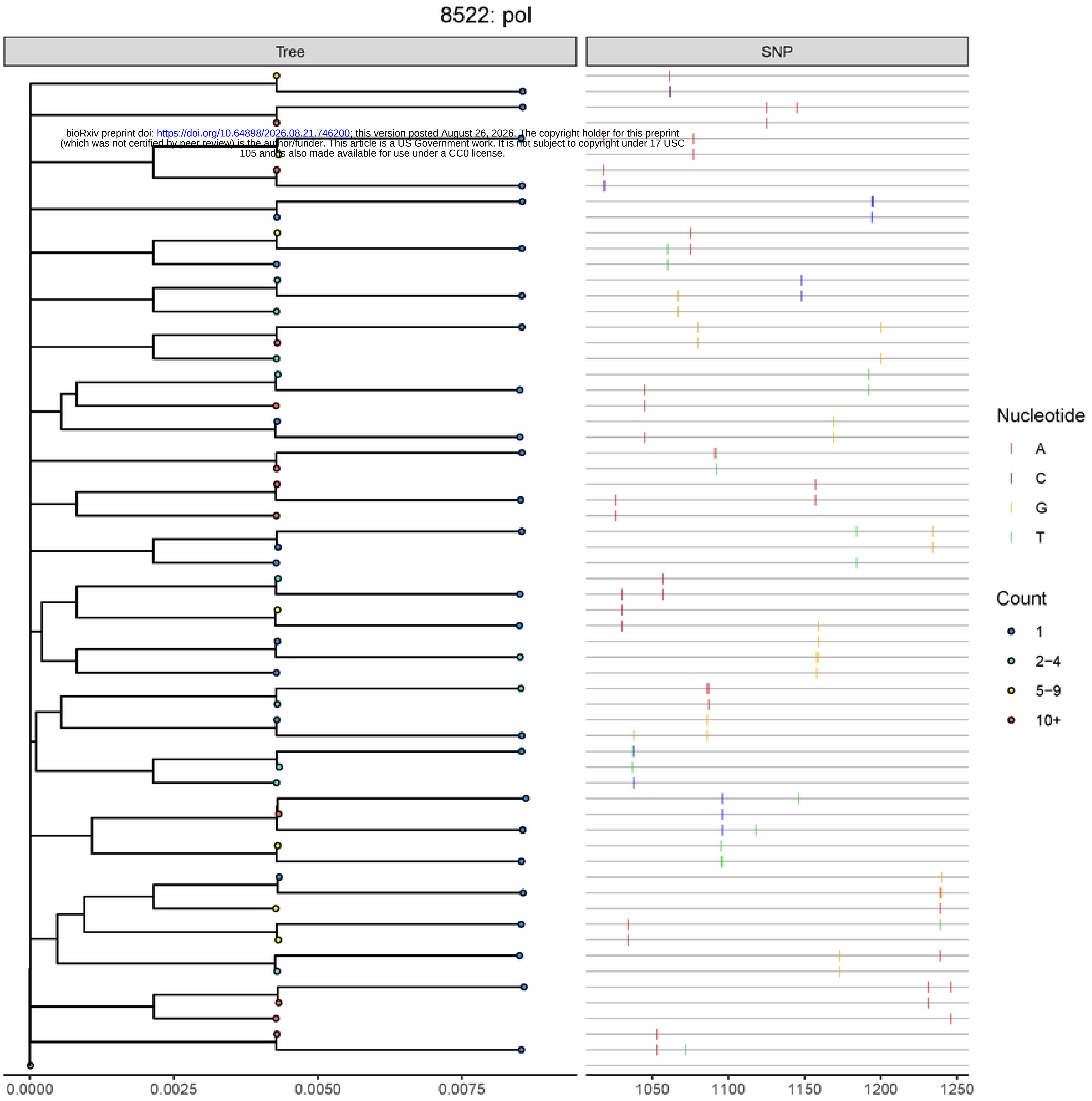

bioRxiv preprint doi: https://doi.org/10.64898/2026.08.21.746200; this version posted August 26, 2026. The copyright holder for this preprint (which was not certified by peer review) is the author/funder. This article is a US Government work. It is not subject to copyright under 17 USC 105 and is also made available for use under a CC0 license.

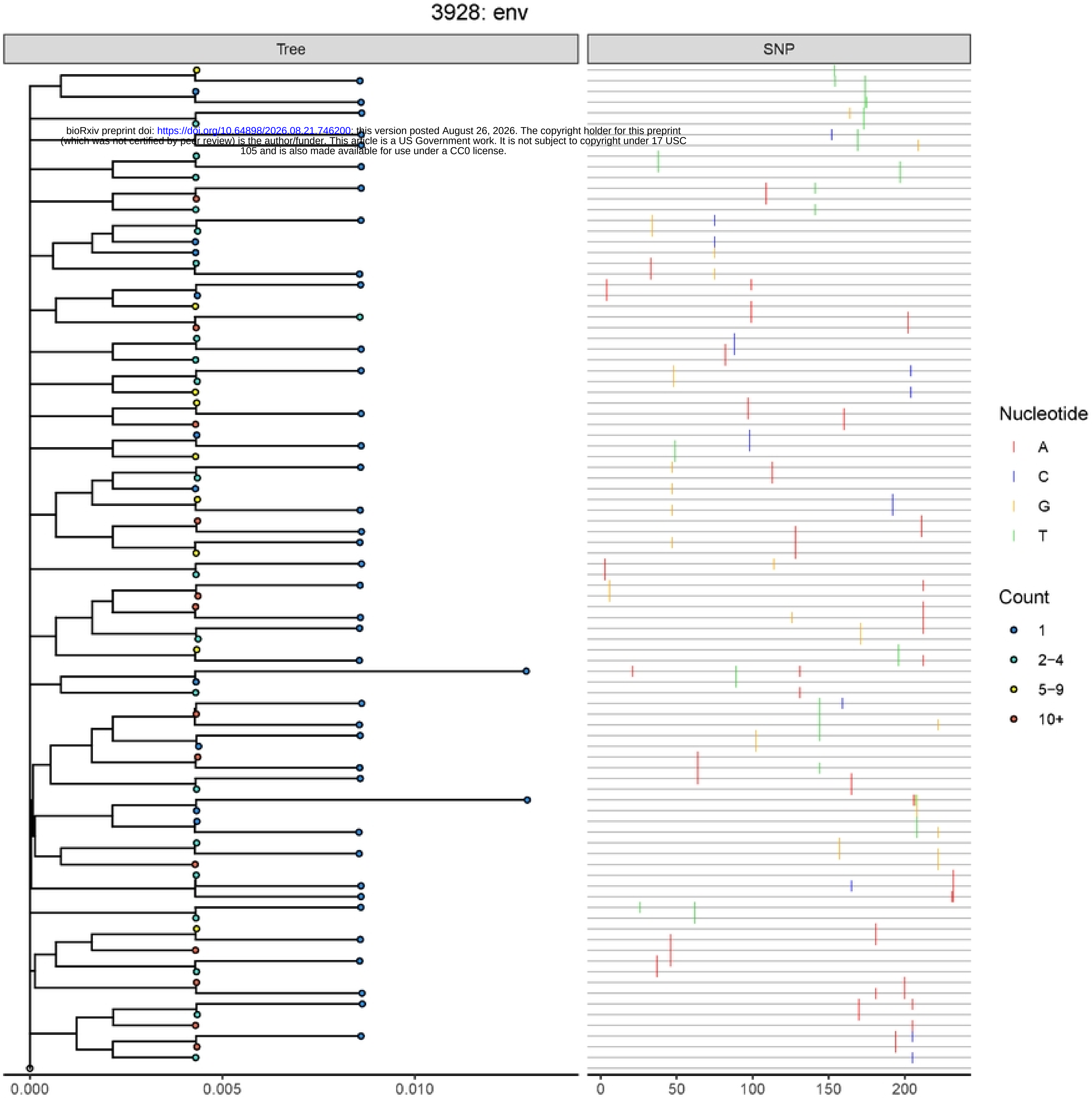

bioRxiv preprint doi: https://doi.org/10.64898/2026.08.21.746200; this version posted August 26, 2026. The copyright holder for this preprint (which was not certified by peer review) is the author/funder. This article is a US Government work. It is not subject to copyright under 17 USC 105 and is also made available for use under a CC0 license.

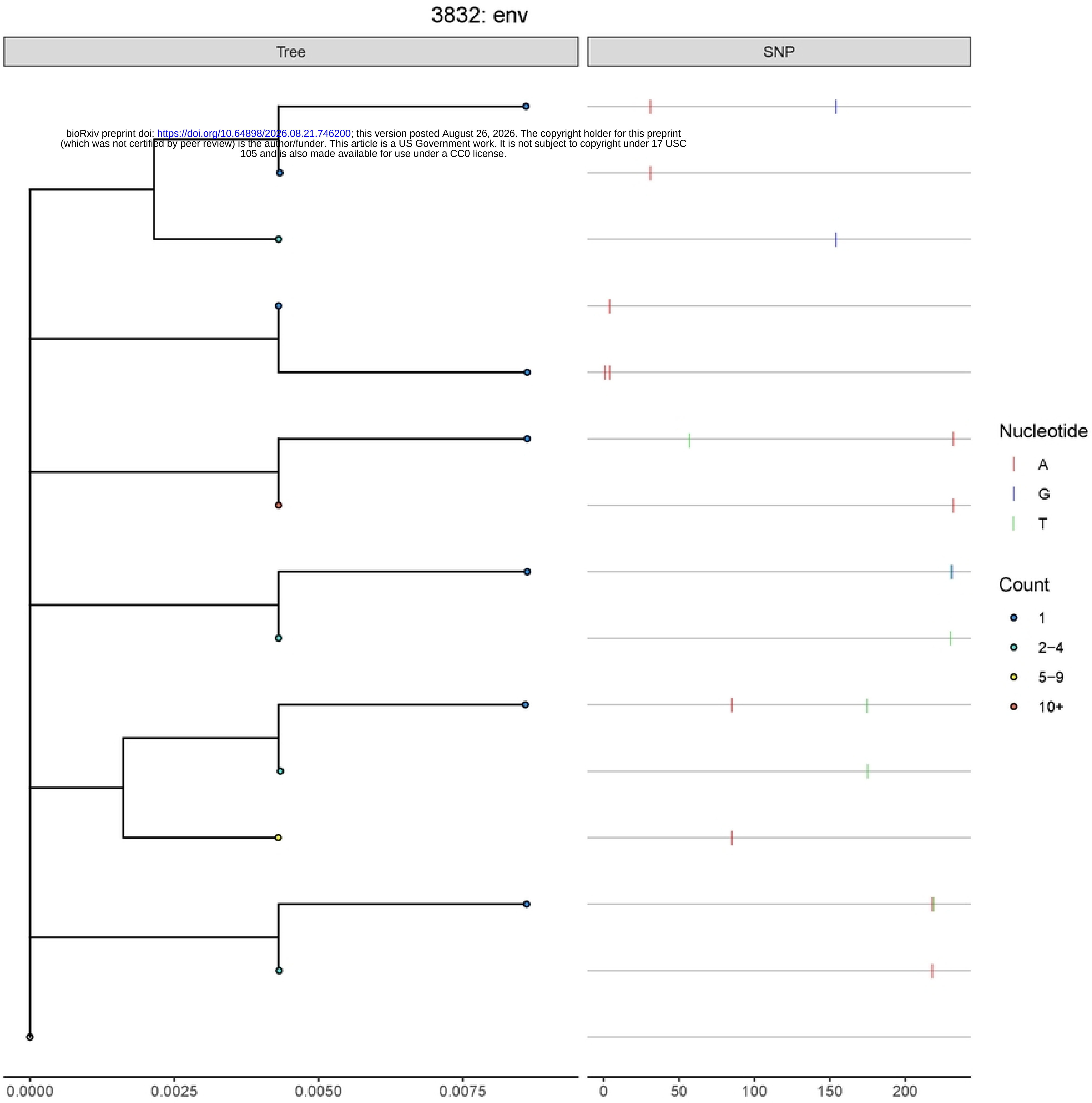

bioRxiv preprint doi: https://doi.org/10.64898/2026.08.21.746200; this version posted August 26, 2026. The copyright holder for this preprint (which was not certified by peer review) is the author/funder. This article is a US Government work. It is not subject to copyright under 17 USC 105 and is also made available for use under a CC0 license.

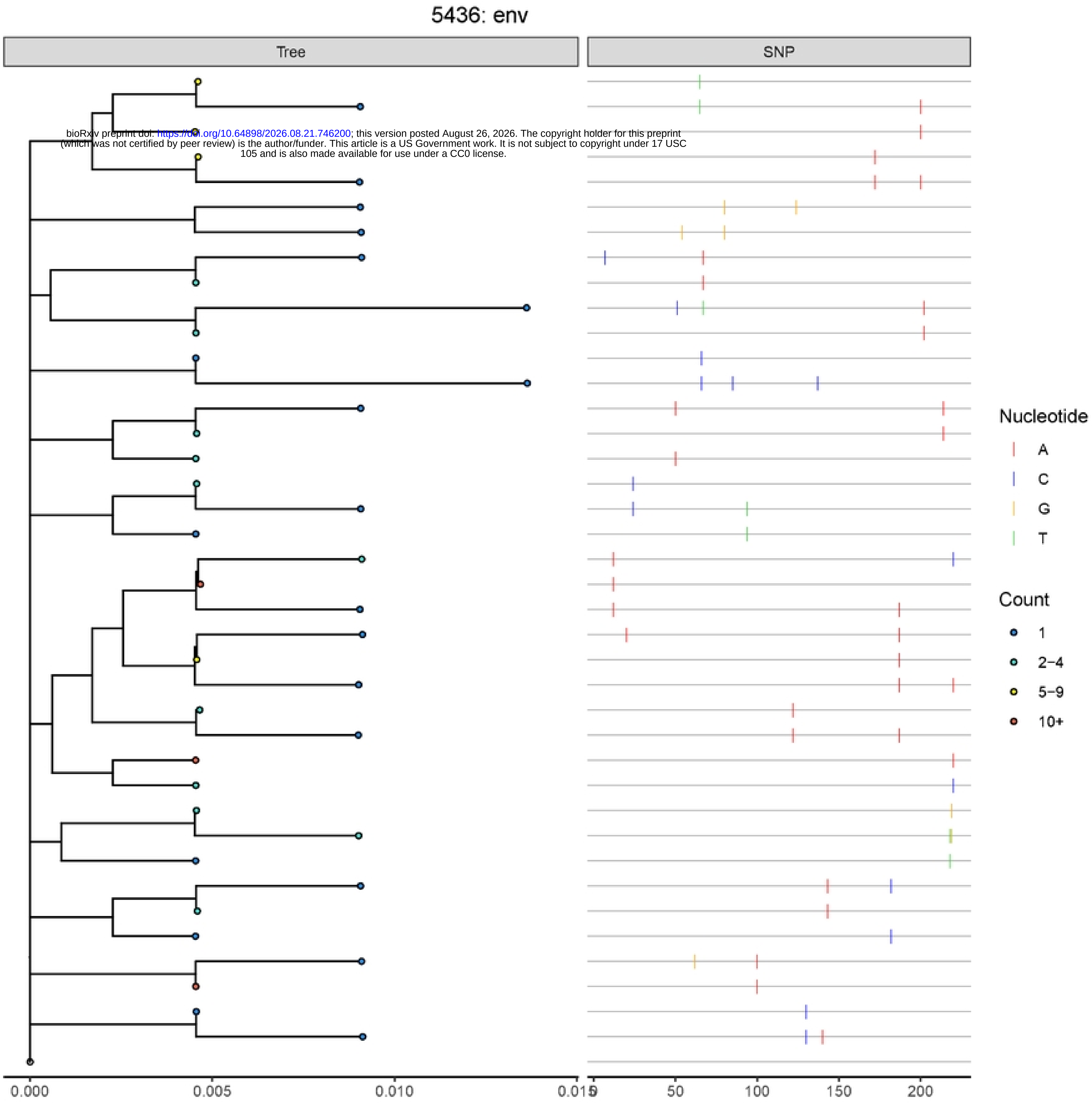

bioRxiv preprint doi: https://doi.org/10.64898/2026.08.21.746200; this version posted August 26, 2026. The copyright holder for this preprint (which was not certified by peer review) is the author/funder. This article is a US Government work. It is not subject to copyright under 17 USC 105 and is also made available for use under a CC0 license.

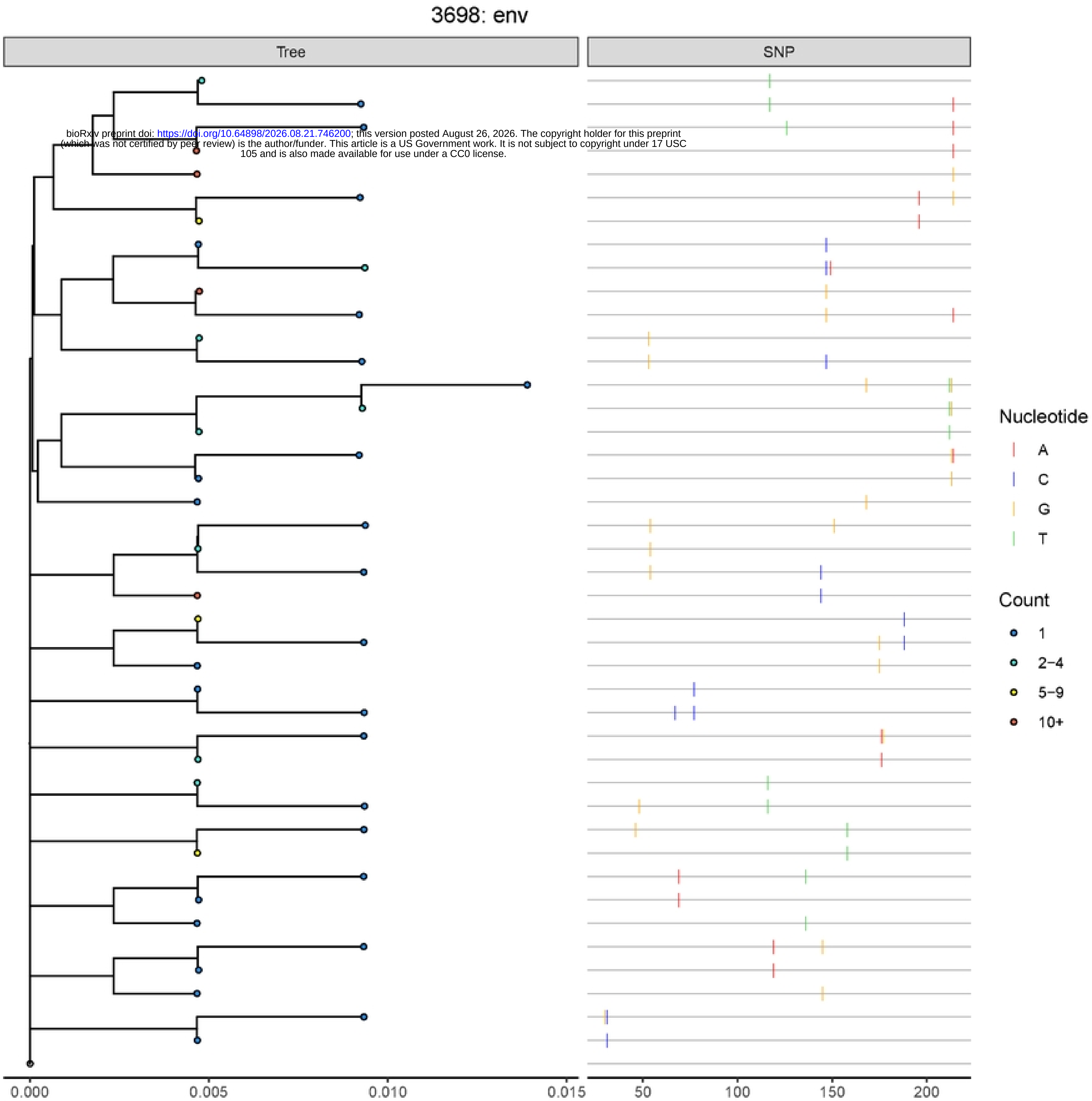

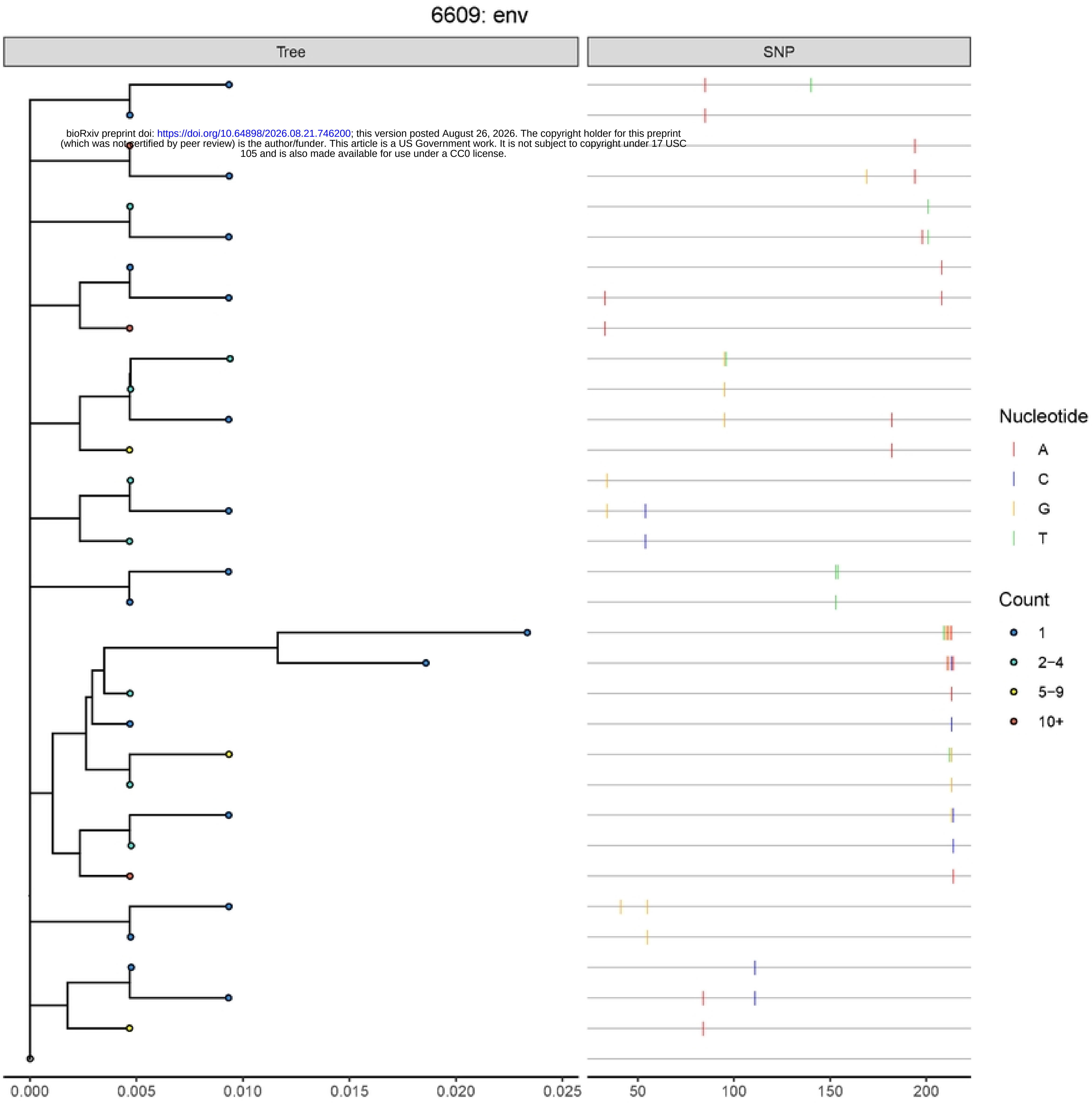

bioRxiv preprint doi: https://doi.org/10.64898/2026.08.21.746200; this version posted August 26, 2026. The copyright holder for this preprint (which was not certified by peer review) is the author/funder. This article is a US Government work. It is not subject to copyright under 17 USC 105 and is also made available for use under a CC0 license.

bioRxiv preprint doi: https://doi.org/10.64898/2026.08.21.746200; this version posted August 26, 2026. The copyright holder for this preprint (which was not certified by peer review) is the author/funder. This article is a US Government work. It is not subject to copyright under 17 USC 105 and is also made available for use under a CC0 license.

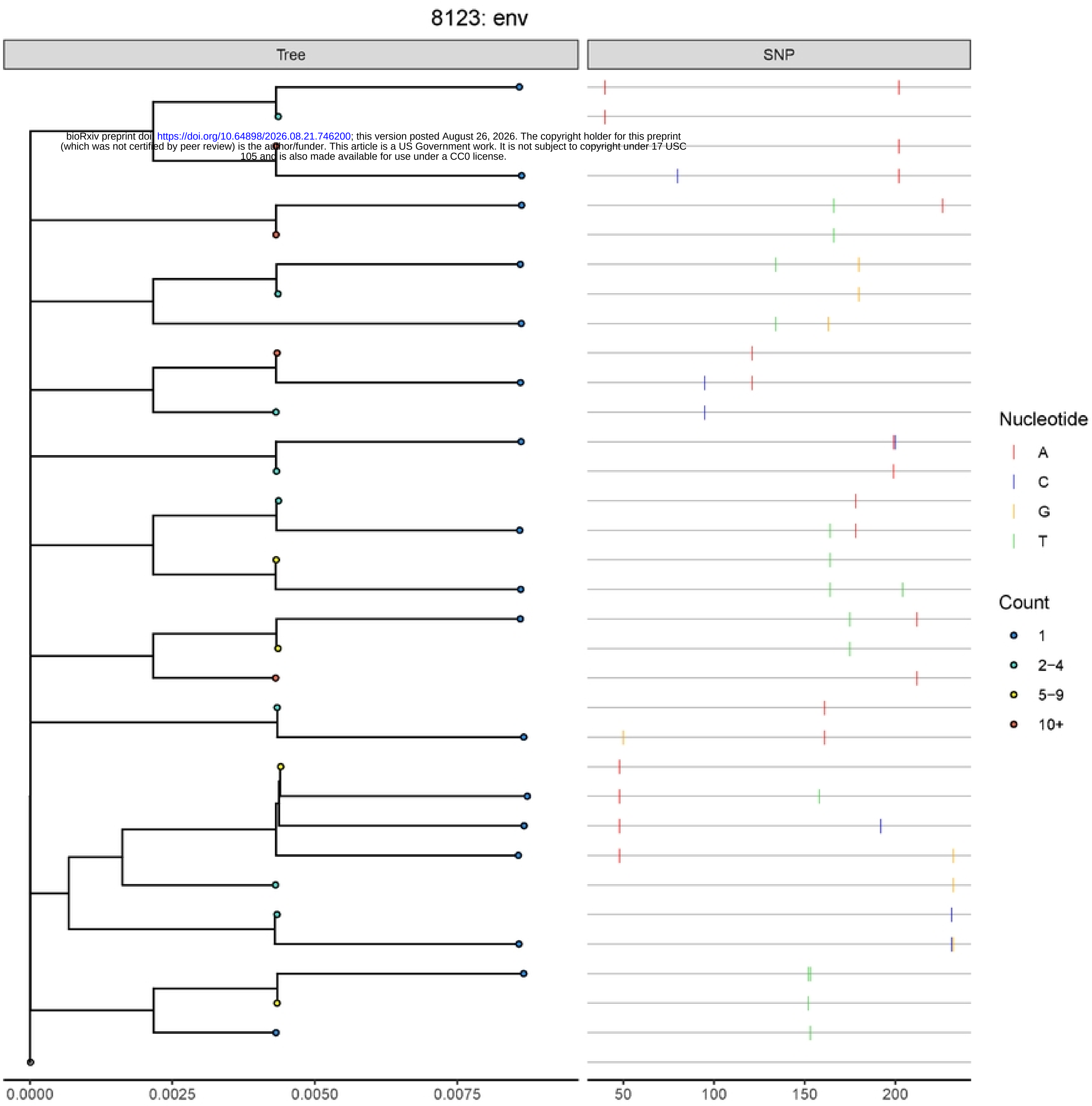

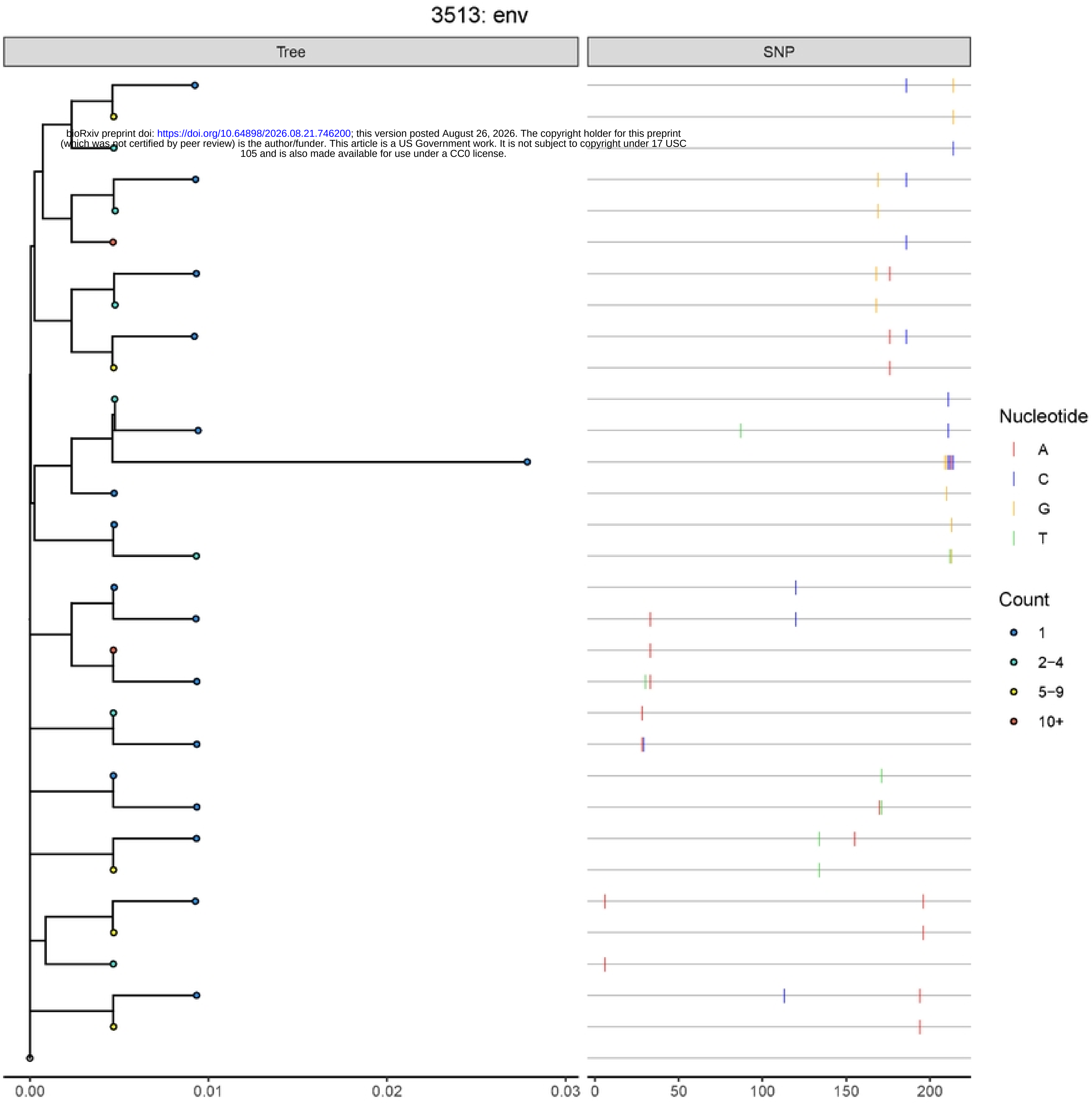

bioRxiv preprint doi: https://doi.org/10.64898/2026.08.21.746200; this version posted August 26, 2026. The copyright holder for this preprint (which was not certified by peer review) is the author/funder. This article is a US Government work. It is not subject to copyright under 17 USC 105 and is also made available for use under a CC0 license.

bioRxiv preprint doi: https://doi.org/10.64898/2026.08.21.746200; this version posted August 26, 2026. The copyright holder for this preprint (which was not certified by peer review) is the author/funder. This article is a US Government work. It is not subject to copyright under 17 USC 105 and is also made available for use under a CC0 license.

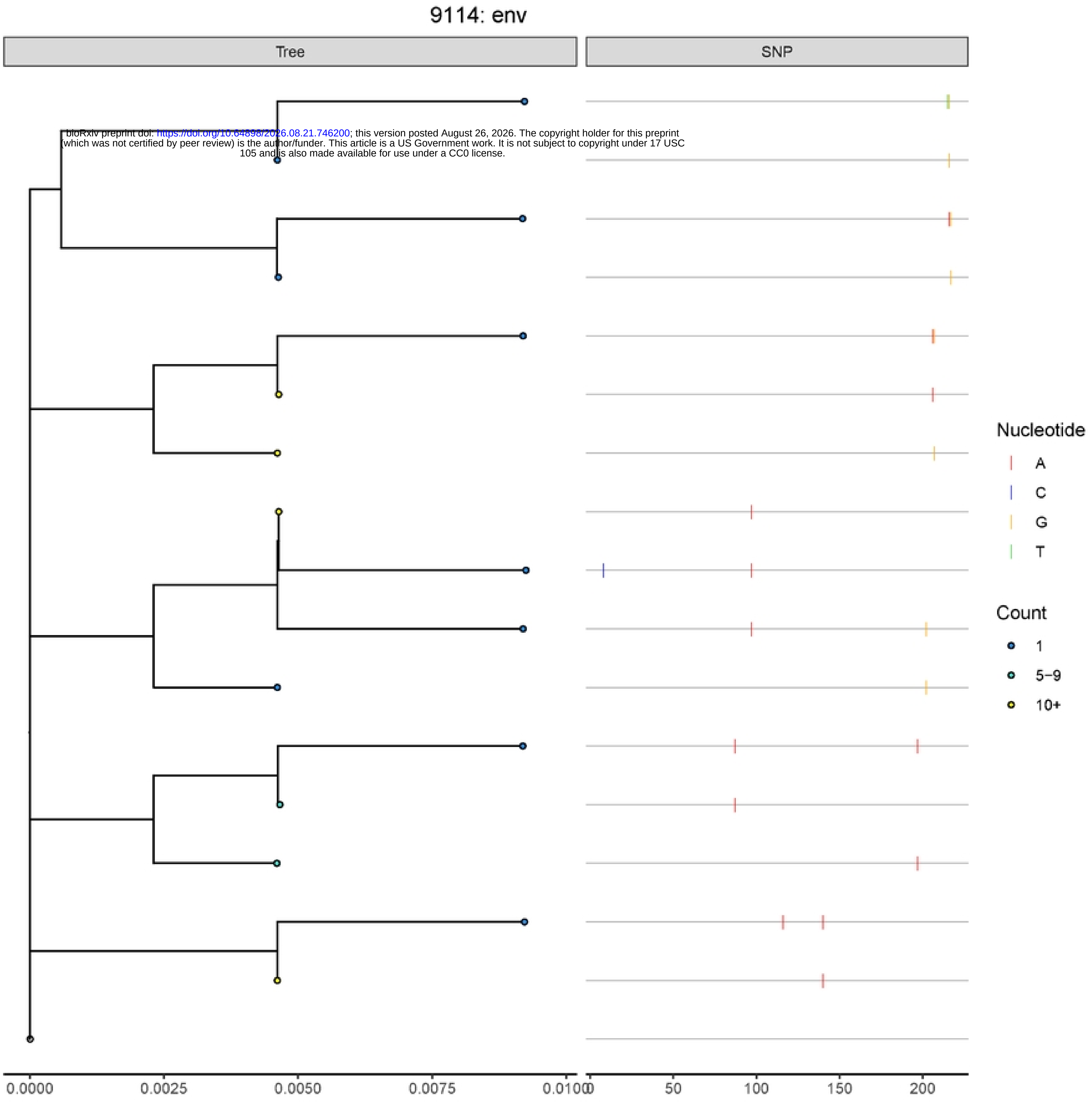

bioRxiv preprint doi: https://doi.org/10.64898/2026.08.21.746200; this version posted August 26, 2026. The copyright holder for this preprint (which was not certified by peer review) is the author/funder. This article is a US Government work. It is not subject to copyright under 17 USC 105 and is also made available for use under a CC0 license.

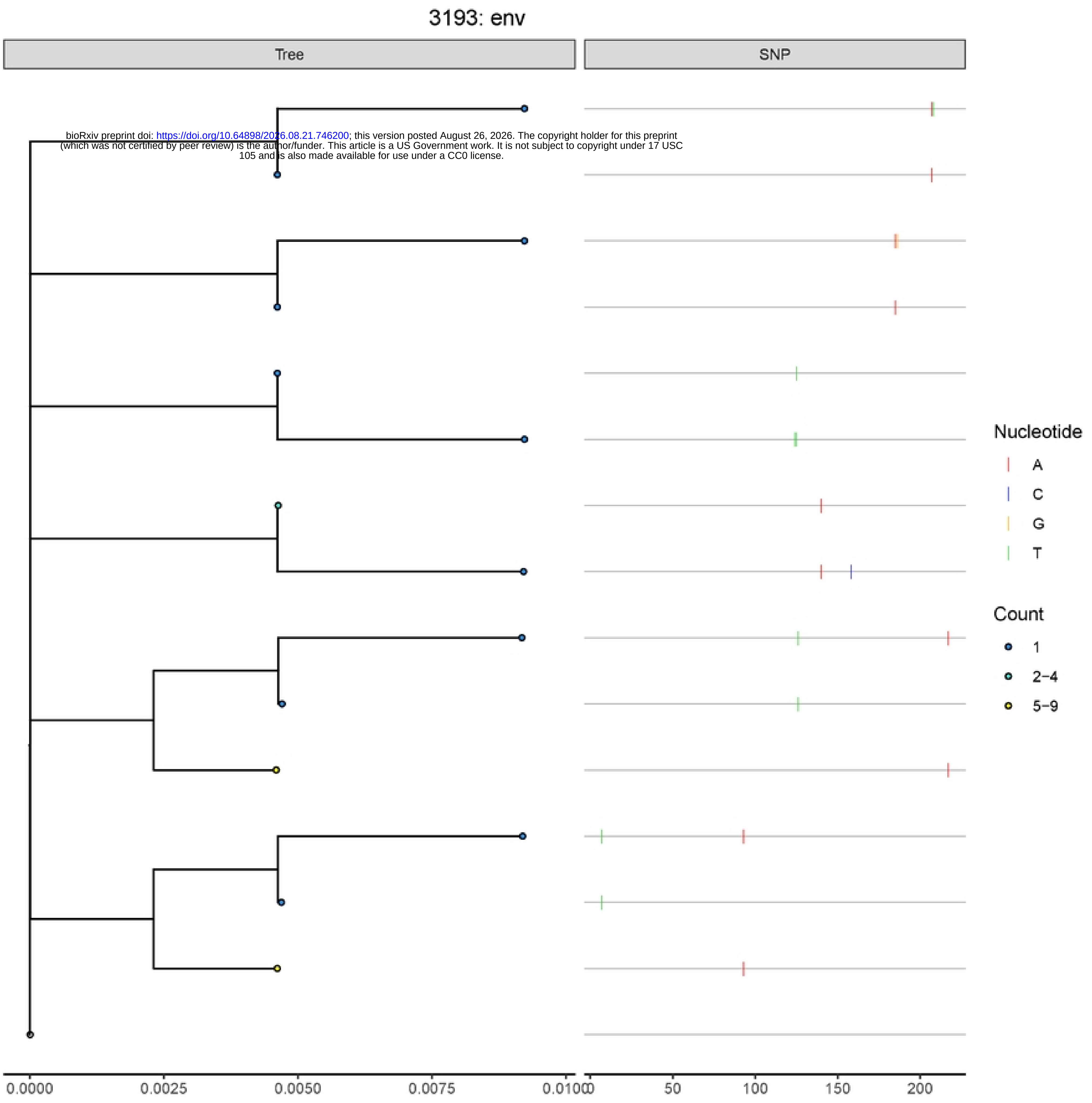

bioRxiv preprint doi: https://doi.org/10.64898/2026.08.21.746200; this version posted August 26, 2026. The copyright holder for this preprint (which was not certified by peer review) is the author/funder. This article is a US Government work. It is not subject to copyright under 17 USC 105 and is also made available for use under a CC0 license.

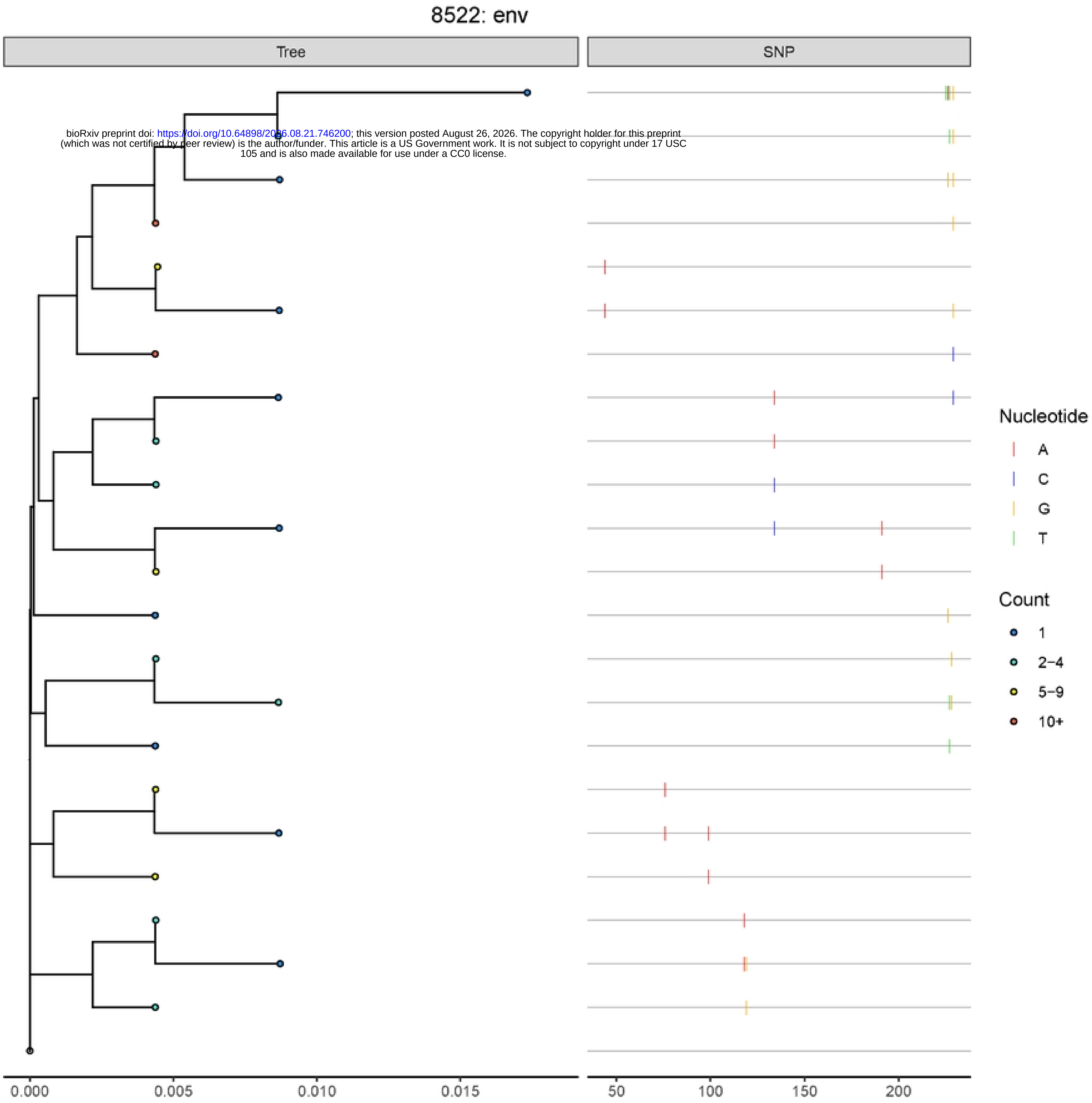

Figure S7 unfiltered GR-AR trees

Order of trees

3928 *pol*

bioRxiv preprint doi: https://doi.org/10.64898/2026.08.21.746200; this version posted August 26, 2026. The copyright holder for this preprint (which was not certified by peer review) is the author/funder. This article is a US Government work. It is not subject to copyright under 17 USC 105 and is also made available for use under a CC0 license.

3832 *pol*

5436 *pol*

3698 *pol*

6609 *pol*

8123 *pol*

3513 *pol*

9114 *pol*

3193 *pol*

8522 *pol*

3928 *env*

3832 *env*

5436 *env*

3698 *env*

6609 *env*

8123 *env*

3513 *env*

9114 *env*

3193 *env*

8522 env

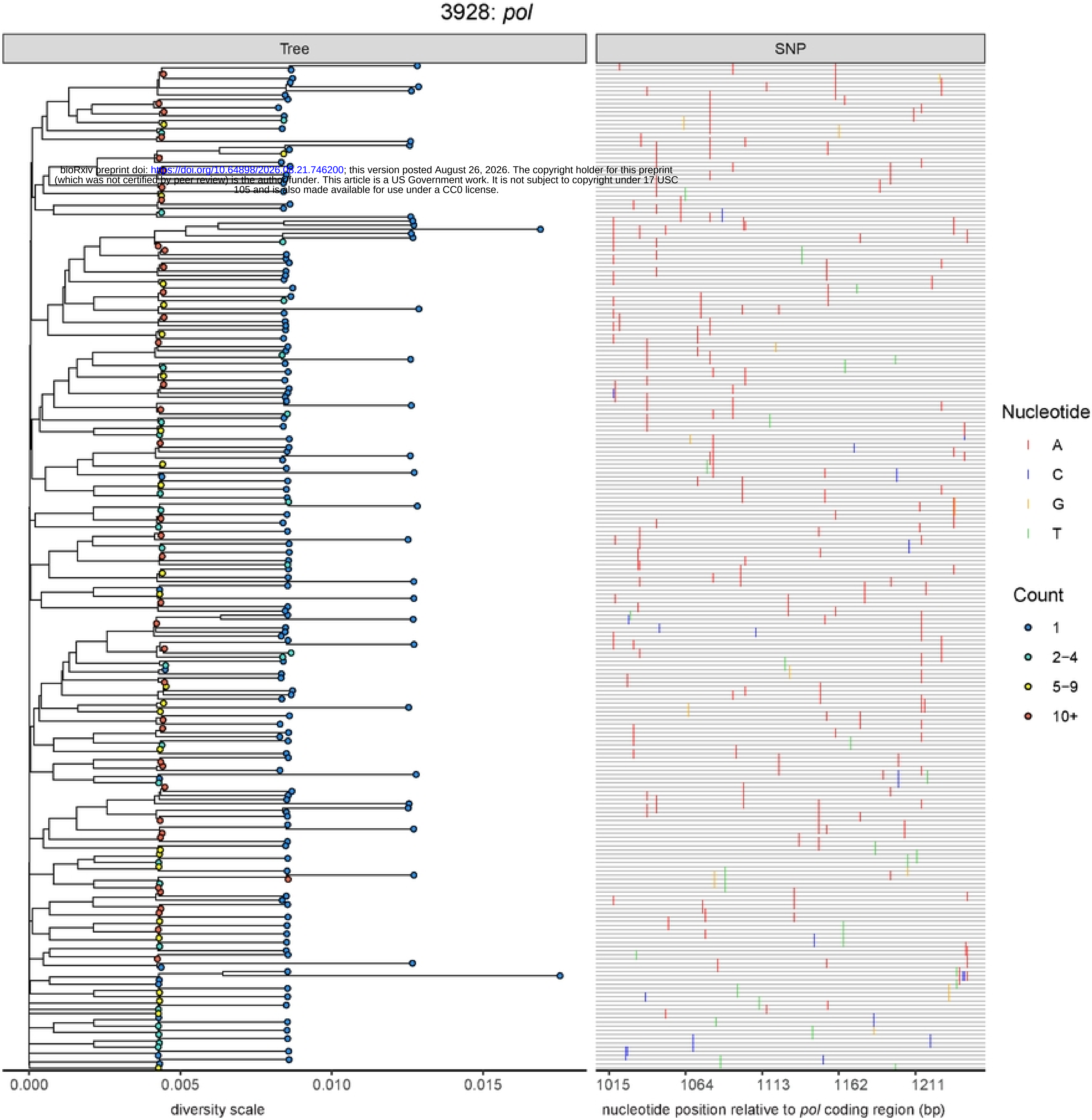

bioRxiv preprint doi: https://doi.org/10.64898/2026.08.21.746200; this version posted August 26, 2026. The copyright holder for this preprint (which was not certified by peer review) is the author/funder. This article is a US Government work. It is not subject to copyright under 17 USC 105 and is also made available for use under a CC0 license.

3832: *pol*

bioRxiv preprint doi: https://doi.org/10.64898/2026.08.21.746200; this version posted August 26, 2026. The copyright holder for this preprint (which was not certified by peer review) is the author/funder. This article is a US Government work. It is not subject to copyright under 17 USC 105 and is also made available for use under a CC0 license.

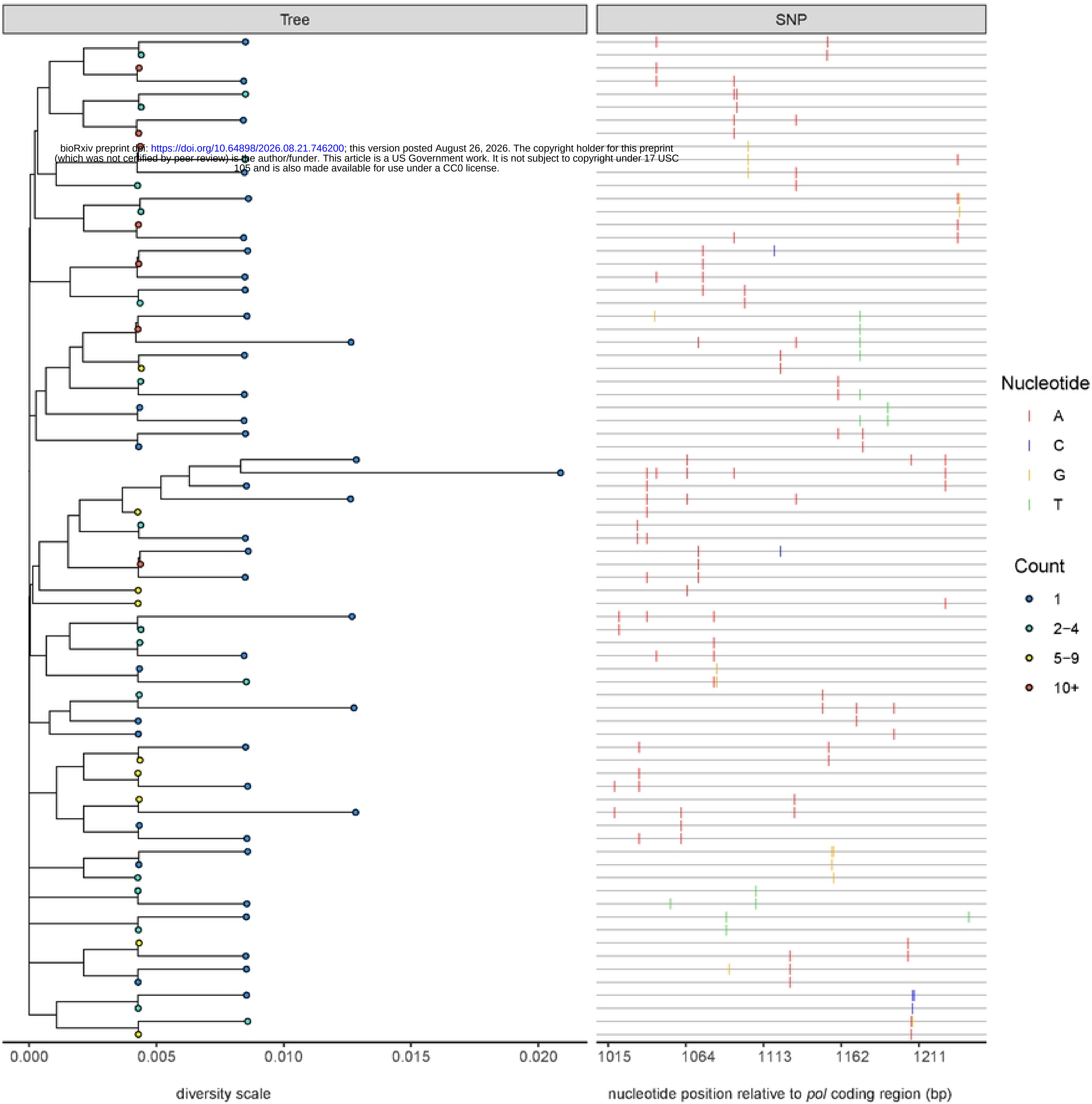

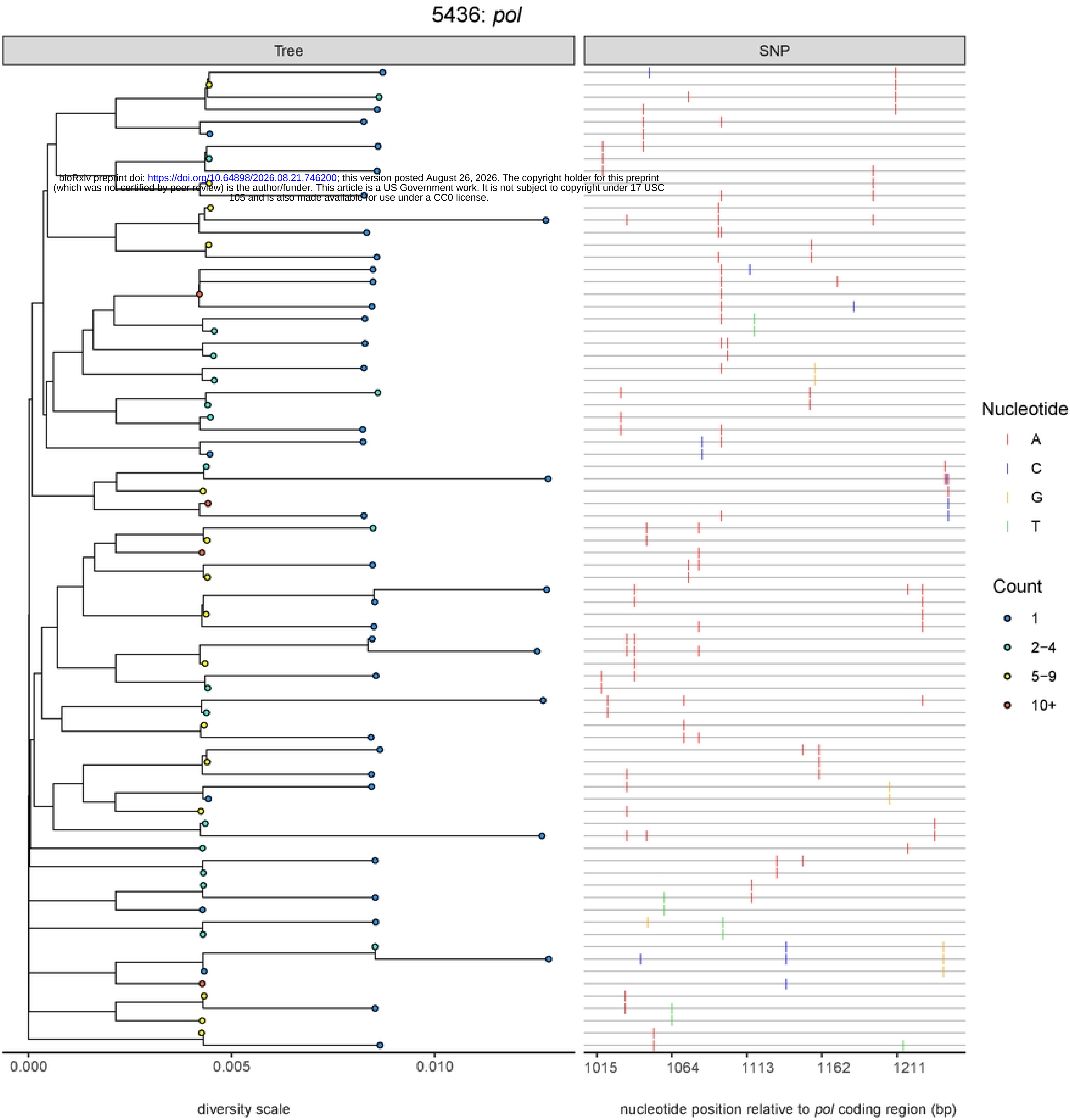

bioRxiv preprint doi: https://doi.org/10.64898/2026.08.21.746200; this version posted August 26, 2026. The copyright holder for this preprint (which was not certified by peer review) is the author/funder. This article is a US Government work. It is not subject to copyright under 17 USC 105 and is also made available for use under a CC0 license.

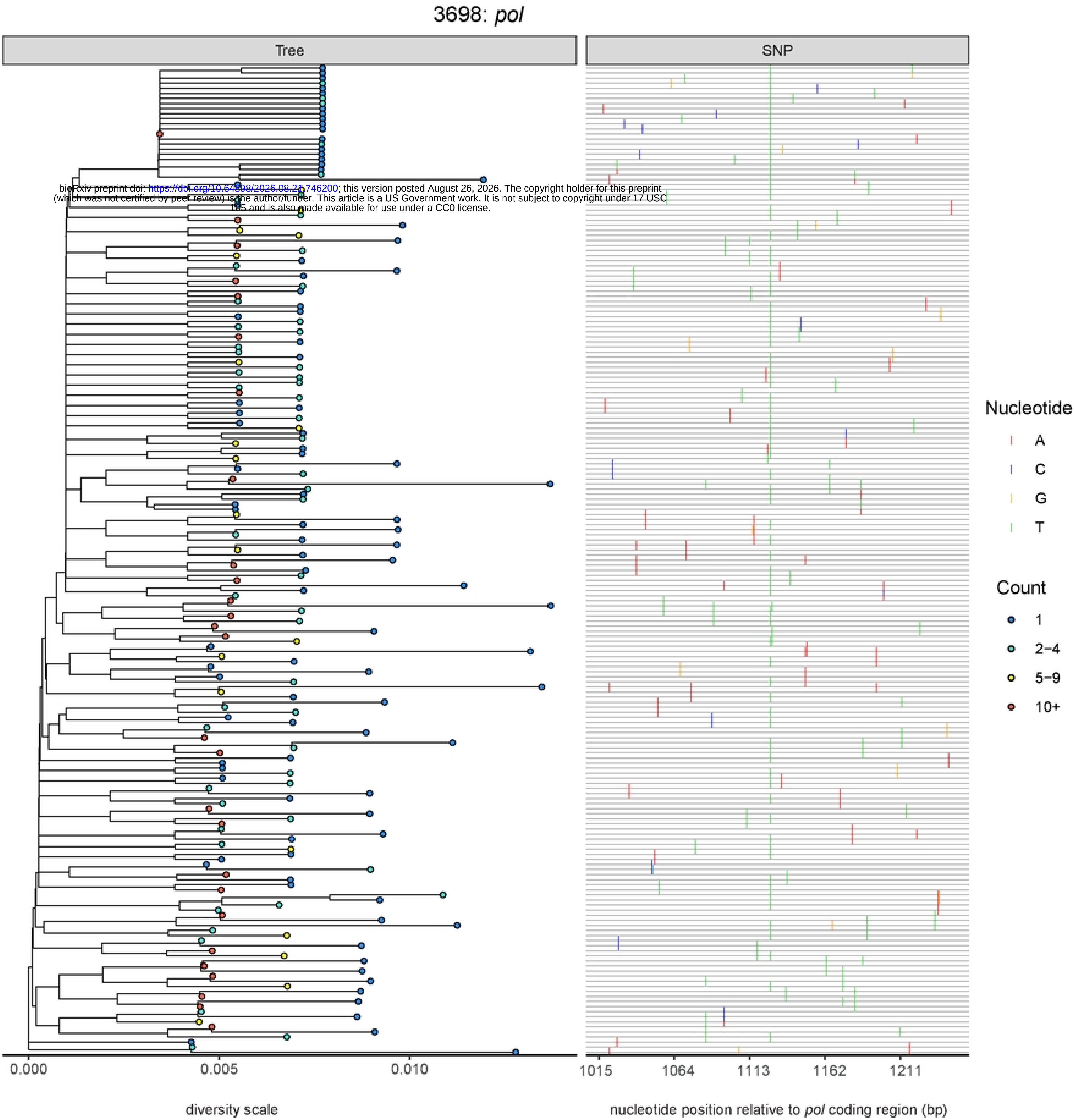

bioRxiv preprint doi: https://doi.org/10.64898/2026.08.21.746200; this version posted August 26, 2026. The copyright holder for this preprint (which was not certified by peer review) is the author/funder. This article is a US Government work. It is not subject to copyright under 17 USC 105 and is also made available for use under a CC0 license.

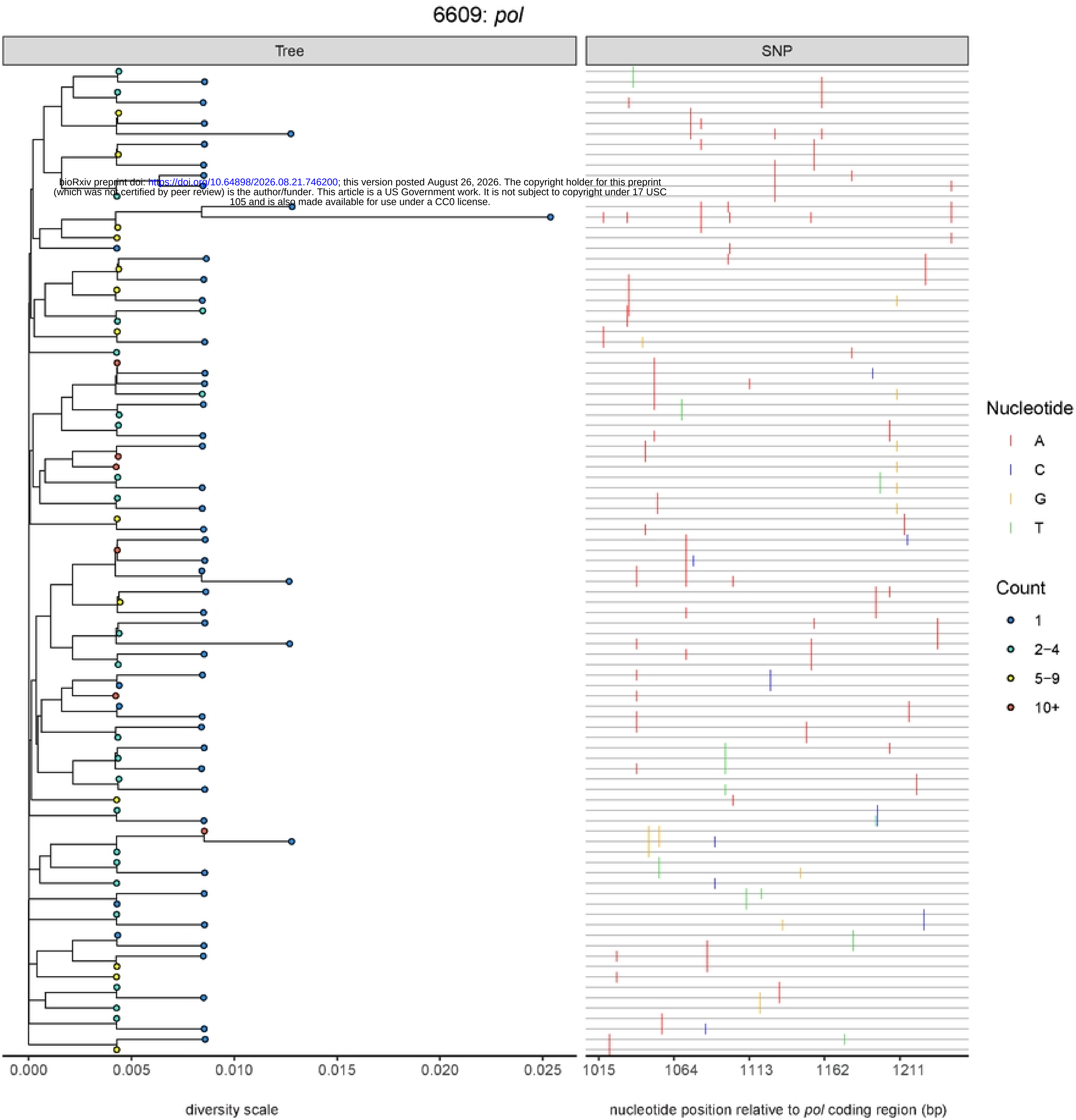

bioRxiv preprint doi: https://doi.org/10.64898/2026.08.21.746200; this version posted August 26, 2026. The copyright holder for this preprint (which was not certified by peer review) is the author/funder. This article is a US Government work. It is not subject to copyright under 17 USC 105 and is also made available for use under a CC0 license.

bioRxiv preprint doi: https://doi.org/10.64898/2026.08.21.746200; this version posted August 26, 2026. The copyright holder for this preprint (which was not certified by peer review) is the author/funder. This article is a US Government work. It is not subject to copyright under 17 USC 105 and is also made available for use under a CC0 license.

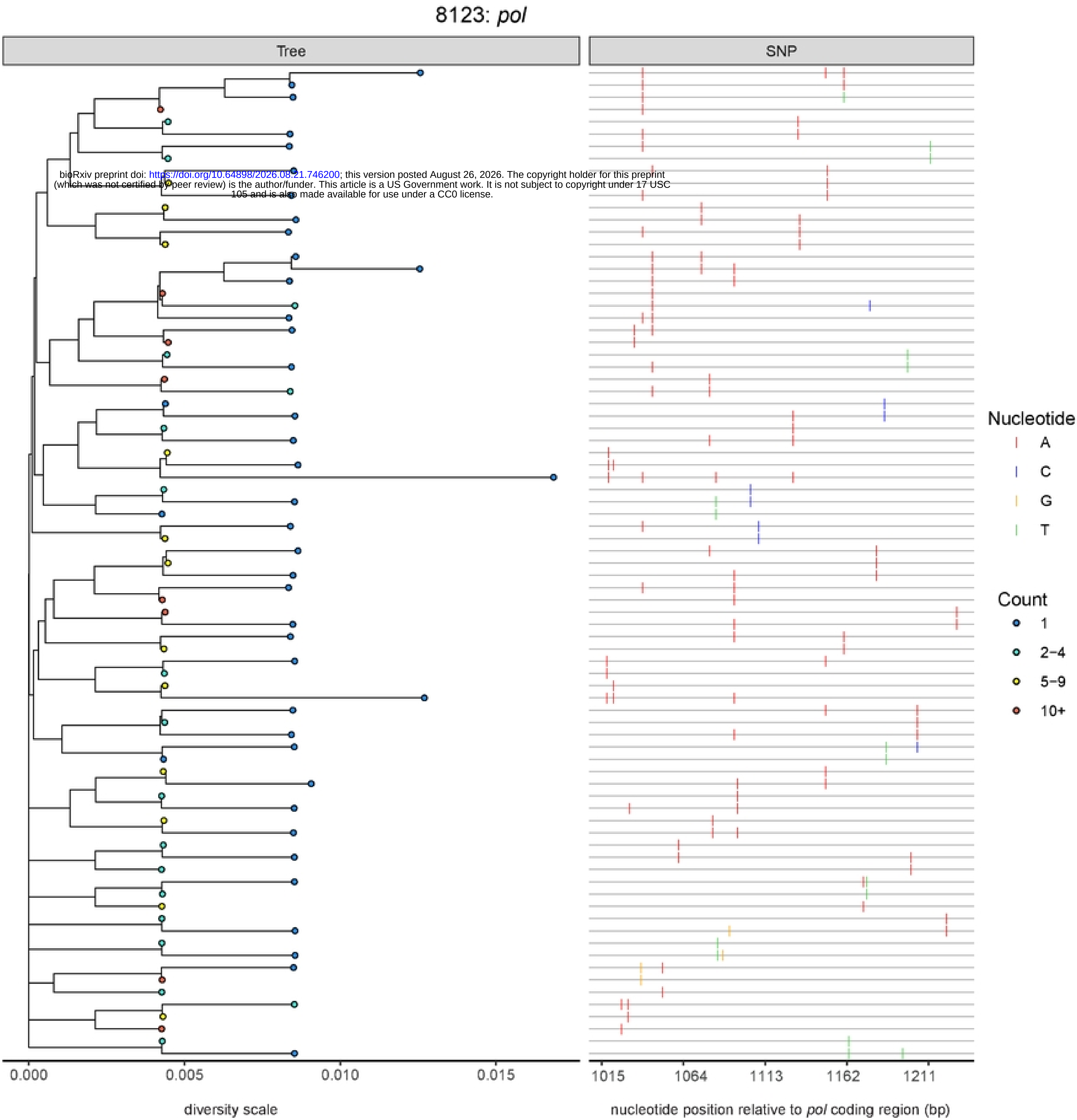

bioRxiv preprint doi: https://doi.org/10.64898/2026.08.21.746200; this version posted August 26, 2026. The copyright holder for this preprint (which was not certified by peer review) is the author/funder. This article is a US Government work. It is not subject to copyright under 17 USC 105 and is also made available for use under a CC0 license.

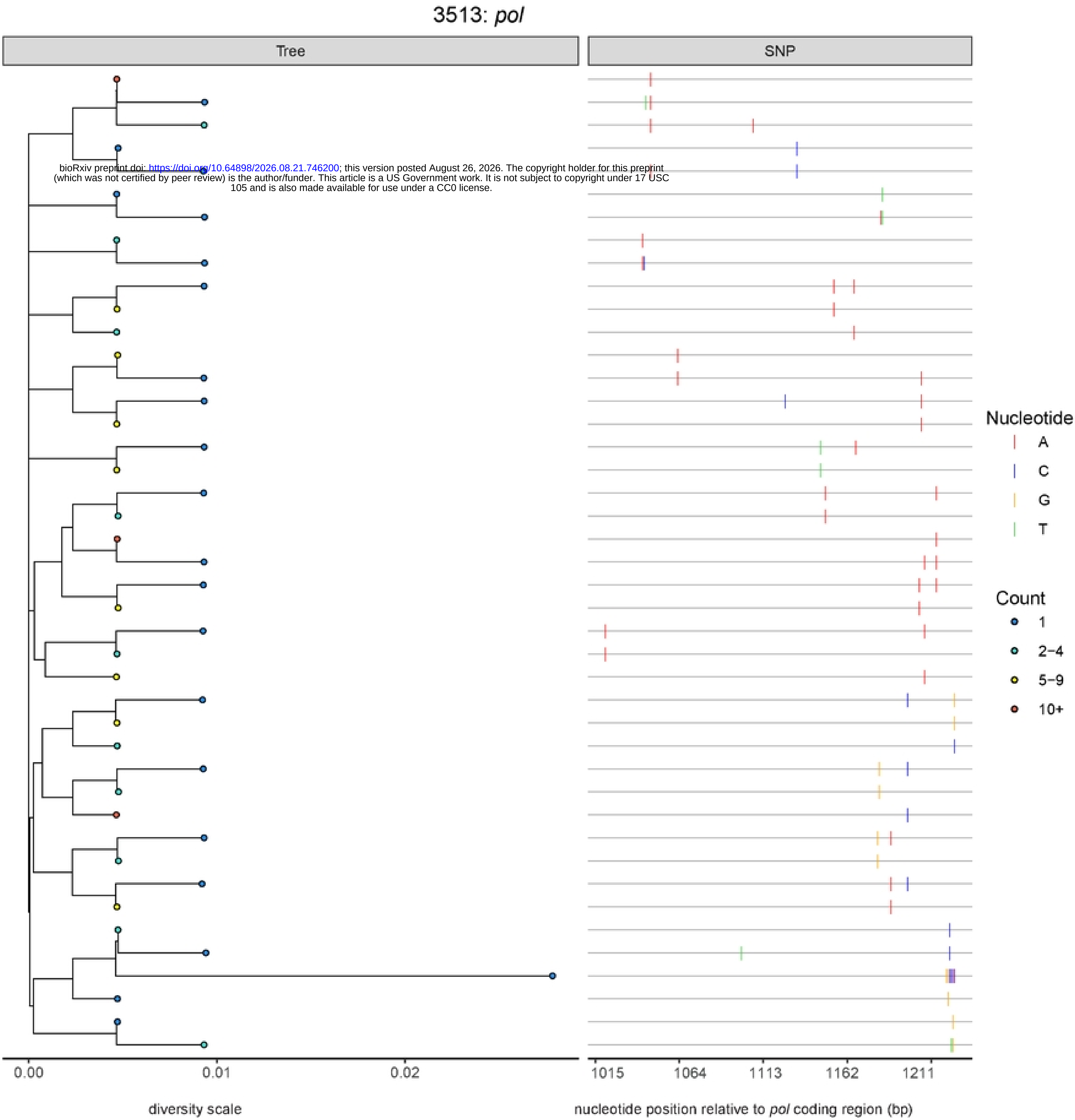

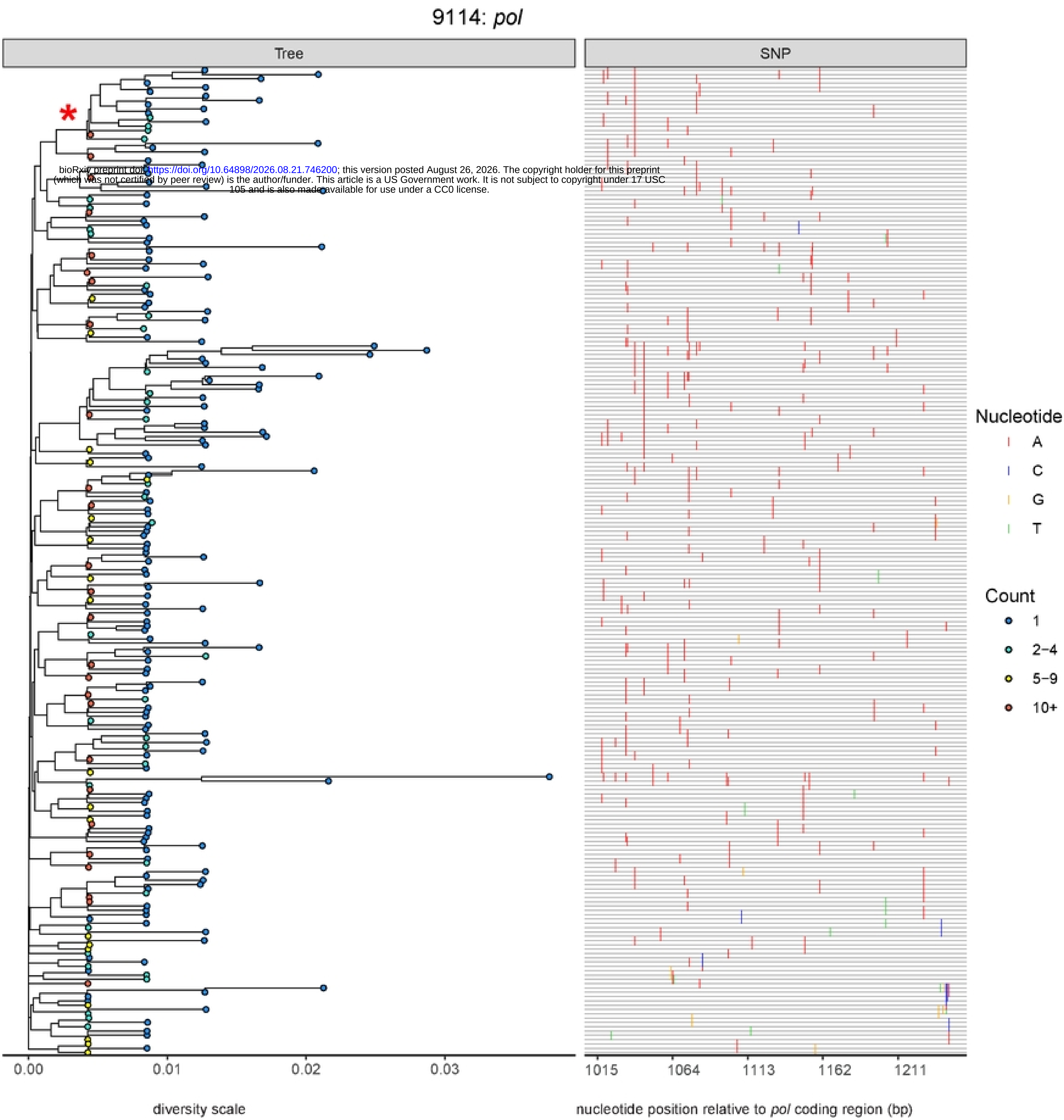

bioRxiv preprint doi: https://doi.org/10.64898/2026.08.21.746200; this version posted August 26, 2026. The copyright holder for this preprint (which was not certified by peer review) is the author/funder. This article is a US Government work. It is not subject to copyright under 17 USC 105 and is also made available for use under a CC0 license.

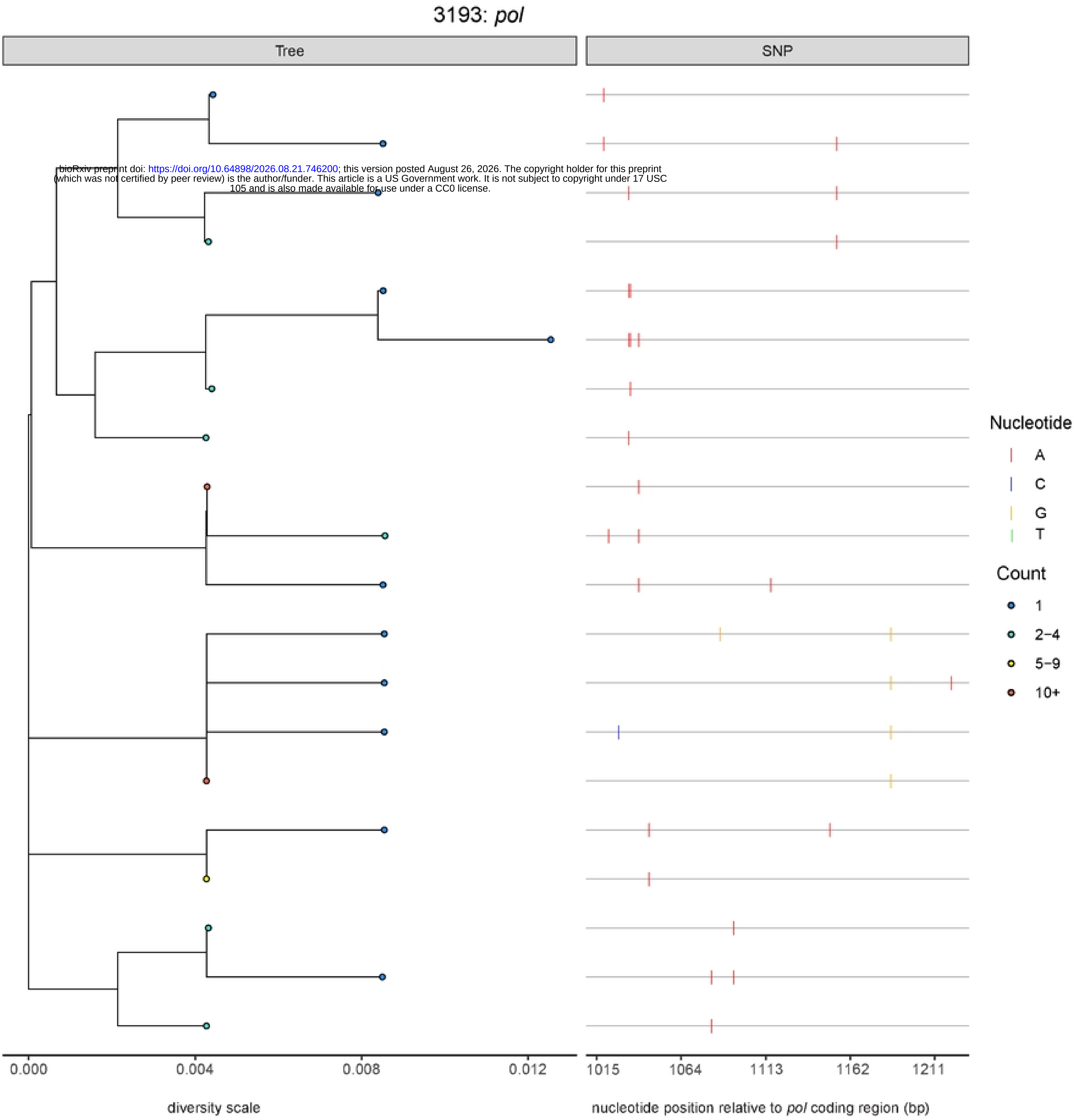

bioRxiv preprint doi: https://doi.org/10.64898/2026.08.21.746200; this version posted August 26, 2026. The copyright holder for this preprint (which was not certified by peer review) is the author/funder. This article is a US Government work. It is not subject to copyright under 17 USC 105 and is also made available for use under a CC0 license.

bioRxiv preprint doi: https://doi.org/10.64898/2026.08.21.746200; this version posted August 26, 2026. The copyright holder for this preprint (which was not certified by peer review) is the author/funder. This article is a US Government work. It is not subject to copyright under 17 USC 105 and is also made available for use under a CC0 license.

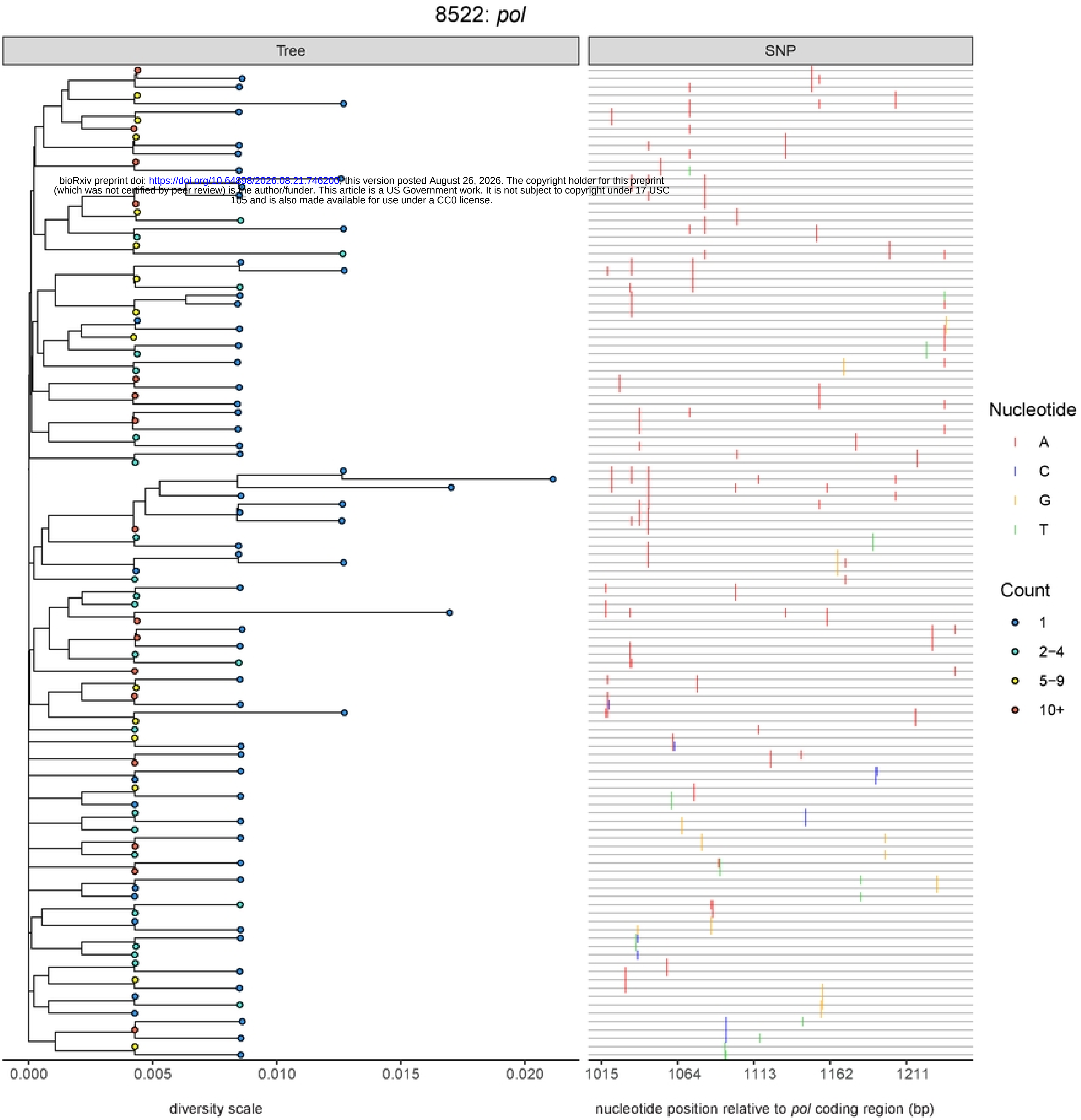

bioRxiv preprint doi: https://doi.org/10.64898/2026.08.21.746200; this version posted August 26, 2026. The copyright holder for this preprint (which was not certified by peer review) is the author/funder. This article is a US Government work. It is not subject to copyright under 17 USC 105 and is also made available for use under a CC0 license.

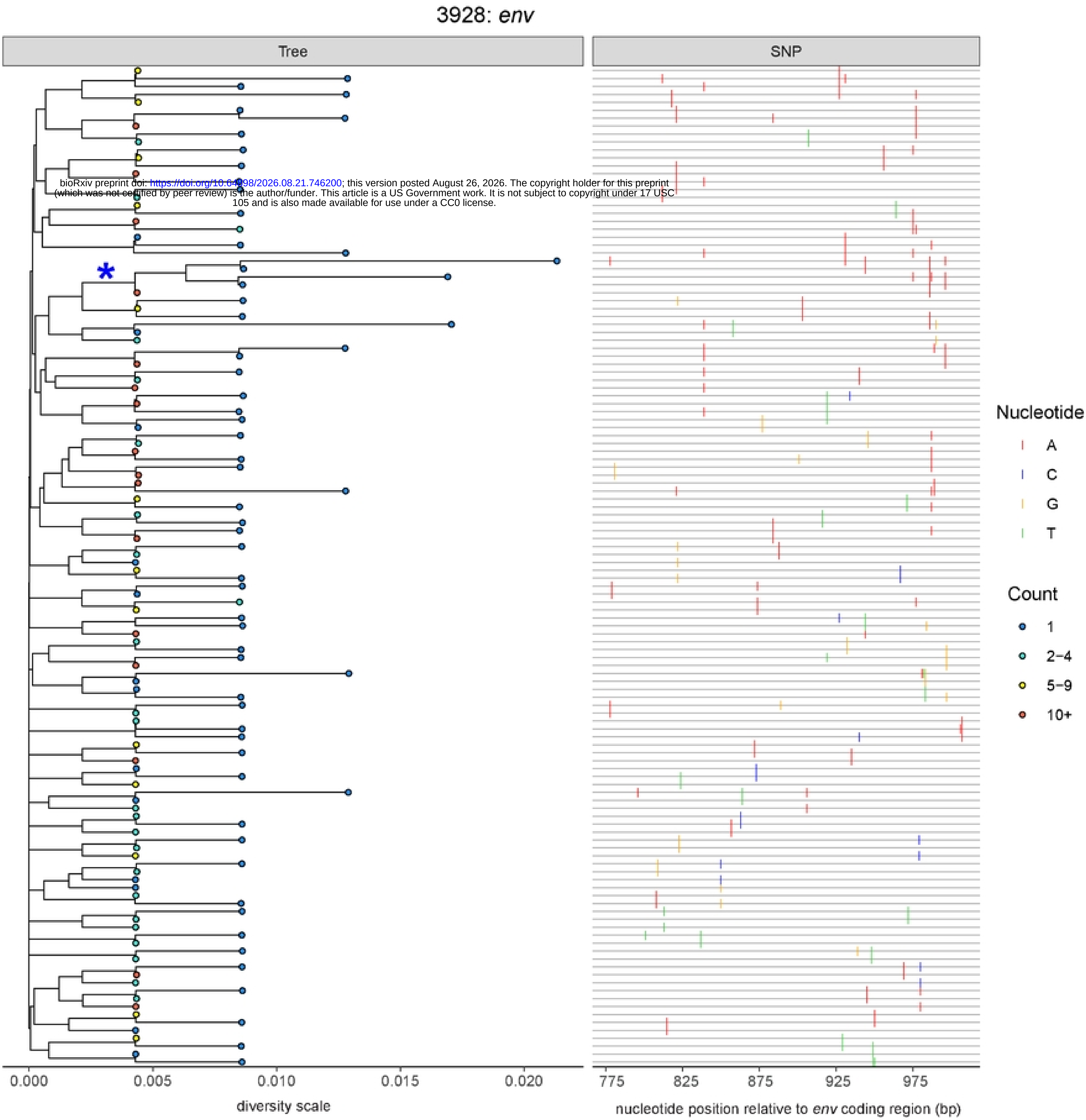

bioRxiv preprint doi: https://doi.org/10.64898/2026.08.21.746200; this version posted August 26, 2026. The copyright holder for this preprint (which was not certified by peer review) is the author/funder. This article is a US Government work. It is not subject to copyright under 17 USC 105 and is also made available for use under a CC0 license.

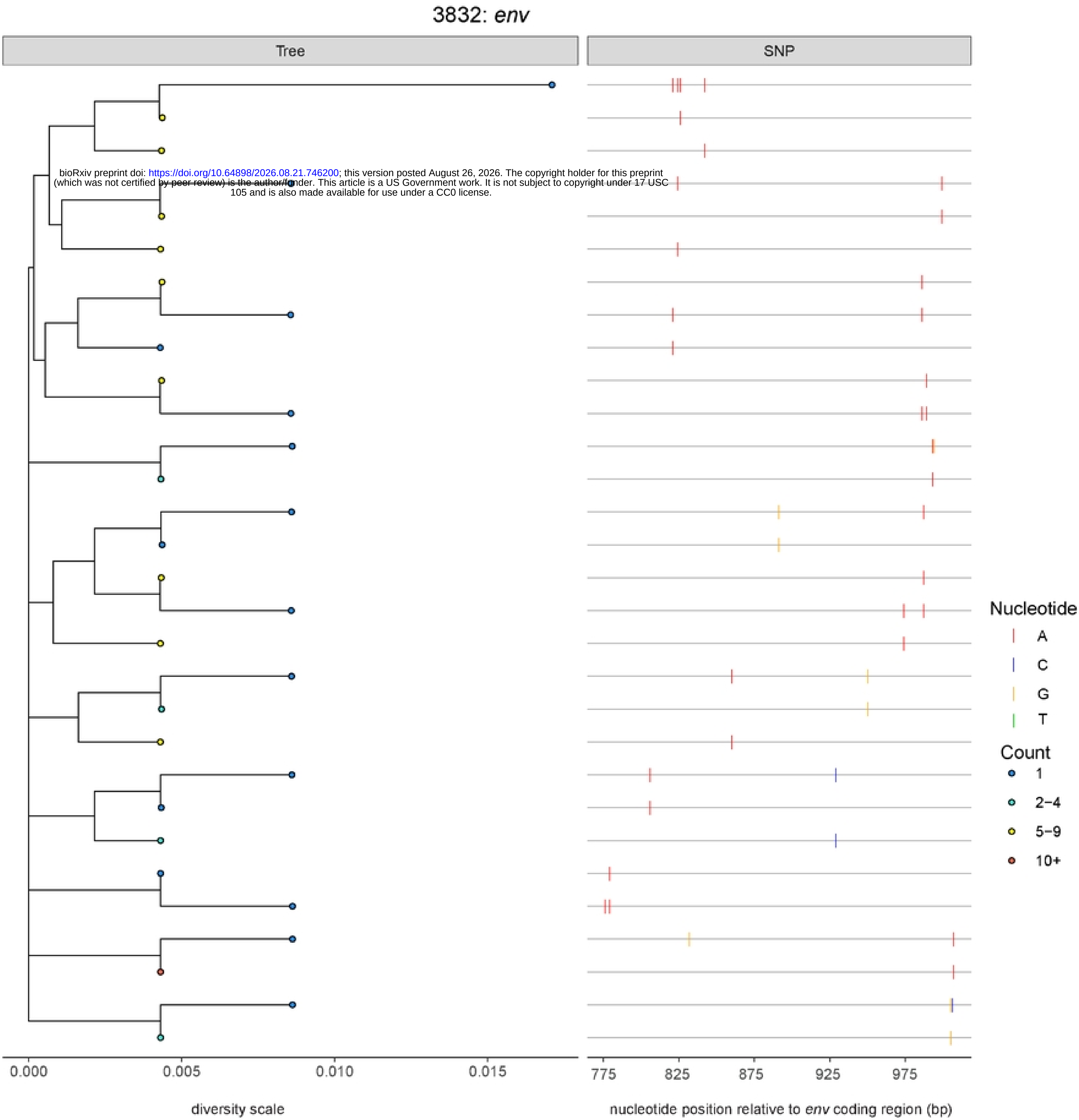

bioRxiv preprint doi: https://doi.org/10.64898/2026.08.21.746200; this version posted August 26, 2026. The copyright holder for this preprint (which was not certified by peer review) is the author/funder. This article is a US Government work. It is not subject to copyright under 17 USC 105 and is also made available for use under a CC0 license.

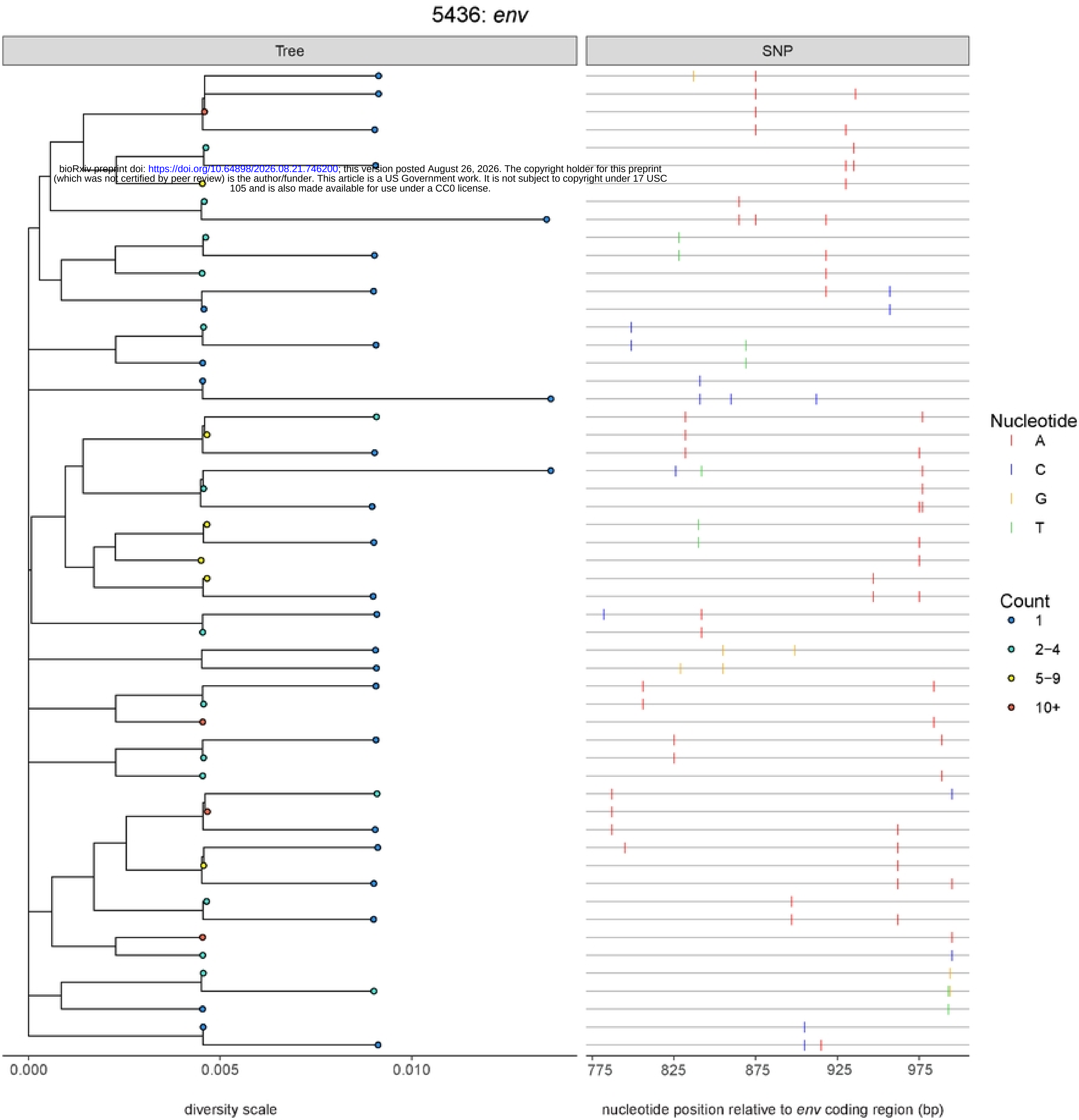

bioRxiv preprint doi: https://doi.org/10.64898/2026.08.21.746200; this version posted August 26, 2026. The copyright holder for this preprint (which was not certified by peer review) is the author/funder. This article is a US Government work. It is not subject to copyright under 17 USC 105 and is also made available for use under a CC0 license.

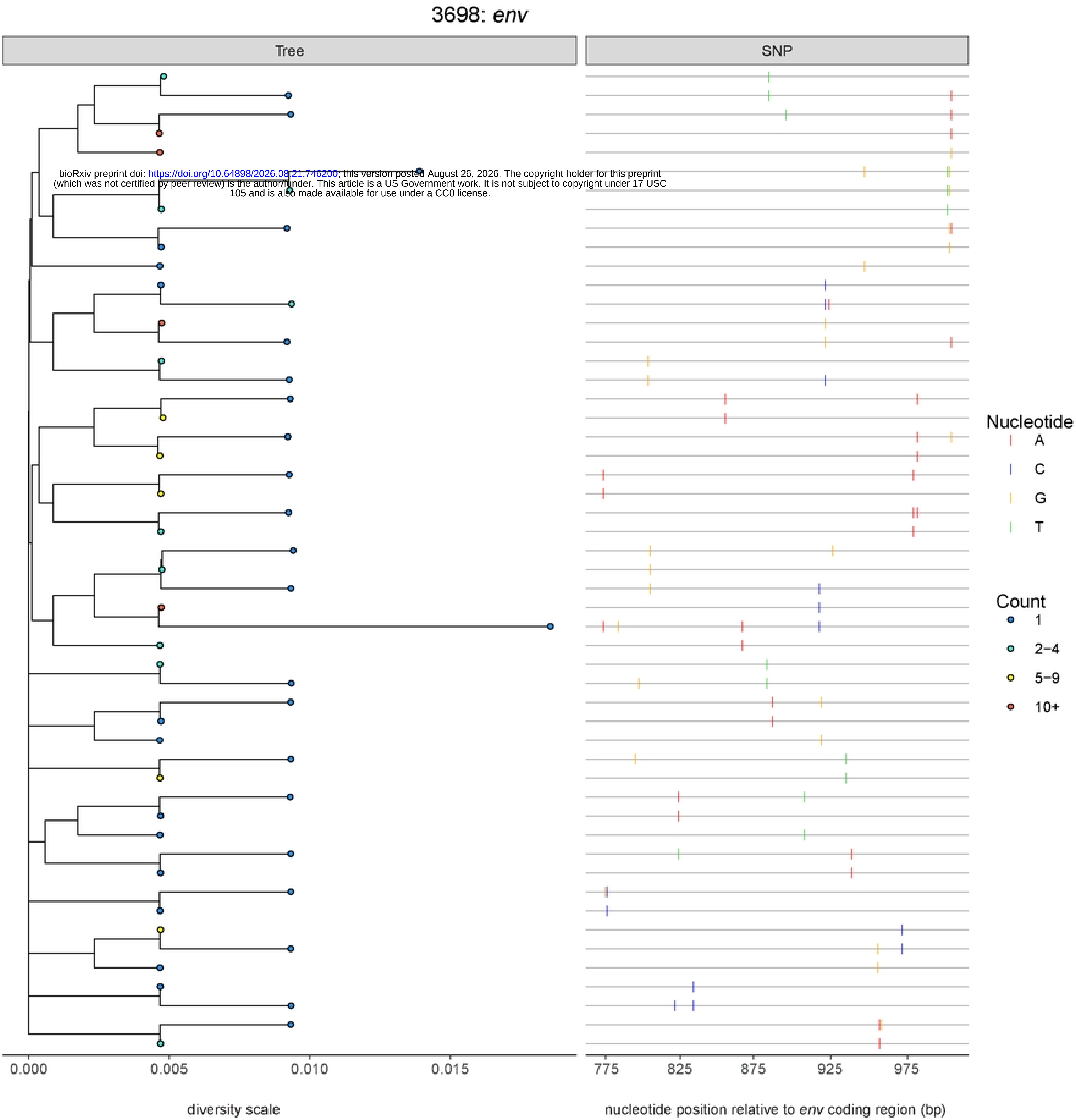

bioRxiv preprint doi: https://doi.org/10.64898/2026.08.21.746200; this version posted August 26, 2026. The copyright holder for this preprint (which was not certified by peer review) is the author/funder. This article is a US Government work. It is not subject to copyright under 17 USC 105 and is also made available for use under a CC0 license.

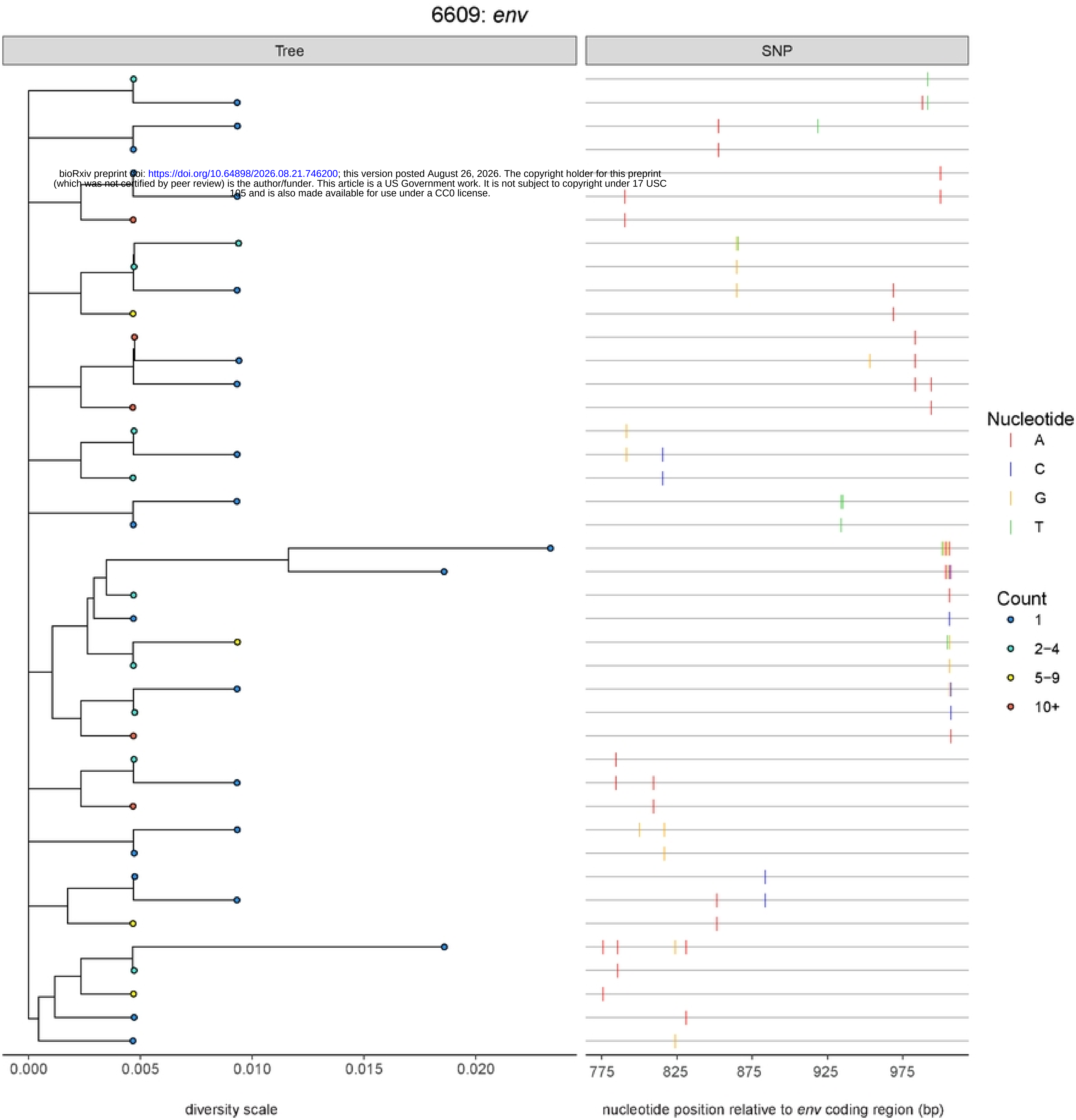

bioRxiv preprint doi: https://doi.org/10.64898/2026.08.21.746200; this version posted August 26, 2026. The copyright holder for this preprint (which was not certified by peer review) is the author/funder. This article is a US Government work. It is not subject to copyright under 17 USC 105 and is also made available for use under a CC0 license.

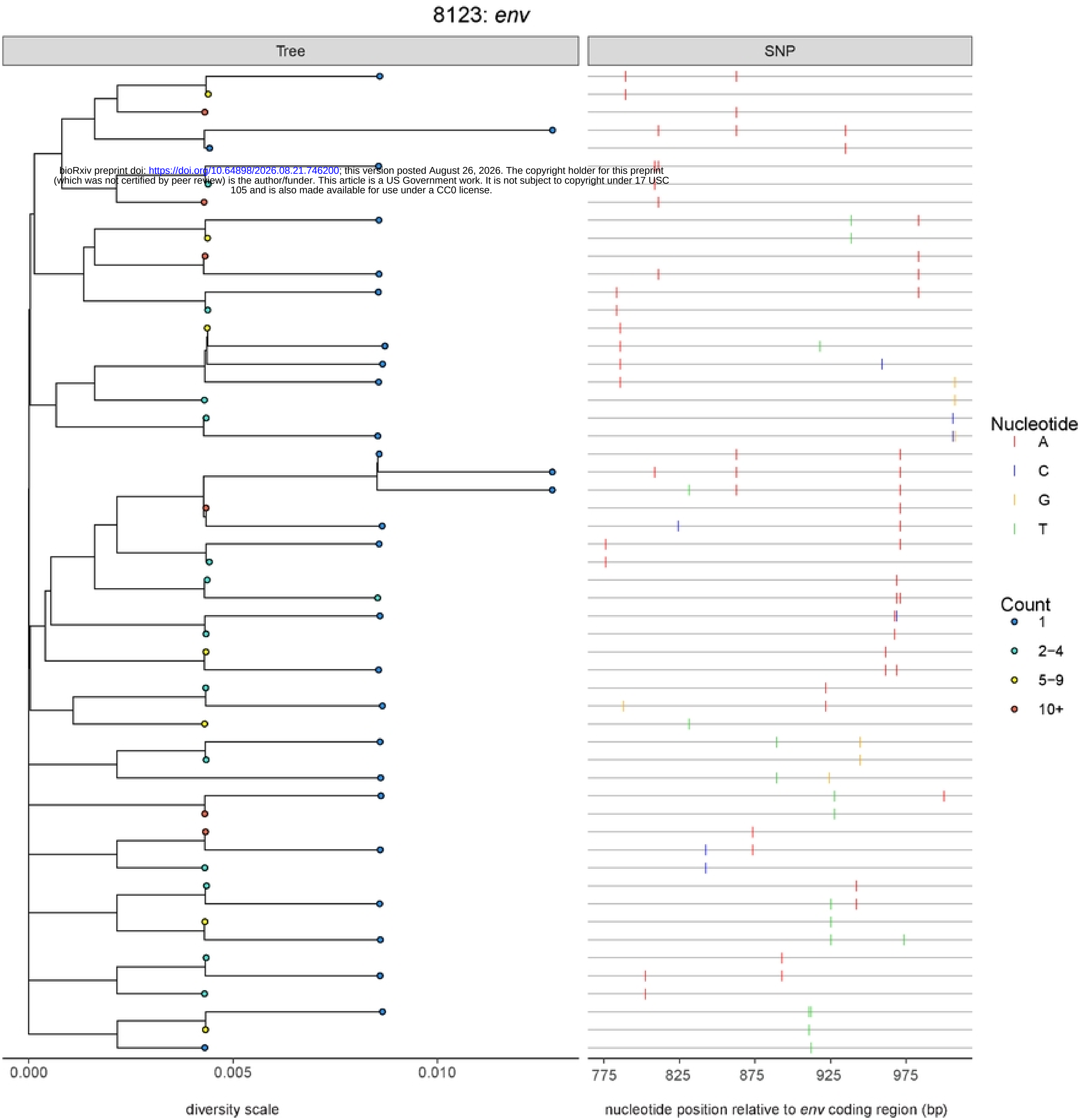

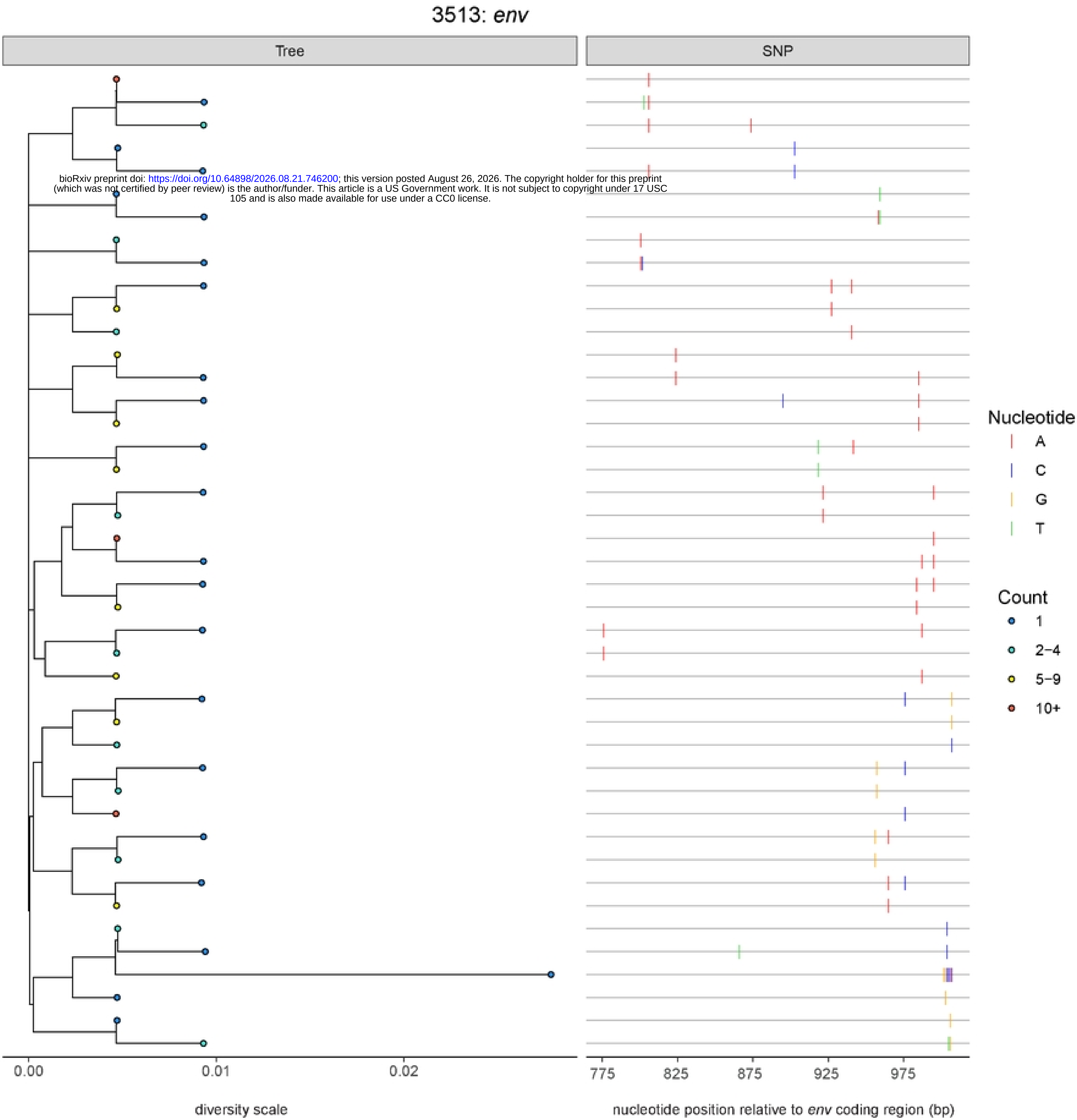

bioRxiv preprint doi: https://doi.org/10.64898/2026.08.21.746200; this version posted August 26, 2026. The copyright holder for this preprint (which was not certified by peer review) is the author/funder. This article is a US Government work. It is not subject to copyright under 17 USC 105 and is also made available for use under a CC0 license.

bioRxiv preprint doi: https://doi.org/10.64898/2026.08.21.746200; this version posted August 26, 2026. The copyright holder for this preprint (which was not certified by peer review) is the author/funder. This article is a US Government work. It is not subject to copyright under 17 USC 105 and is also made available for use under a CC0 license.

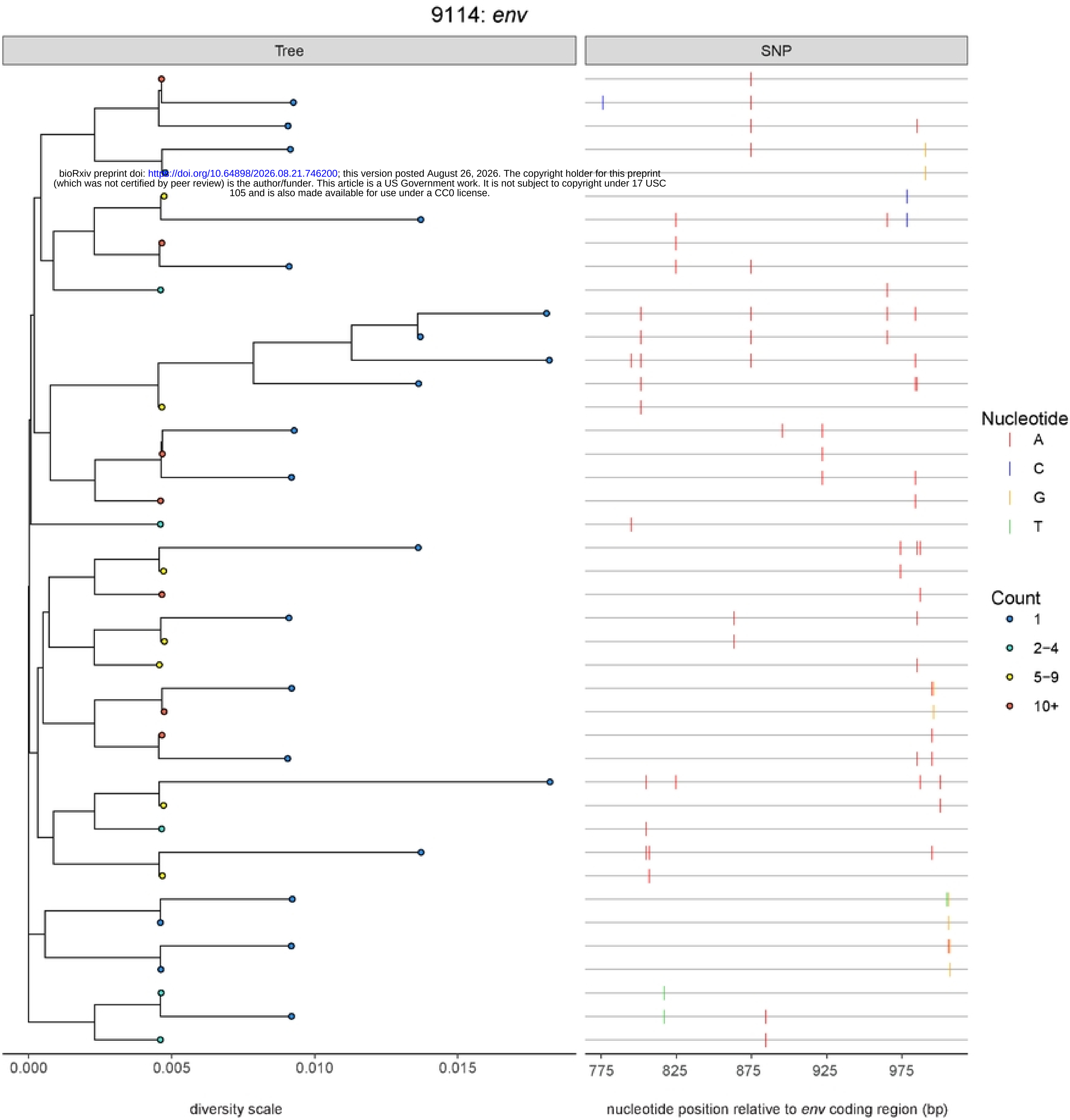

bioRxiv preprint doi: https://doi.org/10.64898/2026.08.21.746200; this version posted August 26, 2026. The copyright holder for this preprint (which was not certified by peer review) is the author/funder. This article is a US Government work. It is not subject to copyright under 17 USC 105 and is also made available for use under a CC0 license.

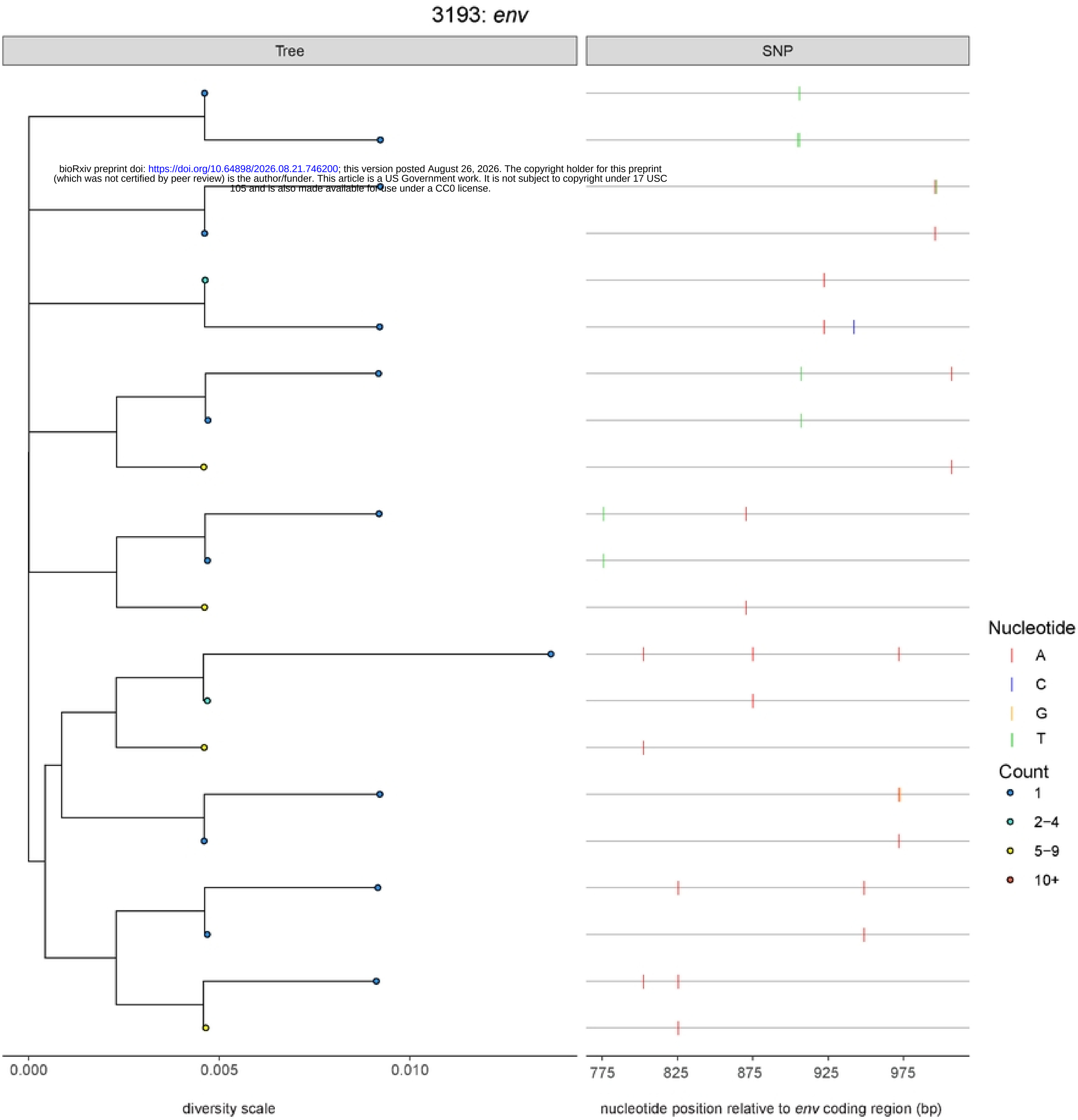

bioRxiv preprint doi: https://doi.org/10.64898/2026.08.21.746200; this version posted August 26, 2026. The copyright holder for this preprint (which was not certified by peer review) is the author/funder. This article is a US Government work. It is not subject to copyright under 17 USC 105 and is also made available for use under a CC0 license.

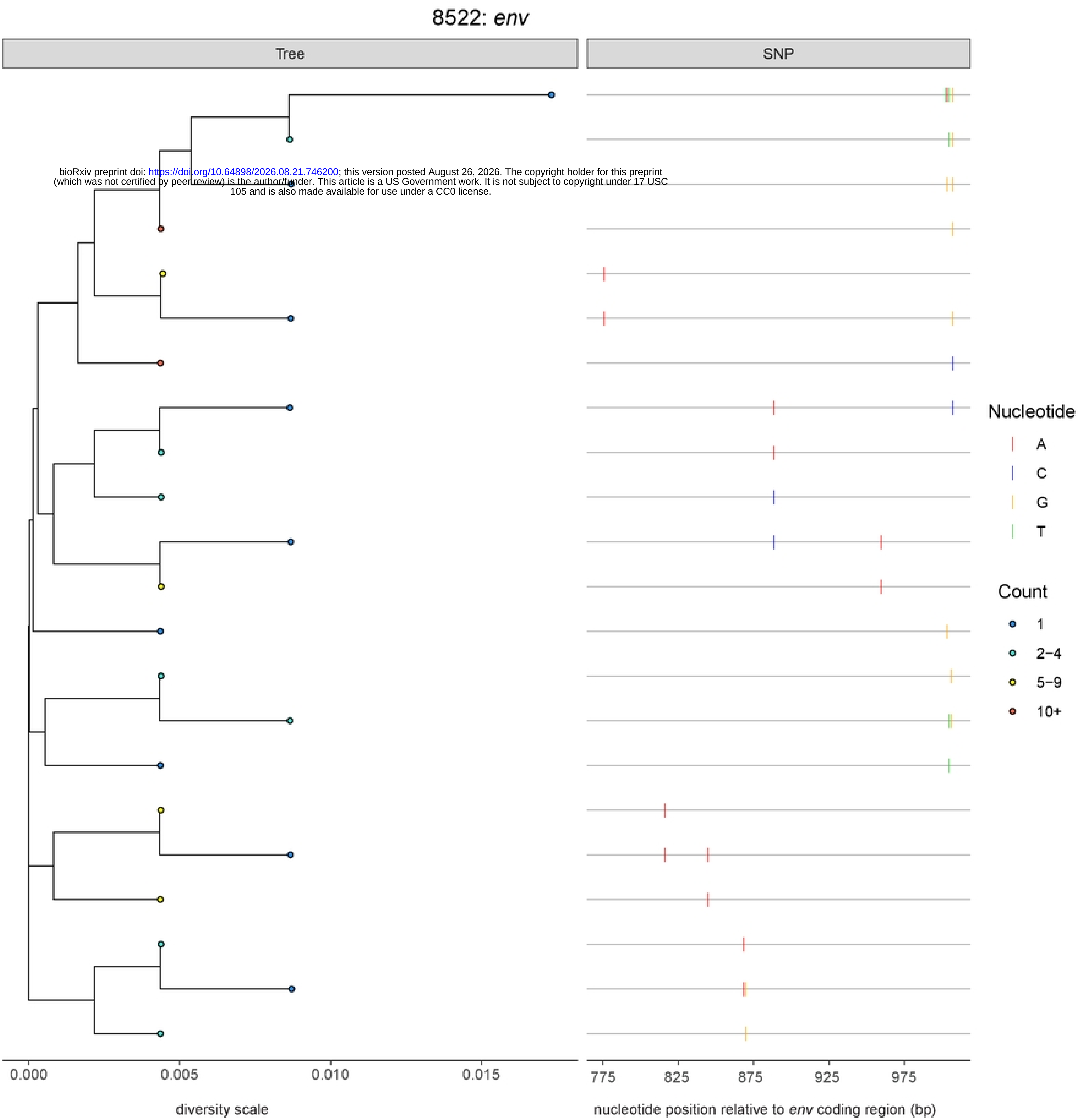

Figure S8

bioRxiv preprint doi: https://doi.org/10.64898/2026.08.21.746200; this version posted August 26, 2026. The copyright holder for this preprint (which was not certified by peer review) is the author/funder. This article is a US Government work. It is not subject to copyright under 17 USC 105 and is also made available for use under a CC0 license.

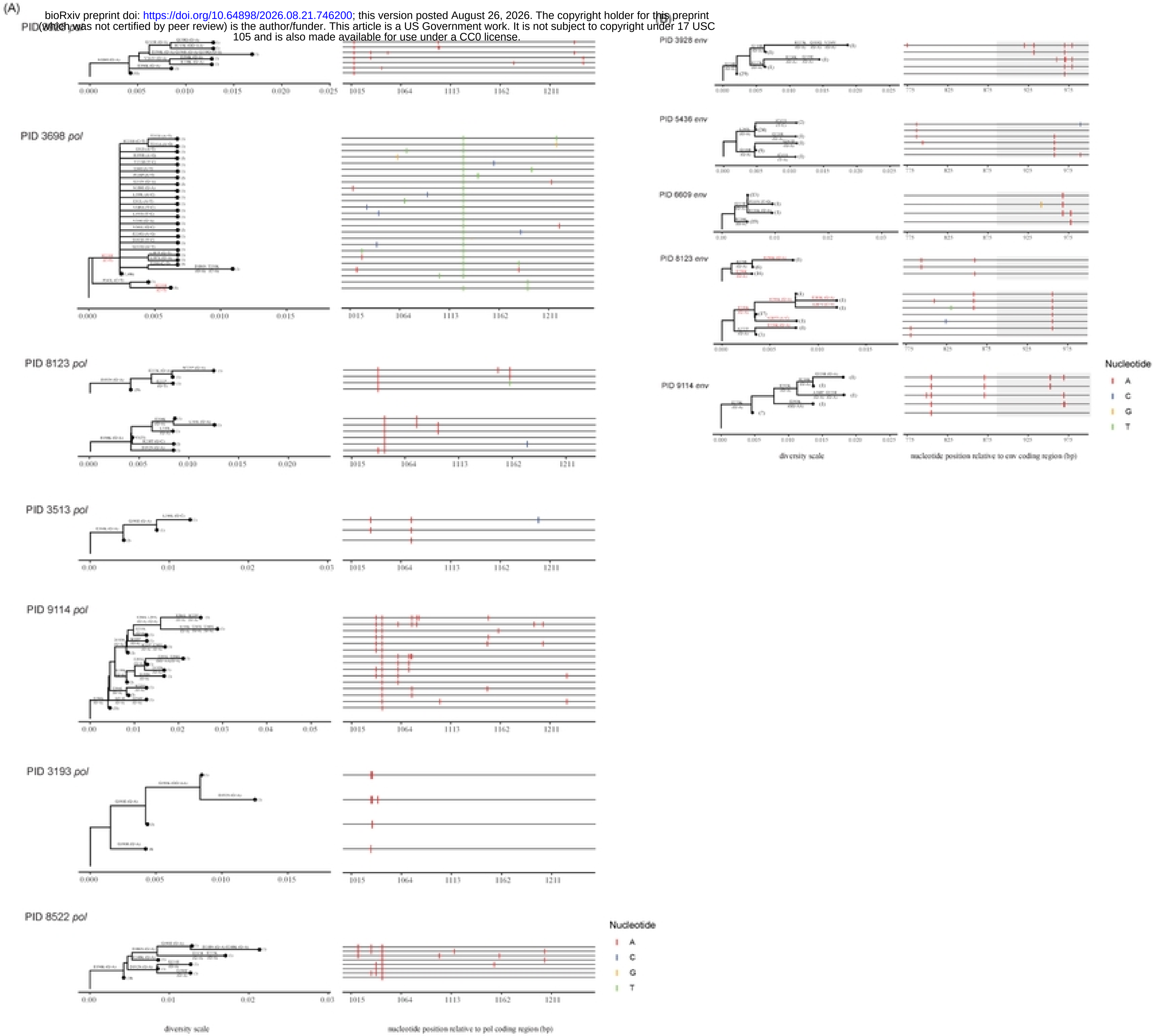

Figure S9

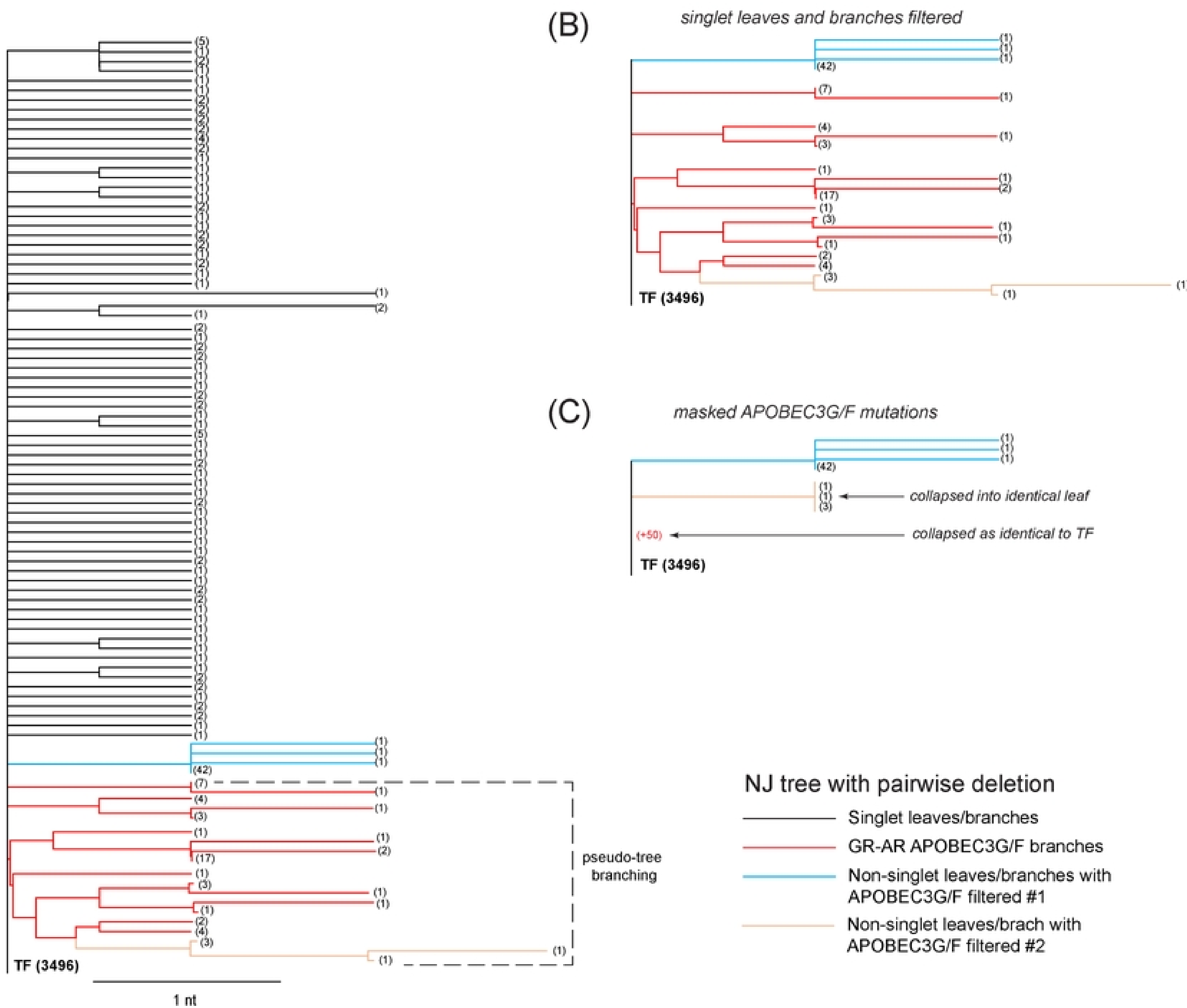

(B)
singlet leaves and branches filtered
TF (3496)
(C)
masked APOBEC3G/F mutations
collapsed into identical leaf
(+50)
collapsed as identical to TF
TF (3496)
pseudo-tree branching
TF (3496)
1 nt
NJ tree with pairwise deletion
Singlet leaves/branches
GR-AR APOBEC3G/F branches
Non-singlet leaves/branches with APOBEC3G/F filtered #1
Non-singlet leaves/brach with APOBEC3G/F filtered #2

Figure S10

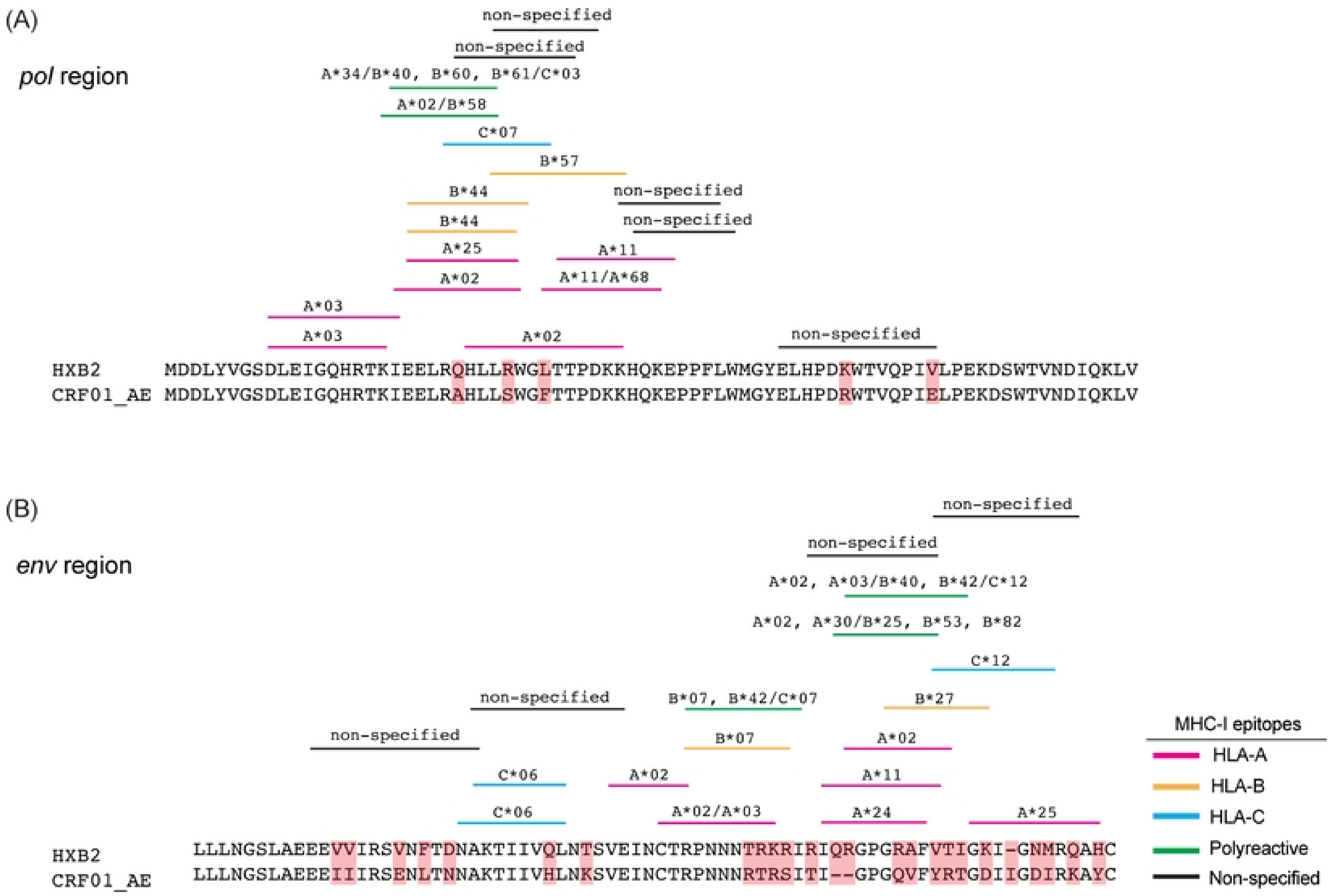

(A)
pol region
non-specified
non-specified
A*34/B*40, B*60, B*61/C*03
A*02/B*58
C*07
B*57
B*44
non-specified
B*44
non-specified
A*25
A*11
A*02
A*11/A*68
A*03
A*03
A*02
non-specified
HXB2 MDDLYVGSDLEIGQHRTKIEELRQHLLRWGLTTPDKKHQKEPPFLWMGYELHPDKWTVQPIVLPEKDSWTVNDIQKLV
CRF01_AE MDDLYVGSDLEIGQHRTKIEELRAHLLSWGFTTPDKKHQKEPPFLWMGYELHPDRWTVQPIELPEKDSWTVNDIQKLV
(B)
env region
non-specified
non-specified
A*02, A*03/B*40, B*42/C*12
A*02, A*30/B*25, B*53, B*82
C*12
non-specified
B*07, B*42/C*07
B*27
non-specified
B*07
A*02
C*06
A*02
A*11
C*06
A*02/A*03
A*24
A*25
HXB2 LLLNGSLAEEEVVIRSVNFTDNAKTIIVQLNTSVEINCTRPNNNTRKRIRIQRGPGRAFVTIGKI-GNMRQAHC
CRF01_AE LLLNGSLAEEEIIIRSENLTNNAKTIIVHLNKSVEINCTRPNNNRTRSITI--GPGQVFYRTGDIIGDIRKAYC
MHC-I epitopes
HLA-A
HLA-B
HLA-C
Polyreactive
Non-specified

Figure S11

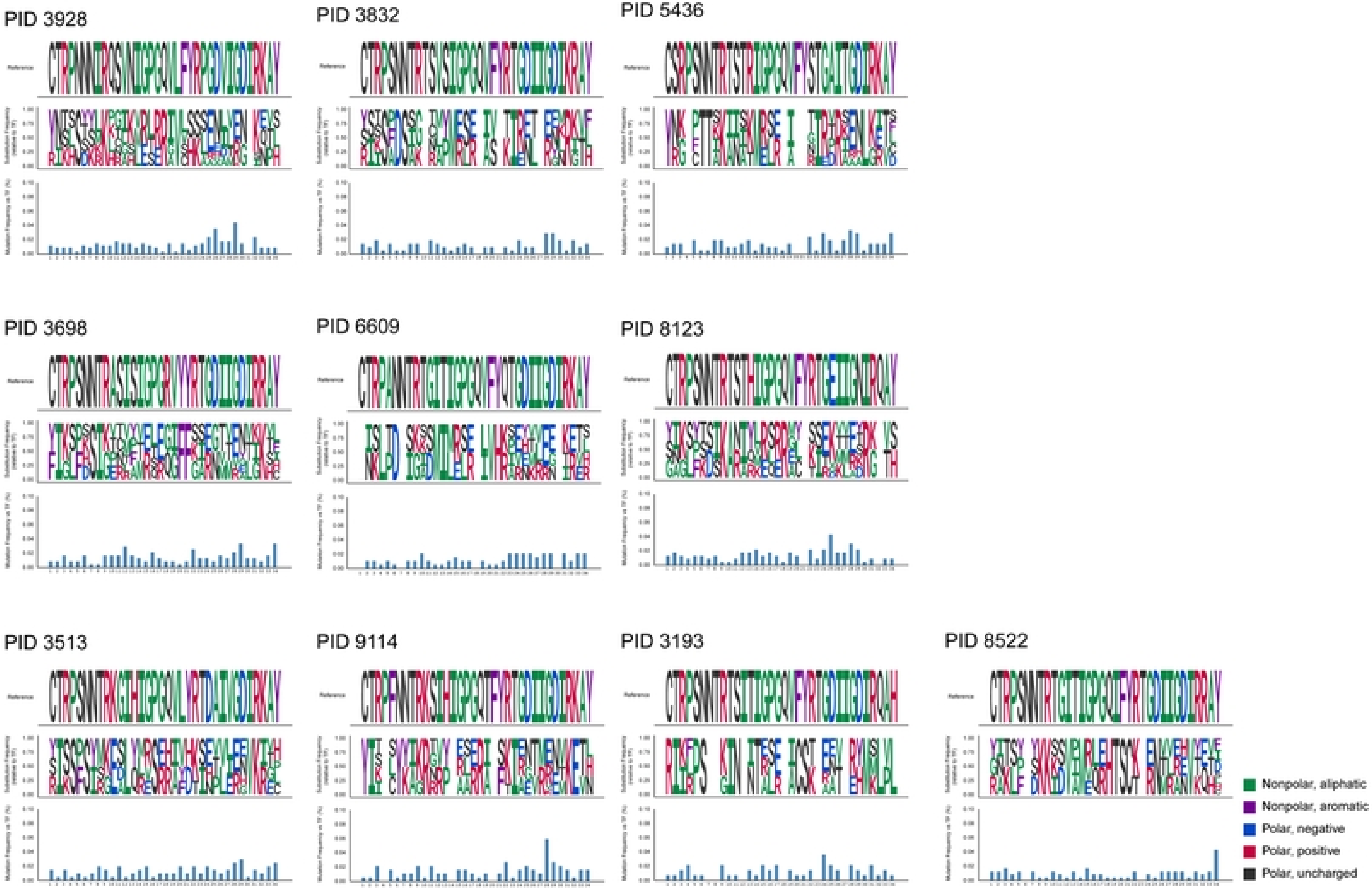
PID 3928
PID 3832
PID 5436
PID 3698
PID 6609
PID 8123
PID 3513
PID 9114
PID 3193
PID 8522
Nonpolar, aliphatic
Nonpolar, aromatic
Polar, negative
Polar, positive
Polar, uncharged

Figure S12

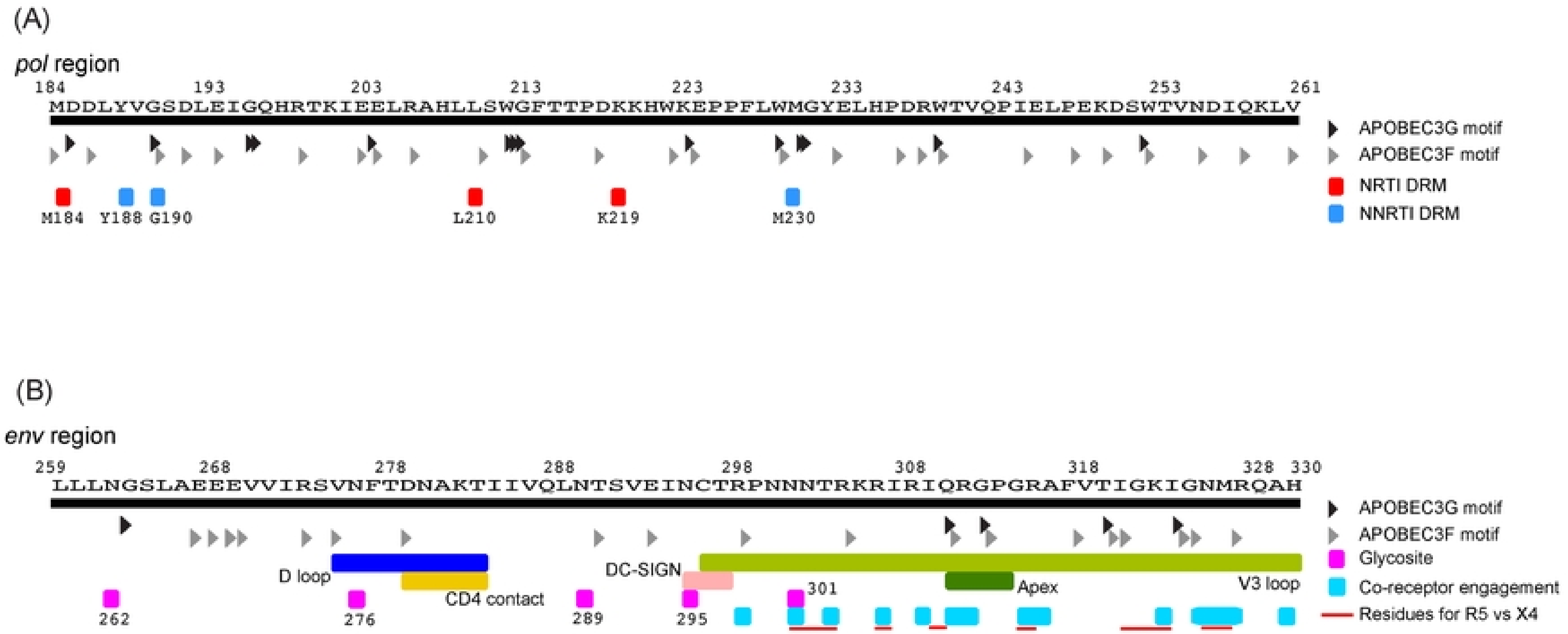

(A)
pol region
184
193
203
213
223
233
243
253
261
MDDLYVGSDLEIGQHRTKIEELRAHLLSWGFTTPDKKHWKEPPFLWMGYELHPDRWTVQPIELPEKDSWTVNDIQKLV
APOBEC3G motif
APOBEC3F motif
NRTI DRM
NNRTI DRM
M184
Y188
G190
L210
K219
M230
(B)
env region
259
268
278
288
298
308
318
328
330
LLLNGSLAEEEVVIRSVNFTDNAKTIIVQLNTSVEINCTRPNNNTRKRIRIQRGPGRAFVTIGKIGNMRQAH
APOBEC3G motif
APOBEC3F motif
Glycosite
Co-receptor engagement
Residues for R5 vs X4
D loop
CD4 contact
DC-SIGN
Apex
V3 loop
262
276
289
295
301
